# Green One-Pot Syntheses of 2-Sulfoximidoyl-3,6-dibromo Indoles Using *N*-Br Sulfoximines as Both Brominating and Sulfoximinating Reagents

**DOI:** 10.3390/molecules28083380

**Published:** 2023-04-11

**Authors:** Xiao Yun Chen, Yaonan Tang, Xinran Xiang, Yisong Tang, Mingyang Huang, Shaojun Zheng, Cuifeng Yang

**Affiliations:** 1 School of Environmental and Chemical Engineering, Jiangsu University of Science and Technology, Zhenjiang 212003, China; 2 Modern Chemistry Research Institute of Xi’an, Xi’an 710065, China; 3 State Key Laboratory of Fluorine & Nitrogen Chemicals, Xi’an 710065, China

**Keywords:** 2,3,6-trifunctionalization of indoles, *N*-Br sulfoximines, brominating reagent, sulfoximinating reagent

## Abstract

A green one-pot 2,3,6-trifunctionalization of *N*-alkyl/aryl indoles was achieved by adding three equivalents of *N*-Br sulfoximine to the indole solution. A variety of 2-sulfoximidoyl-3,6-dibromo indoles were prepared with 38–94% yields using *N*-Br sulfoximines as both brominating and sulfoximinating reagents. Based on the results of controlled experiments, we propose that a radical substitution involving 3,6-dibromination and 2-sulfoximination occurs in the reaction process. This is first time that 2,3,6-trifunctionalization of indole in one pot has been achieved.

## 1. Introduction

**Scheme 1:**
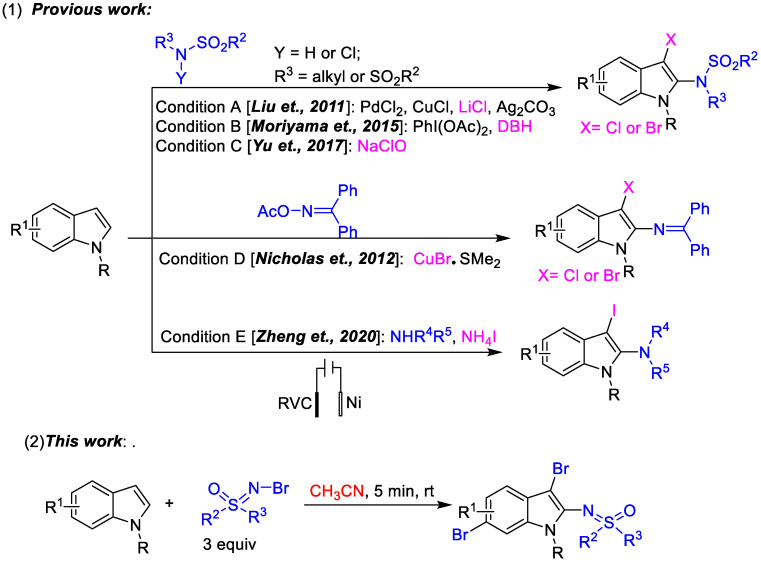
(1) The reported aminohologenation of indoles by using different aminating reagent [33,34,35,36,38]; (2) The green one-pot 2,3,6- trifunctinalization of indoles by using N-Br sulfoximines as both of brominating and aminating reagents.

The indole moiety is always attractive because of its existence in a wide range of natural products and bioactive compounds as core structures [1,2,3,4,5,6]. Hence, the development of simple and green methods for the synthesis of multisubstituted indole derivatives is an important research area. Sulfoximine derivatives, a class of sulfur-containing compounds, have attracted considerable interest for their diverse applications in organic synthesis as building blocks [7,8], in asymmetric catalysis as ligands and chiral auxiliary [9,10,11] and in pharmacology as bioactive components [12,13,14]. A simple combination of indoles and sulfoximine maybe enable the facile synthesis of a series of potentially bioactive sulfoximidoyl indoles. The successful development of the new protocol will facilitate relative research based on sulfoximines and indoles. Normally, the direct functionalization, especially multifunctionalization, of indoles via C-H activation is an atom- and step-economical approach to expand the library of indoles [15,16,17,18,19,20,21,22,23,24,25,26]. Compared to the wildly known C2- and C3-functionalization of indoles [15,16,17,18,19,20,21], the practicality of transformation on other positions is decreased and more difficult. Due to the significant advances in the development of synthetic methodologies, various dual-functionalization strategies of indoles have been developed to introduce two functional groups at the common C2 and C3 positions of the indole core simultaneously [27,28,29,30,31,32]. Among them, effective one-step dehydrogenative aminohalogenation of indoles is always a research hotspot because of the further application of aminohalogenated indoles in the fields of organic synthesis and biology. Despite several excellent works having been reported in the last decade (Scheme 1) [33,34,35,36,37,38], the field of dehydrogenative aminohalogenation of indoles remains largely unexplored. The above studies, pioneering aminohalogenation of indoles, have included the aminohalogenation of *N*-R indoles using sulfonamides and its derivatives as aminating reagents [33,35,36], the copper-catalyzed aminobromination of indoles using CuBr as brominating reagent and active *N*-Br diphenylmethanimine as aminating reagent [34], the electrochemical aminoiodination of indoles using unactivated secondary amines [38]. Inspired by such dehydrogenative aminohalogenation of indoles, sulfoximines as a special aminating reagent entered our field of vision. As is known, due to our long-term research focus [39,40,41,42], the radical addition of olefins using *N*-X sulfoximines [43], and the curiosity about sulfoximine indoles, we wondered whether the active *N*-Br sulfoximines could be used as the aminating reagent or both brominating and aminating reagents to achieve the aminobromination of indoles. Fortunately, this was achieved, and 2,3,6-trifunctionalized indole derivatives were afforded accidentally in one step for the first time in the absence of any transition metal or additional additives (Scheme 1). Due to its step and atom economy, novelty, simplicity and practicality, this novel trifunctionalization of indoles represents important progress towards functionalized indoles.

## 2. Results and Discussion

### 2.1. Optimization

At the beginning of our investigation, the 5 mol% CuBr-catalyzed sulfoximidoylbromination of 1-methylindole (**1a**) using *N*-Br methyl phenyl sulfoximine (**2a**) as both brominating and aminating reagents was attempted in the presence of KOAc at room temperature (entry 1, Table S1). To our delight, the desired dual-functionalized product **4a** was obtained with 29% yield as expected. However, the yield could not be improved more than 29% by varying copper salts, the amount of catalyst, temperature, or reaction time during further optimization (entries 2–10, Table S1). In the reaction process, the *N*-Br methyl phenyl sulfoximine (**2a**) was found to be consumed quickly. As such, the amount of *N*-Br methyl phenyl sulfoximine (**2a**) was evaluated, the trifunctionalized product **3aa** was obtained suddenly with higher 76% isolated yield when the amount of **2a** was increased threefold (entries 11–17, Table S1). According to the above results, the catalyst and base seem to have little effect on the reaction compared to the amount of **2a**. Therefore, the reactions were tried in the absence of copper salts or cooperation of copper salts and KOAc, and **3aa** was produced with excellent 90% or 88% yield, respectively (entries 18–19, Table S1). The model reaction with 1 equiv. 1-methylindole (**1a**) and 3 equiv. *N*-Br methyl phenyl sulfoximine (**2a**) in the absence of transition metal and base was reset (Table 1). The desired trifunctionalized product **3aa** gave an excellent 94% isolated yield (entry 3, Table 1). Then, versatile solvents were evaluated in this simple protocol, and the desired product **3aa** was afforded by all test solvents, except toluene, in 28–80% yields (Entries 4–8, Table 1). The lowest 28% yield was given by the protic solvent EtOH, and the cyclic ether solvents THF and 1,4-dioxane gave good 80% and 72% yields, respectively. The compound **3aa** was also generated in moderate 64% yield using DCE as solvent. Finally, the optimum conditions of 2,3,6-trifunctionalization of indoles was chosen as 3 equiv. *N*-Br sulfoximines added to a solution of 1 equiv. indole derivatives (0.4 mmol) in 2 mL MeCN (entry 3, Table 1) with further stirring for 5 min at room temperature.

### 2.2. Extending the Scope of Indole Substrates

**Scheme 2:**
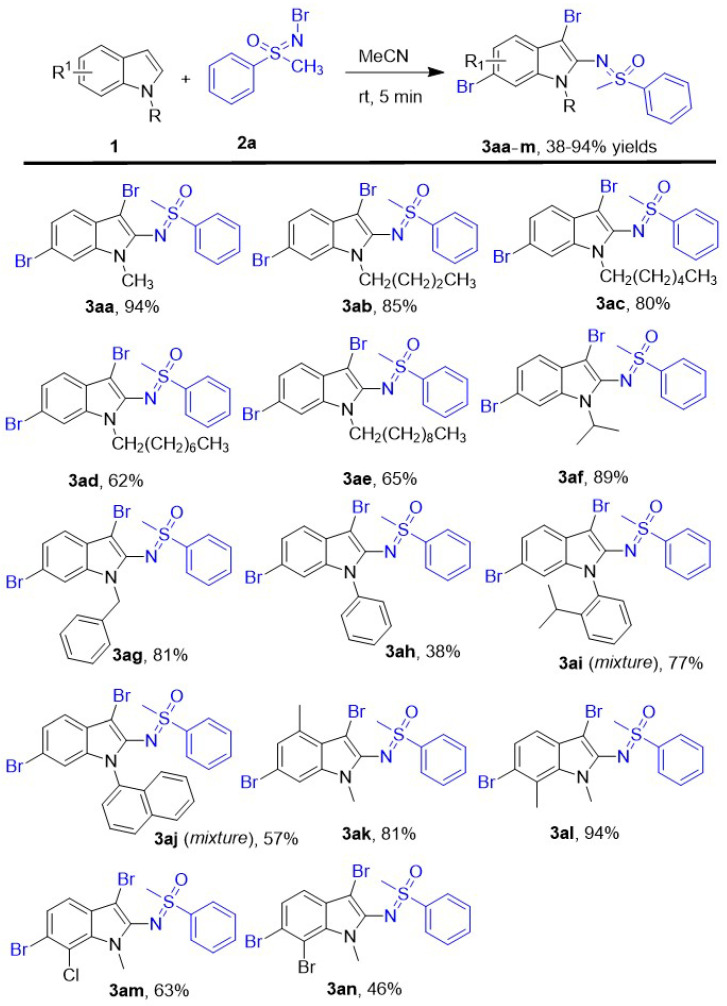
The scope of indoles derivative. Unless otherwise noted, the reactions were performed with Indoles **1** (0.4 mmol), *N*-Br methyl phenyl sulfoximine **2a** (1.2 mmol), 2.0 mL MeCN, stirring 5 min at room temperature. All yields are isolated yields.

The substrate scope of indole derivatives was investigated in this new oxidative dibromo sulfoximination of *N*-R (R = alkyl or aryl) indoles using *N*-Br methyl phenyl sulfoximine as both of brominating and sulfoximinating reagents, and a series of corresponding 3,6-dibrom-2-sulfoximidoyl indoles were afforded in 38–94% yields under the optimal reaction conditions. The details of reaction were shown in Scheme 2. According to the results, the *N*-alkyl indoles including chain alkyl and benzyl groups have been investigated here, and all of them afforded the corresponding 2-sulfoximidoyl-3,6-dibromo indole derivatives **3aa**-**g** in 62–94% yields. The lower yield was given by the indoles which has longer protecting alkyl chain of N atom. So the corresponding product **3aa** was given in the best 94% yield by the *N*-CH_3_ indole, and *N*-octyl indole gave the product **3ad** in worst 62% yield. The steric *N*-isopropyl indole was also performed smoothly in this new protocol to lead to compound **3af** in good 89% yield, and the high yield showed the steric hindrance of *N*-protecting groups was affected this trifunctionalization of indole rarely. Furthermore, the *N*-benzyl indole which has an active methylene group was evaluated in this simple protocol and led to the product **3ag** in good 81% yield. Besides different *N*- alkyl indoles, several *N*-aryl indoles have also been attempted as indole substrates here, and the desired products **3ah**-**j** have been obtained as expectant in 38–77% yields. Among them, the best yield was given by the *N*-(2-isopropylphenyl) indole (**3ai**, 77%) which contained a big 2-isopropylphenyl as protecting group of N atom. Compared to the *N*-(2-isopropylphenyl) indole, the *N*-phenyl indole and *N*-naphthyl indole gave corresponding products **3ah** and **3aj** in decreased yields 38% and 57% yields respectively. Moreover, several substituted *N*-methyl indoles have been evaluated in this new transformation, too. When the 4- or 7- position of *N*-methyl indole was occupied by electron-donating methyl group, the corresponding products **3ak** and **3al** were given in good 81% and excellent 94% yields, respectively. Compared to the *N*-methyl indole, the 7-methyl group of *N*-methyl indole showed no obvious effect on the yield, and the 4-methyl group would make the yield slight decreased to 81%. When the 4-position of *N*-methyl indole was substituted by halogen Cl or Br, the reaction yields of the corresponding products **3am** and **3an** were decreased to 63% and 46%, respectively.

### 2.3. Extending the Scope of N-Br Sulfoximines Substrates

**Scheme 3:**
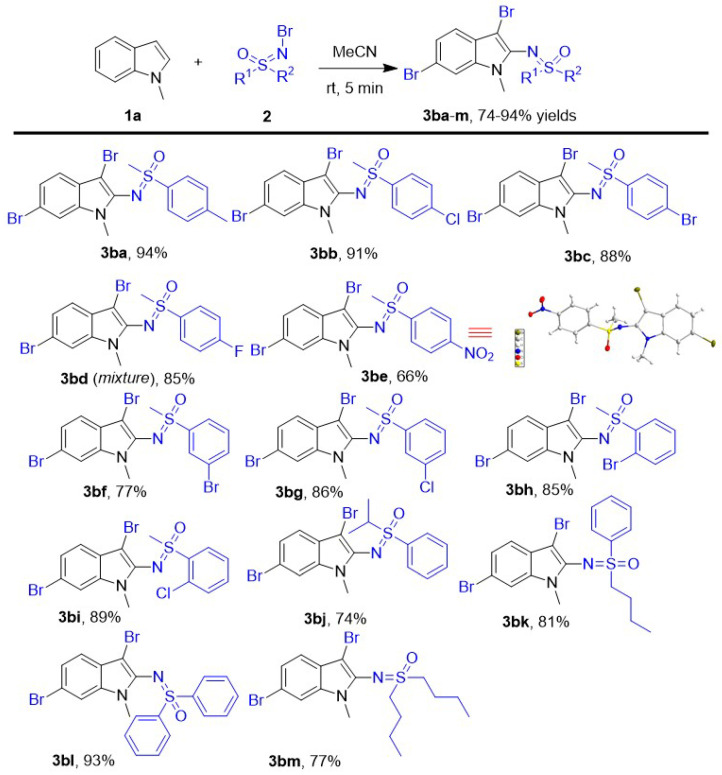
The scope of *N*-Br sulfoximines. Unless otherwise noted, the reactions were performed with Indoles 1a (0.4 mmol) and N-Br sulfoximines 2 (1.2 mmol) in 2.0 mL MeCN with stirring for 5 min at room temperature. All yields are isolated.

Subsequently, various *N*-Br sulfoximines have been implemented into this new tri-functionalization of *N*-methyl indoles as both of brominating and sulfoximinating reagents to evaluate the effect of different R^1^ or R^2^ group of *N*-Br sulfoximines (Scheme 3). First, versatile *N*-Br substituted phenyl methyl sulfoximines have been investigate into the dibromosulfoximination of *N*-methyl indole and all of them led to desired products **3ba–3bi** smoothly in good–excellent yields (66–94%). For different *N*-Br *para*-substituted phenyl methyl sulfoximines, the electron-deficient substituents on the benzene ring led to decreased yields. Among them, the worst yield (**3be**, 66%) was given by the *N*-Br *para*-NO_2_ phenyl methyl sulfoximines, and the highest yield (**3ba**, 94%) was given by the *para*-CH_3_ phenyl methyl sulfoximines. When the *N*-Br *para*-halogenated phenyl methyl sulfoximines were investigated as substrate in this new protocol, all of them performed very well in the reaction, and the corresponding products **3bb**-**d** were given in good–excellent 85–91% yields because of the electron withdrawal of halogen on the benzene ring. Compared to the *N*-Br *para*-bromo/chlorophenyl methyl sulfoximines, similar *N*-Br *meta*-bromo/cholorophenyl methyl sulfoximines were also achieved smoothly in this protocol and afforded corresponding products **3bf** and **3bg** smoothly in slightly lower 77% and 86% yields, respectively. Meanwhile, the 2-sulfoximidoyl-3,6-dibromo indoles **3bh** and **3bi** were obtained in good 85% and 89% yields using *N*-Br *ortho*-Bromo/cholorphenyl methyl sulfoximines as aminohalogenated substrates. The results of all tested *N*-Br halogenated phenyl methyl sulfoximines showed the electronic and steric effect has little influence in the yields. Subsequently, we varied the substituents of *N*-Br phenyl sulfoximines from steric isopropyl chain *n*-butyl to phenyl to evaluate the effect of substituent: the *N*-Br diphenyl sulfoximines led to the corresponding product **3bl** in a similar 91% yield to *N*-Br phenyl methyl sulfoximines, and the steric *N*-Br isopropyl phenyl sulfoximine produced the compound **3bj** in the lowest 74% yield. The *N*-Br *n*-butyl phenyl sulfoximine performed very well here and afforded the product **3bk** in lower 81% yield compared to *N*-Br methyl phenyl sulfoximine. Besides *N*-Br phenyl sulfoximines, *N*-Br di-*n*-butyl sulfoximine also worked in this protocol and led to product **3bm** in 77% yield.

### 2.4. Control Reactions

**Scheme 4:**
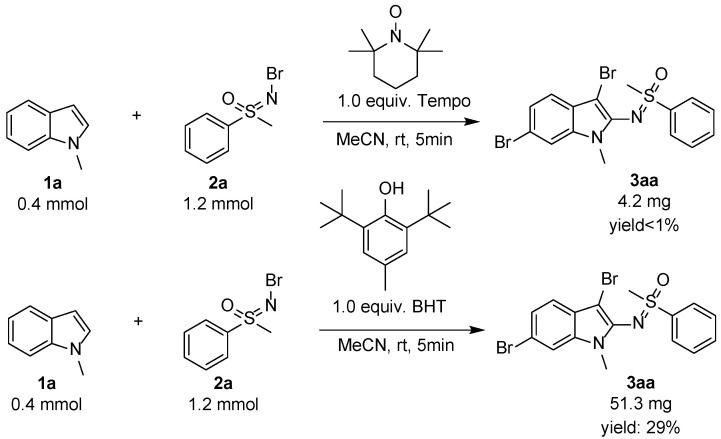
Control reactions and possible mechanism.

Due to the important synthetic significance and potential application of this useful new methodology, the mechanism was studied. The control reactions were assessed, and the results are shown in Scheme 4. Based on similar reported procedures [35,36,38], a radical process was proposed to have occurred, so 1.0 equiv. radical scavengers Tempo or BHT were added to the reaction mixture and led to the corresponding product **3aa** in trace yield or low 29% yield from 94% yield as expected. Most of the starting materials were left in the reaction mixture. Combining the results in the optimization, we found only 2,3-disubstituted indole was given when the amount of *N*-Br sulfoximines was less than 1.5 equivalent, and the 2,3,6-trisubstituted product would be increased gradually with the increased *N*-Br sulfoximines, so the final step must be the radical substitution at the six-position of indoles. The radical 3,6-dibromo-2-sulfoximination of *N*-R (R = alkyl and Ar) indoles was proved in this new protocol.

## 3. Chemistry

All solvents were obtained from commercial sources and used without further purification unless otherwise noted. The *N*-bromosulfoximines were prepared according to literature protocols [44,45]. All N-protected indoles were prepared according to literature protocols [46,47]. Other chemicals were obtained from Energy Chemical and Titan. ^1^H and ^13^C NMR spectra were recorded on a Bruker DRX-400 spectrometer using CDCl_3_ or DMSO-d_6_ as solvent and TMS as an internal standard. Mass spectra (API) were tested on an Agilent 6100 using liquid chromatography–mass spectrometry. Single-crystal X-ray diffraction was conducted in the X-ray and Spectral Center at Huazhong University of Science and Technology, Wuhan, China.

### General Procedure (GP) for the Preparation of Products 3

*N*-bromosulfoximine **2** (1.2 mmol, 3 equiv.) was added to the solution of *N*-protected indole **1** (0.4 mmol) in MeCN (2 mL) in a 15 mL dry pressure tube equipped with a stirring bar. The reaction mixture was stirred at room temperature for another 5 min at room temperature. Then, the organic layer of the reaction mixture was removed under reduced pressure and the residue purified by column chromatography on a neutral alumina column using a mixture of petroleum ether and ethyl acetate as eluent to afford product **3**.

*3,6-Dibromo-2-(methyl phenylsulfoximidoyl)-1-methyl-1H-indole* (***3aa***) According to the general procedure (GP) for the preparation of product 3, the 3,6-dibromo-2-(methyl phenylsulfoximidoyl)-1-methyl-1*H*-indole was prepared as black oil in 94% yield. ^1^H NMR (400 MHz, CDCl_3_) *δ* 8.25–8.20 (m, 2H), 7.73–7.68 (m, 1H), 7.66–7.60 (m, 2H), 7.37 (d, *J* = 1.6 Hz, 1H), 7.26 (s, 1H), 7.22 (dd, *J* = 8.4, 1.6 Hz, 1H), 3.71 (s, 3H), 3.26 (s, 3H). ^13^C NMR (100 MHz, CDCl_3_) *δ* 139.2, 137.7, 135.0, 134.1, 129.7, 128.5, 125.6, 123.2, 119.2, 114.5, 112.1, 80.8, 44.6, 30.2.

*3,6-Dibromo-2-(methyl phenylsulfoximidoyl)-1-butyl-1H-indole (**3ab**)* According to the general procedure (GP) for the preparation of product 3, the 3,6-dibromo-2-(methyl phenylsulfoximidoyl)-1-butyl-1*H*-indole was prepared as black oil in 85% yield. ^1^H NMR (400 MHz, Chloroform-*d*) *δ* 8.25–8.18 (m, 2H), 7.73–7.66 (m, 1H), 7.65–7.58 (m, 2H), 7.37 (d, *J* = 1.7 Hz, 1H), 7.29–7.18 (m, 3H), 4.18 (dddd, *J* = 49.3, 14.5, 8.5, 6.7 Hz, 2H), 3.25 (s, 3H), 1.81–1.64 (m, 2H), 1.36 (dt, *J* = 14.9, 7.4 Hz, 2H), 0.93 (t, *J* = 7.4 Hz, 3H). ^13^C NMR (100 MHz, Chloroform-*d*) *δ* 139.6, 137.5, 134.2, 134.0, 129.6, 128.5, 125.8, 123.0, 119.2, 114.4, 112.3, 80.4, 44.7, 43.4, 31.9, 20.3, 13.9.

*3,6-Dibromo-2-(methyl phenylsulfoximidoyl)-1-hexyl-1H-indole (**3ac**)* According to the general procedure (GP) for the preparation of product 3, the 3,6-dibromo-2-(methyl phenylsulfoximidoyl)-1-hexyl-1*H*-indole was prepared as black oil in 80% yield. ^1^H NMR (400 MHz, DMSO-*d*_6_) *δ* 8.14–8.08 (m, 2H), 7.78–7.72 (m, 1H), 7.71–7.65 (m, 2H), 7.61 (d, *J* = 1.6 Hz, 1H), 7.17–7.10 (m, 2H), 4.19 (pt, *J* = 7.9, 4.0 Hz, 2H), 3.51 (s, 3H), 1.63 (qd, *J* = 10.3, 4.6 Hz, 2H), 1.31–1.19 (m, 6H), 0.87–0.78 (m, 3H). ^13^C NMR (100 MHz, CDCl_3_) *δ* 139.6, 138.3, 133.8, 133.5, 129.5, 127.7, 125.3, 122.4, 118.5, 113.1, 112.1, 77.8, 44.6, 42.5, 30.9, 29.1, 25.8, 22.1, 13.9. HR-MS(ESI), *m*/*z* (%): Calcd for C_21_H_25_Br_2_N_2_OS^+^ ([M+H]^+^): 511.0049, Found: 511.0052.

*3,6-Dibromo-2-(methyl phenylsulfoximidoyl)-1-octyl-1H-indole (**3ad**)* According to the general procedure (GP) for the preparation of product 3, the 3,6-dibromo-2-(methyl phenylsulfoximidoyl)-1-octyl-1*H*-indole was prepared as black oil in 62% yield. ^1^H NMR (400 MHz, CDCl_3_) *δ* 8.26–8.18 (m, 2H), 7.74–7.66 (m, 1H), 7.62 (dd, *J* = 8.3, 6.7 Hz, 2H), 7.36 (d, *J* = 1.6 Hz, 1H), 7.27 (d, *J* = 7.4 Hz, 1H), 7.20 (dd, *J* = 8.4, 1.6 Hz, 1H), 4.28–4.05 (m, 2H), 3.25 (s, 3H), 1.75 (h, *J* = 6.8 Hz, 2H), 1.36–1.20 (m, 10H), 0.86 (t, *J* = 6.7 Hz, 3H). ^13^C NMR (100 MHz, CDCl_3_) *δ* 139.4, 137.4, 134.1, 134.0, 129.6, 128.5, 125.7, 123.0, 119.2, 114.3, 112.2, 80.4, 44.6, 43.6, 31.9, 29.8, 29.4, 29.3, 27.0, 22.8, 14.3. Calcd for C_23_H_29_Br_2_N_2_OS^+^ (M): 539.0362, Found: 539.0355.

*3,6-Dibromo-2-(methyl phenylsulfoximidoyl)-1-decyl-1H-indole (**3ae**)* According to the general procedure (GP) for the preparation of product 3, the 3,6-dibromo-2-(methyl phenylsulfoximidoyl)-1-decyl-1*H*-indole was prepared as black oil in 65% yield. ^1^H NMR (400 MHz, CDCl_3_) *δ* 8.24–8.19 (m, 2H), 7.71–7.66 (m, 1H), 7.61 (dd, *J* = 8.4, 6.8 Hz, 2H), 7.36 (d, *J* = 1.7 Hz, 1H), 7.27 (s, 1H), 7.20 (dd, *J* = 8.4, 1.6 Hz, 1H), 4.28–4.04 (m, 2H), 3.24 (s, 3H), 1.75 (h, *J* = 7.4, 7.0 Hz, 2H), 1.37–1.19 (m, 14H), 0.87 (t, *J* = 6.8 Hz, 3H). ^13^C NMR (100 MHz, CDCl_3_) *δ* 139.6, 137.5, 134.2, 134.0, 129.6, 128.4, 125.8, 123.0, 119.2, 114.3, 112.2, 80.4, 44.7, 43.6, 32.0, 29.8, 29.7 (2C), 29.4 (2C), 27.0, 22.8, 14.2. HR-MS(ESI), *m*/*z* (%): Calcd for C_25_H_33_Br_2_N_2_OS^+^ ([M+H]^+^): 567.0675, Found: 567.0669.

*3,6-Dibromo-2-(methyl phenylsulfoximidoyl)-1-isopropyl-1H-indole (**3af**)* According to the general procedure (GP) for the preparation of product 3, the 3,6-dibromo-2-(methyl phenylsulfoximidoyl)-1-isopropyl-1*H*-indole was prepared as green oil in 89% yield. ^1^H NMR (400 MHz, CDCl_3_) *δ* 8.29–8.20 (m, 2H), 7.75–7.67 (m, 1H), 7.66–7.58 (m, 3H), 7.32–7.17 (m, 3H), 5.10 (p, *J* = 7.1 Hz, 1H), 3.23 (s, 3H), 1.64 (d, *J* = 7.0 Hz, 3H), 1.53 (d, *J* = 7.1 Hz, 3H). ^13^C NMR (100 MHz, CDCl_3_) *δ* 139.2, 136.8, 134.1, 132.6, 129.7, 128.5, 126.3, 122.8, 119.5, 114.2, 114.0, 81.2, 47.2, 44.1, 21.7, 21.2.

*3,6-Dibromo-2-(methyl phenylsulfoximidoyl)-1-benzyl-1H-indole (**3ag**)* According to the general procedure (GP) for the preparation of product 3, the 3,6-dibromo-2-(methyl phenylsulfoximidoyl)-1-benzyl-1*H*-indole was prepared as white solid in 81% yield. M.p.: 162.5–163.4 °C. ^1^H NMR (400 MHz, DMSO-*d*_6_) *δ* 7.97–7.90 (m, 2H), 7.74–7.67 (m, 1H), 7.61 (dd, *J* = 8.4, 7.1 Hz, 2H), 7.55 (d, *J* = 1.5 Hz, 1H), 7.31 (dd, *J* = 8.1, 6.6 Hz, 2H), 7.27–7.21 (m, 1H), 7.18–7.09 (m, 4H), 5.58–5.40 (m, 2H), 3.50 (s, 3H). ^13^C NMR (100 MHz, DMSO-*d*_6_) *δ* 139.2, 138.9, 138.1, 133.8, 133.6, 129.5, 128.6, 127.7, 127.2, 126.9, 125.6, 122.8, 118.6, 113.2, 112.4, 77.7, 45.6, 44.7. HR-MS(ESI), *m*/*z* (%): Calcd for C_22_H_19_Br_2_N_2_OS^+^ ([M+H]^+^): 516.9579, Found: 516.9587.

*3,6-Dibromo-2-(methyl phenylsulfoximidoyl)-1-phenyl-1H-indole (**3ah**)* According to the general procedure (GP) for the preparation of product 3, the 3,6-dibromo-2-(methyl phenylsulfoximidoyl)-1-phenyl-1*H*-indole was prepared as yellow oil in 38% yield. ^1^H NMR (400 MHz, CDCl_3_) *δ* 7.78–7.73 (m, 2H), 7.60–7.49 (m, 3H), 7.49–7.38 (m, 5H), 7.31 (d, *J* = 8.3 Hz, 1H), 7.26–7.24 (m, 1H), 7.24–7.21 (m, 1H), 3.07 (s, 3H). ^13^C NMR (100 MHz, CDCl_3_) *δ* 139.6, 137.9, 136.8, 135.0, 133.7, 129.5, 129.4, 129.3, 128.5, 128.1 (2C), 126.1, 123.9, 119.4, 115.0, 112.8, 82.3, 44.9. HR-MS(ESI), *m*/*z* (%): Calcd for C_21_H_17_Br_2_N_2_OS^+^ ([M+H]^+^): 502.9423, Found: 502.9429.

*3,6-Dibromo-2-(methyl phenylsulfoximidoyl)-1-(2-isopropylphenyl)-1H-indole (**3ai**)* According to the general procedure (GP) for the preparation of product 3, the 3,6-dibromo-2-(methyl phenylsulfoximidoyl)-1-(2-isopropylphenyl)-1*H*-indole was prepared as black oil in 77% yield. ^1^H NMR (400 MHz, CDCl_3_) *δ* 7.79–7.71 (m, 1H), 7.58–7.46 (m, 4H), 7.45–7.20 (m, 5H), 7.16–7.09 (m, 1H), 6.94 (dd, *J* = 16.6, 1.7 Hz, 1H), 3.10 (d, *J* = 33.4 Hz, 3H), 1.15 (dd, *J* = 13.2, 6.8 Hz, 3H), 0.99 (dd, *J* = 6.9, 4.2 Hz, 3H). ^13^C NMR (100 MHz, DMSO-*d*_6_) *δ* 149.2, 148.4, 140.2 (2C), 139.1, 138.9, 135.8 (2C), 134.6, 134.2, 133.6, 133.5, 130.2, 129.6 (2C), 129.4, 129.3, 128.3, 128.0, 127.2, 126.8, 126.6, 126.5, 126.3, 126.1, 123.7, 123.6, 119.3, 119.1, 114.8, 114.7, 112.7 (2C), 80.4, 80.1, 45.7, 44.8, 28.3, 28.1, 24.8, 24.6, 23.4, 23.0. HR-MS(ESI), *m*/*z* (%): Calcd for C_24_H_23_Br_2_N_2_OS^+^ ([M+H]^+^): 544.9892, Found: 544.9900.

*3,6-Dibromo-2-(methyl phenylsulfoximidoyl)-1-(naphthalen-1-yl)-1H-indole (**3aj**)* According to the general procedure (GP) for the preparation of product 3, the 3,6-dibromo-2-(methyl phenylsulfoximidoyl)-1-(naphthalen-1-yl)-1*H*-indole was prepared as black oil in 57% yield. ^1^H NMR (400 MHz, CDCl_3_) *δ* 8.04–7.93 (m, 2H), 7.60 (dd, *J* = 8.3, 7.2 Hz, 1H), 7.55–7.32 (m, 7H), 7.30–7.14 (m, 4H), 6.87 (dd, *J* = 3.2, 1.7 Hz, 1H), 2.98 (d, *J* = 12.5 Hz, 3H). ^13^C NMR (100 MHz, DMSO-*d*_6_) *δ* 139.8 (2C), 139.4, 138.9, 136.1, 136.0, 134.5 (2C), 133.5, 133.4 (2C), 131.6, 131.5, 129.4 (2C), 129.2, 129.1, 128.4, 128.3, 128.1, 128.0, 127.9, 127.4, 127.3, 126.8, 126.7, 126.3 (2C), 125.7, 125.4, 123.9, 123.8, 123.3, 119.4 (2C), 115.0 (2C), 113.1, 113.0, 82.1, 81.1, 45.1, 44.9.

*3,6-Dibromo-2-(methyl phenylsulfoximidoyl)-1,4-dimethyl-1H-indole (**3ak**)* According to the general procedure (GP) for the preparation of product 3, the 3,6-dibromo-2-(methyl phenylsulfoximidoyl)-1,4-dimethyl-1*H*-indole was prepared as yellow oil in 80% yield. ^1^H NMR (400 MHz, CDCl_3_) *δ* 8.30–8.23 (m, 2H), 7.74–7.67 (m, 1H), 7.67–7.60 (m, 2H), 7.23 (d, *J* = 1.7 Hz, 1H), 6.96 (dd, *J* = 1.9, 1.0 Hz, 1H), 3.68 (s, 3H), 3.23 (s, 3H), 2.75 (s, 3H). ^13^C NMR (100 MHz, CDCl_3_) *δ* 139.2, 137.3, 135.2, 134.1, 131.2, 129.6, 128.5, 124.7, 122.8, 114.1, 110.3, 81.0, 44.1, 30.4, 19.3.

*3,6-Dibromo-2-(methyl phenylsulfoximidoyl)-1,7-dimethyl-1H-indole (**3al**)* According to the general procedure (GP) for the preparation of product 3, the 3,6-dibromo-2-(methyl phenylsulfoximidoyl)-1,7-dimethyl-1*H*-indole was prepared as black solid in 94% yield. M.p.: 122.6–124.2 °C. ^1^H NMR (400 MHz, CDCl_3_) *δ* 8.27–8.18 (m, 2H), 7.73–7.65 (m, 1H), 7.62 (dd, *J* = 8.3, 6.7 Hz, 2H), 7.29 (d, *J* = 8.4 Hz, 1H), 7.10 (d, *J* = 8.4 Hz, 1H), 3.96 (s, 3H), 3.23 (s, 3H), 2.81 (s, 3H). ^13^C NMR (100 MHz, CDCl_3_) *δ* 139.3, 138.3, 134.2, 134.0, 129.6, 128.5, 126.8, 124.8, 120.7, 119.4, 116.9, 81.6, 44.3, 34.0, 19.0. HR-MS(ESI), *m*/*z* (%): Calcd for C_17_H_17_Br_2_N_2_OS^+^ ([M+H]^+^): 454.9423, Found:454.9426.

*3,6-Dibromo-2-(methyl phenylsulfoximidoyl)-7-chloro-1-methyl-1H-indole (**3am**)* According to the general procedure (GP) for the preparation of product 3, the 3,6-dibromo-2-(methyl phenylsulfoximidoyl)-7-chloro-1-methyl-1*H*-indole was prepared as yellow oil in 63% yield. ^1^H NMR (400 MHz, CDCl_3_) *δ* 8.24–8.18 (m, 2H), 7.73–7.67 (m, 1H), 7.63 (ddt, *J* = 8.3, 6.8, 1.4 Hz, 2H), 7.31 (d, *J* = 8.4 Hz, 1H), 7.15 (d, *J* = 8.4 Hz, 1H), 4.08 (s, 3H), 3.26 (s, 3H). ^13^C NMR (100 MHz, CDCl_3_) *δ* 139.2, 139.1, 134.2, 131.0, 129.7, 128.4, 128.3, 124.9, 117.5, 116.7, 116.2, 81.3, 44.7, 33.4.

*3,6,7-Tribromo-2-(methyl phenylsulfoximidoyl)-1-methyl-1H-indole (**3an**)* According to the general procedure (GP) for the preparation of product 3, the 3,6,7-tribromo-2-(methyl phenylsulfoximidoyl)-1-methyl-1*H*-indole was prepared as black oil in 46% yield. ^1^H NMR (400 MHz, DMSO-*d*_6_) *δ* 8.13–8.08 (m, 2H), 7.78–7.72 (m, 1H), 7.71–7.65 (m, 2H), 7.36 (d, *J* = 8.4 Hz, 1H), 7.12 (d, *J* = 8.3 Hz, 1H), 4.02 (s, 3H), 3.56 (s, 3H). ^13^C NMR (100 MHz, DMSO-*d*_6_) *δ* 140.3, 139.2, 133.9, 131.5, 129.5, 127.9, 127.7, 124.7, 117.8, 117.6, 105.3, 79.0, 44.4, 33.0.

*3,6-Dibromo-2-(methyl p-tolylsulfoximidoyl)-1-methyl-1H-indole (**3ba**)* According to the general procedure (GP) for the preparation of product 3, the 3,6-dibromo-2-(methyl *p*-tolylsulfoximidoyl)-1-methyl-1*H*-indole was prepared as purple oil in 84% yield. ^1^H NMR (400 MHz, CDCl_3_) *δ* 8.13–8.05 (m, 2H), 7.42 (d, *J* = 8.0 Hz, 2H), 7.36 (d, *J* = 1.7 Hz, 1H), 7.28 (s, 1H), 7.22 (dd, *J* = 8.4, 1.6 Hz, 1H), 3.71 (s, 3H), 3.24 (s, 3H), 2.48 (s, 3H). ^13^C NMR (101 MHz, CDCl_3_) *δ* 145.2, 137.9, 136.1, 135.0, 130.3, 128.5, 125.6, 123.1, 119.2, 114.5, 112.1, 80.8, 44.7, 30.2, 21.8. HR-MS(ESI), *m*/*z* (%): Calcd for C_17_H_17_Br_2_N_2_OS^+^ ([M+H]^+^): 454.9423, Found: 454.9438.

*3,6-Dibromo-2-(methyl 4-chlorophenylsulfoximidoyl)-1-methyl-1H-indole (**3bb**)* According to the general procedure (GP) for the preparation of product 3, the 3,6-dibromo-2-(methyl 4-chlorophenylsulfoximidoyl)-1-methyl-1*H*-indole was prepared as purple oil in 91% yield. ^1^H NMR (400 MHz, CDCl_3_) *δ* 8.18–8.12 (m, 2H), 7.62–7.56 (m, 2H), 7.36 (d, *J* = 1.6 Hz, 1H), 7.29–7.19 (m, 2H), 3.69 (s, 3H), 3.25 (s, 3H). ^13^C NMR (100 MHz, CDCl_3_) *δ* 141.0, 137.7, 137.3, 135.0, 130.0, 129.9, 125.5, 123.3, 119.2, 114.7, 112.2, 80.7, 44.7, 30.2. HR-MS(ESI), *m*/*z* (%): Calcd for C_16_H_14_Br_2_ClN_2_OS^+^ ([M+H]^+^): 474.8877, Found: 474.8897.

*3,6-Dibromo-2-(methyl 4-bromophenylsulfoximidoyl)-1-methyl-1H-indole (**3bc**)* According to the general procedure (GP) for the preparation of product 3, the 3,6-dibromo-2-(methyl 4-bromophenylsulfoximidoyl)-1-methyl-1*H*-indole was prepared as purple oil in 88% yield. ^1^H NMR (400 MHz, DMSO-*d*_6_) *δ* 8.05–8.00 (m, 2H), 7.91–7.86 (m, 2H), 7.60 (d, *J* = 1.6 Hz, 1H), 7.16 (dd, *J* = 8.3, 1.6 Hz, 1H), 7.12 (d, *J* = 8.4 Hz, 1H), 3.68 (s, 3H), 3.57 (s, 3H). ^13^C NMR (100 MHz, DMSO-*d*_6_) *δ* 138.8, 138.4, 134.2, 132.5, 129.9, 127.9, 125.1, 122.5, 118.4, 113.2, 112.2, 78.0, 44.3, 29.7.

*3,6-Dibromo-2-(methyl 4-fluorophenylsulfoximidoyl)-1-methyl-1H-indole (mixture **3bd**)* According to the general procedure (GP) for the preparation of product 3, the 3,6-dibromo-2-(methyl 4-fluorophenylsulfoximidoyl)-1-methyl-1*H*-indole and 3,6-dibromo-2-(methyl 4-fluorophenylsulfoximidoyl)-1-methyl-1*H*-indole were prepared as orange solid in about 85% yield (the ratio of trisubstituted product to disubstituted product was about 3.5: 1). ^1^H NMR (400 MHz, CDCl_3_, main tri-substituted product) *δ* 8.24 (dd, *J* = 9.0, 5.0 Hz, 2H), 7.37 (d, *J* = 1.6 Hz, 1H),7.32–7.26 (m, 4H), 7.24–7.21 (m, 1H), 3.71 (3H), 3.26 (3H); ^13^C NMR (100 MHz, CDCl_3_) (main tri-substituted product) *δ* 166.2 (d, *J* = 255.4), 137.5, 135.0, 131.5 (d, *J* = 9.7 Hz), 125.5, 123.2, 119.2, 117.9, 117.0 (d, *J* = 22.6 Hz), 114.6, 112.1, 80.8, 44.7, 30.2.

*3,6-Dibromo-2-(methyl 4-nitrophenylsulfoximidoyl)-1-methyl-1H-indole (**3be**)* According to the general procedure (GP) for the preparation of product 3, the 3,6-dibromo-2-(methyl 4-nitrophenylsulfoximidoyl)-1-methyl-1*H*-indole was prepared as orange solid in 66% yield. M.p.: 157.3–158.1 °C. ^1^H NMR (400 MHz, DMSO-*d*_6_) *δ* 8.46 (d, *J* = 8.8 Hz, 2H), 8.36 (d, *J* = 8.7 Hz, 2H), 7.60 (d, *J* = 1.7 Hz, 1H), 7.19–7.07 (m, 2H), 3.69 (d, *J* = 7.3 Hz, 6H). ^13^C NMR (100 MHz, DMSO-*d*_6_) *δ* 150.4, 145.5, 137.9, 134.2, 129.5, 125.1, 124.6, 122.6, 118.5, 113.3, 112.3, 78.1, 44.0, 29.7.

*3,6-Dibromo-2-(methyl 3-bromophenylsulfoximidoyl)-1-methyl-1H-indole (**3bf**)* According to the general procedure (GP) for the preparation of product 3, the 3,6-dibromo-2-(methyl 3-bromophenylsulfoximidoyl)-1-methyl-1*H*-indole was prepared as black oil in 77% yield. ^1^H NMR (400 MHz, CDCl_3_) δ 8.37 (t, *J* = 1.8 Hz, 1H), 8.14 (ddd, *J* = 8.0, 1.9, 1.0 Hz, 1H), 7.81 (ddd, *J* = 8.0, 1.9, 1.0 Hz, 1H), 7.49 (t, *J* = 7.9 Hz, 1H), 7.36 (dd, *J* = 1.6, 0.5 Hz, 1H), 7.28 (d, *J* = 0.5 Hz, 1H), 7.22 (dd, *J* = 8.4, 1.6 Hz, 1H), 3.70 (s, 3H), 3.28 (s, 3H). ^13^C NMR (100 MHz, CDCl_3_) *δ* 141.1, 137.2, 137.1, 135.0, 131.4, 131.1, 127.1, 125.5, 123.5, 123.3, 119.3, 114.7, 112.2, 80.8, 44.8, 30.2.

*3,6-Dibromo-2-(methyl 3-chlorophenylsulfoximidoyl)-1-methyl-1H-indole (**3bg**)* According to the general procedure (GP) for the preparation of product 3, the 3,6-dibromo-2-(methyl 3-chlorophenylsulfoximidoyl)-1-methyl-1*H*-indole was prepared as black oil in 86% yield. ^1^H NMR (400 MHz, CDCl_3_) *δ* 8.21 (t, *J* = 1.9 Hz, 1H), 8.09 (ddd, *J* = 7.9, 1.8, 1.1 Hz, 1H), 7.65 (ddd, *J* = 8.1, 2.0, 1.1 Hz, 1H), 7.55 (t, *J* = 8.0 Hz, 1H), 7.36 (dd, *J* = 1.6, 0.5 Hz, 1H), 7.27 (d, *J* = 0.5 Hz, 1H), 7.21 (dd, *J* = 8.4, 1.6 Hz, 1H), 3.69 (s, 3H), 3.28 (s, 3H). ^13^C NMR (100 MHz, CDCl_3_) *δ* 141.1, 137.1, 135.8, 135.0, 134.3, 130.9, 128.6, 126.6, 125.5, 123.2, 119.3, 114.7, 112.1, 80.7, 44.7, 30.2.

*3,6-Dibromo-2-(methyl 2-bromophenylsulfoximidoyl)-1-methyl-1H-indole (**3bh**)* According to the general procedure (GP) for the preparation of product 3, the 3,6-dibromo-2-(methyl 2-bromophenylsulfoximidoyl)-1-methyl-1*H*-indole was prepared as black oil in 85% yield. ^1^H NMR (400 MHz, CDCl_3_) *δ* 8.47 (dd, *J* = 8.0, 1.7 Hz, 1H), 7.75 (dd, *J* = 7.9, 1.4 Hz, 1H), 7.55 (td, *J* = 7.7, 1.3 Hz, 1H), 7.48 (td, *J* = 7.6, 1.8 Hz, 1H), 7.33–7.31 (m, 1H), 7.22–7.16 (m, 2H), 3.66 (s, 3H), 3.57 (s, 3H). ^13^C NMR (100 MHz, CDCl_3_) *δ* 139.3, 137.4, 135.9, 134.9, 134.7, 132.8, 128.4, 125.9, 123.0, 120.6, 119.2, 114.3, 111.8, 78.3, 43.7, 29.9.

*3,6-Dibromo-2-(methyl 2-chlorophenylsulfoximidoyl)-1-methyl-1H-indole (**3bi**)* According to the general procedure (GP) for the preparation of product 3, the 3,6-dibromo-2-(methyl 2-chlorophenylsulfoximidoyl)-1-methyl-1*H*-indole was prepared as black oil in 89% yield. ^1^H NMR (400 MHz, DMSO-*d*_6_) *δ* 8.28 (dd, *J* = 7.9, 1.6 Hz, 1H), 7.75–7.60 (m, 3H), 7.58 (d, *J* = 1.7 Hz, 1H), 7.14 (dd, *J* = 8.3, 1.7 Hz, 1H), 7.06 (d, *J* = 8.3 Hz, 1H), 3.74 (s, 3H), 3.66 (s, 3H). ^13^C NMR (100 MHz, DMSO-*d*_6_) *δ* 138.2, 137.3, 135.4, 134.0, 132.1, 132.0, 131.0, 128.2, 125.2, 122.5, 118.4, 113.1, 112.1, 76.5, 43.9, 29.5. HR-MS(ESI), *m*/*z* (%): Calcd for C_16_H_14_Br_2_ClN_2_OS^+^: 474.8877, Found: 474.8863.

*3,6-Dibromo-2-(isopropyl phenylsulfoximidoyl)-1-methyl-1H-indole (**3bj**)* According to the general procedure (GP) for the preparation of product 3, the 3,6-dibromo-2-(isopropyl phenylsulfoximidoyl)-1-methyl-1*H*-indole was prepared as green oil in 74% yield ^1^H NMR (400 MHz, CDCl_3_) *δ* 8.01–7.95 (m, 2H), 7.64–7.59 (m, 1H), 7.56–7.50 (m, 2H), 7.29–7.27 (m, 1H), 7.15 (dd, *J* = 1.8, 1.1 Hz, 2H), 3.69 (s, 3H), 3.58 (p, *J* = 6.8 Hz, 1H), 1.45 (d, *J* = 6.7 Hz, 3H), 1.31 (d, *J* = 6.8 Hz, 3H). ^13^C NMR (100 MHz, CDCl_3_) *δ* 138.5, 136.1, 134.7, 133.8, 129.8, 129.4, 126.0, 122.9, 118.8, 113.8, 111.7, 78.7, 58.2, 30.2, 16.8, 16.0. HR-MS(ESI), *m*/*z* (%): Calcd for C_18_H_19_Br_2_N_2_OS^+^ ([M+H]^+^): 468.9579, Found: 468.9588.

*3,6-Dibromo-2-(butyl phenylsulfoximidoyl)-1-methyl-1H-indole (**3bk**)* According to the general procedure (GP) for the preparation of product 3, the 3,6-dibromo-2-(butyl phenylsulfoximidoyl)-1-methyl-1*H*-indole was prepared as black oil in 81% yield. ^1^H NMR (400 MHz, CDCl_3_) *δ* 8.15–8.08 (m, 2H), 7.69–7.63 (m, 1H), 7.58 (dd, *J* = 8.4, 6.8 Hz, 2H), 7.33 (d, *J* = 1.6 Hz, 1H), 7.24 (d, *J* = 3.4 Hz, 1H), 7.18 (dd, *J* = 8.4, 1.6 Hz, 1H), 3.70 (s, 3H), 3.39 (dddd, *J* = 54.7, 14.0, 11.2, 5.1 Hz, 2H), 1.76–1.53 (m, 2H), 1.38–1.27 (m, 2H), 0.84 (t, *J* = 7.3 Hz, 3H). ^13^C NMR (100 MHz, CDCl_3_) *δ* 138.0, 137.7, 134.8, 133.9, 129.5, 129.0, 125.7, 123.0, 119.0, 114.2, 111.9, 80.2, 56.7, 30.2, 25.3, 21.5, 13.6.

*3,6-Dibromo-2-(diphenylsulfoximidoyl)-1-methyl-1H-indole (**3bl**)* According to the general procedure (GP) for the preparation of product 3, the 3,6-dibromo-2-(diphenylsulfoximidoyl)-1-methyl-1*H*-indole was prepared as black oil in 93% yield. ^1^H NMR (400 MHz, CDCl_3_) *δ* 8.13–8.09 (m, 4H), 7.58–7.46 (m, 6H), 7.30–7.27 (m, 1H), 7.19–7.12 (m, 2H), 3.75 (s, 3H). ^13^C NMR (100 MHz, CDCl_3_) *δ* 140.3, 137.8, 134.9, 133.4, 129.5, 128.5, 125.9, 123.0, 119.1, 114.2, 111.9, 80.5, 30.6. HR-MS(ESI), *m*/*z* (%): Calcd for C_21_H_17_Br_2_N_2_OS^+^ ([M+H]^+^): 502.9423, Found: 502.9429.

*3,6-Dibromo-2-(dibutylsulfoximidoyl)-1-methyl-1H-indole (**3bm**)* According to the general procedure (GP) for the preparation of product 3, the mixture of 3,6-dibromo-2-(dibutylsulfoximidoyl)-1-methyl-1*H*-indole and 2,6-dibromo-3-(dibutylsulfoximidoyl)-1-methyl-1*H*-indole was prepared as black oil in 77% yield. ^1^H NMR (400 MHz, CDCl_3_) *δ* 7.34 (d, *J* = 1.6 Hz, 1H), 7.27 (s, 1H), 7.20 (dd, *J* = 8.4, 1.6 Hz, 1H), 3.66 (d, *J* = 18.4 Hz, 3H), 3.33–3.14 (m, 4H), 1.99–1.75 (m, 4H), 1.47 (h, *J* = 7.4 Hz, 4H), 0.95 (t, *J* = 7.4 Hz, 6H). ^13^C NMR (for main product) (100 MHz, CDCl_3_) *δ* 138.1, 134.8, 125.5, 123.0, 119.0, 114.3, 112.0, 80.8, 52.8, 30.07, 25.1, 21.9, 13.7.

## 4. Conclusions

In conclusion, a green 3,6-dibromo-2-sulfoximination of *N*-R (R = alkyl, benzyl and aryl) indoles using *N*-Br sulfoximines as both brominating and sulfoximinating reagents was developed, and a total of 27 products were prepared in 38–94% yields. This protocol introduces two bromo and one sulfoximidoyl group on indole moiety with absolute atom and step economy by stirring the reaction mixture for 5 min in CH_3_CN at room temperature. This method is compatible with a wide range of substrates and affords most of the products in >60% yields. It also adheres to the principles of green chemistry. A range of potential bioactive sulfoximidoyl indoles with two good-leaving-group Br was prepared via this novel trifunctionalization of indoles in good to excellent yields. According to the control reactions, this is a radical bromosulfoximidoyl process involving Br radical and sulfoximidoyl radical generated by *N*-Br sulfoximine in the presence of *N*-R (R = alkyl, benzyl or aryl) indoles. This new green synthesis of *N*-methyl-3,6-dibromo-2-sulfoximidoyl indoles will facilitate the understanding of transition-metal-free trifunctionalization of indoles and the development of research based on sulfoximidoyl indoles. Additionally, the successful application of *N*-X sulfoximines as both sulfoximinating and halogenating reagents would further promote the development of sulfoximination and halogenation. Organic syntheses or biochemistry based on indoles or sulfoximines will be greatly developed. In the near future, we will test the bioactivity of this new sulfoximidoyl indoles as pesticide, silkworm medicine, and medicine. We will also continue to expand the library of sulfoximidoyl indoles through the functionalization of the 3- or 6-position C-Br bond.

## 5. Patents

One Chinese invention patent (CN113121403B) resulting from this work is reported in this manuscript [48].

## Figures and Tables

**Table 1:** Exploring experimental conditions.

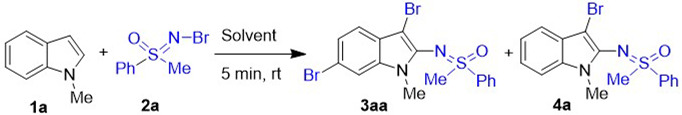				
**Entry**	**Equiv. of 2a**	**Solvent**	**Yield of 3aa**	**Yield of 4a**
1	1	MeCN	trace	22%
2	2	MeCN	trace	62%
**3**	**3**	**MeCN**	**94%**	**trace**
4	3	THF	80%	trace
5	3	Toluene	trace	trace
6	3	1,4-Dioxane	72%	trace
7	3	EtOH	28%	trace
8	3	DCE	64%	trace

Normal conditions: The *N*-Br methyl phenyl sulfoximine **2a** was added into the solution of 0.4 mmol *N*-Me indole in 2 mL solvent, stirring for 5 min at room temperature.

## Data Availability

The data presented in this study are available in the Supplementary Materials.

## Supplementary Materials

The following supporting information can be downloaded at: https://www.mdpi.com/article/10.3390/molecules28083380/s1, including general procedure (GP) for the preparation of N-bromosulfoximines, general procedure (GP) for the preparation of N-aryl indole, general procedure (GP) for the preparation of N-alkyl indole, general procedure (GP) for the preparation of various N-methyl indole and additional optimization (Table S1. Characterization data of substrates, X-ray crystallography of product **3be**, and ^1^H and ^13^C NMR spectra of substrates and products **3**); Table S1: The additional optimization of bromosulfoximidation of N-Me indole; Table S2: Crystal data and structure refinement; References [49,50,51] are cited in the supplementary materials.

## Sample Availability

Samples of the compounds are not available from the authors.
