# Supplementary material for: Green One-Pot Syntheses of 2-Sulfoximidoyl-3,6-dibromo Indoles Using N-Br Sulfoximines as Both Brominating and Sulfoximinating Reagents
Source: Molecules. 2023 Apr 11;28(8):3380. doi: 10.3390/molecules28083380 (PMC10146707; doi:10.3390/molecules28083380)
Supplement: Supplementary file 1 [file molecules-28-03380-s001.zip › molecules-2298227-supplementary.pdf]

Green One-Pot Syntheses of 2-Sulfoximidoyl-3,6-Dibromo Indoles using *N*-Br Sulfoximines as Both Brominating Reagents and Sulfoximinating Reagents

Xiao Yun Chen[1,] *, Yaonan Tang[1], Xinran Xiang[1], Yisong Tang[1], Mingyang Huang[1], Shaojun Zheng[1,] * and Cuifeng Yang[2,3]

1 School of Environmental and Chemical Engineering, Jiangsu University of Science and Technology, Zhenjiang 212003, Jiangsu province, China;

2 Modern Chemistry Research Institute of Xi'an, Xi'an 710065, China;

3 State Key Laboratory of Fluorine & Nitrogen Chemicals, Xi'an 710065, China

**E-mail: chenxy@just.edu.cn; sz281cam@just.edu.cn*

Supporting Information

Table of Contents

**1.**	**General information**	**S2**
2.1	**General procedure (GP) for the preparation of *N*-bromosulfoximines**	**S2**
2.2	**General procedure (GP) for the preparation of *N*-aryl indole**	**S2**
2.3	**General procedure (GP) for the preparation of *N*-alkyl indole**	**S2**
2.4	**General procedure (GP) for the preparation of various *N*-methyl indole**	**S3**
2.6	**The additional optimization of bromosulfoximination of *N*-Me indole**	
3.	**Characterization data**	**S4**
**4.**	**X-ray crystallography**	**S13**
**5.**	**Reference**	**S13**
**6.**	**NMR spectra for all compounds**	**S14**

1 General Information

All solvents were obtained from commercial sources and used without further purification unless otherwise noted. The *N*-Br sulfoximines were prepared according to literature protocols.[1] All *N*-protected indole were prepared according to literature protocols.[2] Other chemicals were obtained from Energy Chemical and Titan. ^{1}H and ^{13}C NMR spectra were recorded on a Bruker DRX-400 spectrometer using $CDCl_3$ or DMSO-d_6 as solvent and TMS as an internal standard. High-resolution mass spectra (HRMS) were obtained on an AB 5800 MALDI-TOF/TOF and recorded using electrospray ionization (ESI). The single-crystal X-ray diffraction was conducted in the X-Ray and Spectral Center at Huazhong University of Science and Technology, China.

2 .1 General procedure (GP) for the preparation of *N*-Br sulfoximines[1]

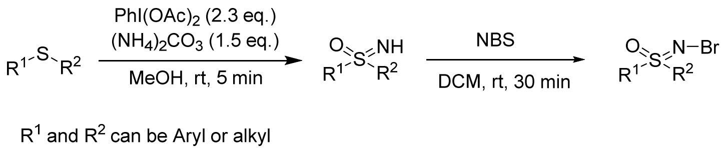

Thioether (10 mmol) was added to a stirring solution of $PhI(OAc)_2$ (23 mmol, 7.40 g) and $(NH_4)_2CO_3$ (15 mmol, 1.44 g) in MeOH (100 mL). Then, the reaction mixture was stirred for another 5 min at room temperature. After the consumption of the thioanisole (monitored by TLC), the reaction solvent was removed under reduced pressure. The crude product was purified by flash column chromatography on SiO_2 to give pure *N*-H sulfoximine as yellow oil.

Sulfoximines (9.6 mmol) were added to a solution of NBS (2.55 g, 14.3 mmol) in 100 mL DCM. The reaction mixture was stirred at room temperature for 30 min. After the consumption of the sulfoximines (monitored by TLC), the reaction solvent was removed under reduced pressure. The crude product was purified by flash column chromatography on SiO_2 to give *N*-Br sulfoximine as the yellow solid.

2.2 General procedure (GP) for the preparation of *N*-aryl indole

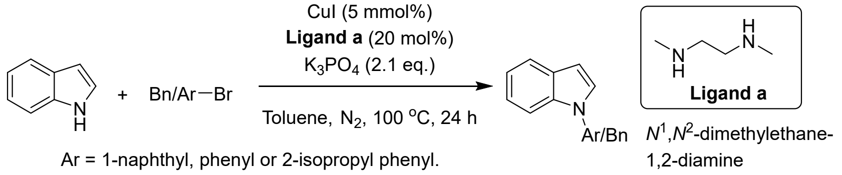

A sealed Schlenk tube with a mixture of CuI (38.1 mg, 0.2 mmol) and K_3PO_4 (1.78 g, 8.4 mmol) inside was evacuated and then filled with N_2 (3 times repeated). Subsequently, indole (468.6 mg, 4 mmol), **Ligand a** (88 μL, 0.8 mmol), Ar/Bn-Br (4.8 mmol) and 2 mL toluene were added into the Schlenk tube under a flow of N_2. The final reaction mixture was vigorously stirred at 110 °C under N_2 atmosphere for 24 h. After the reaction was completed, the reaction mixture was cooled to room temperature. Then, the mixture was diluted with 60 mL H_2O. The aqueous phase was extracted with EtOAc (3 × 30 mL), and the final combined organic phase was dried over Na_2SO_4. The crude product was given after filtration and concentrated in vacuo, then purified by column chromatography on SiO_2 by using PE as eluent to give corresponding *N*-Ar/Bn indoles.

2.3 General procedure (GP1) for the preparation of *N*-alkyl indole

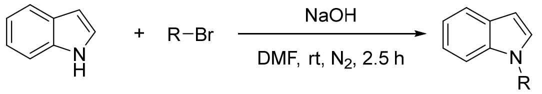

R = $CH_3(CH_2)_3$– , $CH_3(CH_2)_5$– , $CH_3(CH_2)_7$– , $CH_3(CH_2)_9$– , iso-propyl

A mixture of indole (0.59 g, 5.0 mmol), alkyl bromide (5.0 mmol), NaOH (0.40 g, 10.0 mmol) in DMF (10 mL) was vigorously stirred at room temperature under nitrogen atmosphere for 2.5 h. Subsequently, the reaction mixture was diluted with 60 mL H_2O. The aqueous phase was extracted with EtOAc (3 × 30 mL), and the combined organic phase was dried over Na_2SO_4. The crude product was obtained after filtration and concentrated in vacuo and subsequently purified by column chromatography on SiO_2 by using V_{PE} /V_{EA} = 32/1 as eluent to give the desired pure products.

2.4 General procedure (GP2) for the preparation of various substituted *N*-methyl indoles

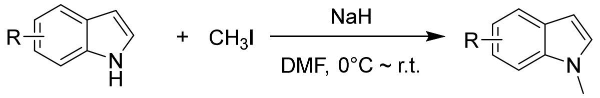

R = H, Cl, Me and Br

To a suspended solution of NaH (180.0 mg, 7.5 mmol) in dry DMF (2.5 mL), the solution of indole (5 mmol) in DMF (2.5 mL) was added dropwise at 0 °C. The final reaction mixture was stirred for 15 min at 0 °C, then for another 1 h at room temperature.

Following that, the mixture was treated with iodomethane (405 μL, 6.5 mmol) at 0 °C, and the final reaction mixture was stirred at room temperature for an additional 30 min. Then, the reaction mixture was quenched with 20 mL NH_4Cl saturated solution at 0 °C. The aqueous solution was extracted with ether (3 × 20 mL). The combined organic layer was washed with brine, dried over anhydrous Na_2SO_4, and concentrated in vacuo. The resulting oil was purified by column chromatography on SiO_2 (V_{PE}/V_{EA} = 32/1) to afford the corresponding product.

2.5 General procedure (GP) for the preparation of product 3

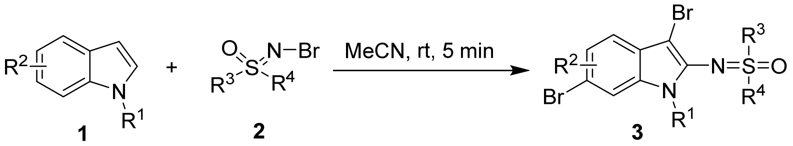

N-Br sulfoximine **2** (1.2 mmol, 3 equiv.) was added to the solution of *N*-substituted indole **1** (0.4 mmol) in MeCN (2 mL) at room temperature. Then, the reaction mixture was stirred at room temperature for another 5 min. Following that, the reaction solvent was concentrated in vacuo and the residue was purified by column chromatography on neutral alumina by using V_{EA}/V_{PE} as eluent to afford the desired product **3**.

2.6 The additional optimization of bromosulfoximination of *N*-Me indole

Firstly, the reaction was investigated under a copper-catalyzed condition. The optimization was evaluated in the presence of a combination of base and copper salt. According to the results, none of the reaction temperature, time, KOAc, or copper salt had any obvious effect on the reaction. Only the amount of *N*-Br sulfoximine has affected the product and its yield. When less than 2 equivalent of the *N*-Br sulfoximine was used in this new protocol, only 2-sulfoximidoyl-3-bromo indole was afforded in 10–65% yield. The mixture was produced when 2 to 2.5 equivalent of *N*-Br sulfoximine was used. Pure 2-sulfoximidoyl-3,6-dibromo indole could be produced in good yields when the 3 equivalent of the *N*-Br sulfoximine was included in this protocol. Compared to the protocol in the absence of base and copper salt, the addition of copper salts would decrease the yields, and the base KOAc would increase the yield slightly, but the amount of *N*-Br sulfoximine played a defining role in the reaction.

Table S1 Additional optimization of bromosulfoximination of *N*-Me indole

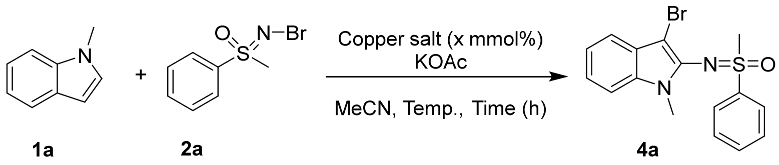

Enty	Temp. (°C)	Time	Base	Catalyst	Equiv. of **2a**	Weight (mg)	Yield %
1	r.t.	1 h	KOAc	5 mol% CuBr	1	42.2	29
2	r.t.	1.5 h	KOAc	5 mol% CuBr	1	40.7	28
3	r.t.	4 h	KOAc	5 mol% CuBr	1	28.5	20
4	r.t.	4 h	KOAc	10 mol% CuBr	1	32.3	22
5	r.t.	4 h	KOAc	20 mol% CuBr	1	14.4	10
6	r.t.	30 min	KOAc	5mol% CuBr	1	37.9	26
7	70	1 h	KOAc	5 mol% CuBr	1	21.3	20
8	70	1 h	KOAc	5 mol% CuBr	1	31.9	22
9	70	1 h	KOAc	5 mol% CuBr	1	23.3	16
10	70	12 h	None	10 mol% $Cu(OAc)_2$	1	39.2	27
11	30	5 min	KOAc	10 mol% CuBr	1.5	66.5	46
12	30	5 min	KOAc	10 mol% CuBr	2	94.5	65

13	30	5 min	KOAc	10 mol% CuBr	3	134.0	76 [b]
14	30	5 min	KOAc	10 mol% CuCl	3	140.2	79 [b]
15	30	5 min	KOAc	10 mol% CuI	3	132.0	75 [b]
16	30	5 min	KOAc	10 mol% $CuCl_2$	3	143.6	81 [b]
17	30	5 min	KOAc	10 mol% $CuBr_2$	3	141.0	80 [b]
18	30	5 min	KOAc	None	3	159.2	90 [b]
19	30	5 min	None	None	3	156	88 [b]

[a] Unless otherwise noted, the reactions were performed with indicated amounts of *N*-Br methyl phenyl sulfoximine **2a** added to the solution of 0.4 mmol *N*-Me indole in 2 mL MeCN at indicated temperatures. All yields were isolated. **[b]** The product was 2-sulfoximidoyl-3,6-dibrom indole.

3 Characterization Data[3]

1-Butyl-1H-indole (1a)

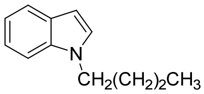

According to the general procedure (GP1) for the preparation of *N*-alkyl indole, the 1-butyl-1H-indole was prepared as yellow oil in 97% yield.

^{1}H NMR (400 MHz, chloroform-*d*) δ 7.61 (dq, *J* = 7.8, 1.0 Hz, 1H), 7.29 (dq, *J* = 8.3, 0.9 Hz, 1H), 7.17 (ddt, *J* = 8.2, 7.0, 1.5 Hz, 1H), 7.07 (ddt, *J* = 7.9, 6.9, 1.0 Hz, 1H), 7.02 – 6.98 (m, 1H), 6.45 (dt, *J* = 3.1, 0.9 Hz, 1H), 4.00 (t, *J* = 7.1 Hz, 2H), 1.72 (tt, *J* = 9.1, 6.8 Hz, 2H), 1.26 (dtd, *J* = 16.7, 8.3, 7.9, 6.7 Hz, 2H), 0.88 (td, *J* = 7.3, 1.4 Hz, 3H).

1-Hexyl-1H-indole (1b)

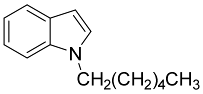

According to the general procedure (GP1) for the preparation of *N*-alkyl indole, the 1-hexyl-1H-indole was prepared as yellow oil in 95% yield.

^{1}H NMR (400 MHz, chloroform-*d*) δ 7.63 (dt, *J* = 7.9, 1.0 Hz, 1H), 7.34 (dd, *J* = 8.3, 1.1 Hz, 1H), 7.20 (ddd, *J* = 8.2, 6.9, 1.2 Hz, 1H), 7.12 – 7.06 (m, 2H), 6.48

(d, J = 3.3 Hz, 1H), 4.10 (t, J = 7.2 Hz, 2H), 1.82 (s, 2H), 1.30 (q, J = 4.3, 3.3 Hz, 6H), 0.92 – 0.82 (m, 3H).

1-Octyl-1H-indole (1c)

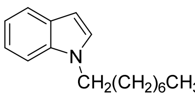

According to the general procedure (GP1) for the preparation of *N*-alkyl indole, the 1-octyl-1H-indole was prepared as yellow oil in 96% yield. ^{1}H NMR (400 MHz, chloroform-*d*) δ 7.60 (d, J = 7.9 Hz, 1H), 7.29 (d, J = 8.3 Hz, 1H), 7.17 (t, J = 7.6 Hz, 1H), 7.11 – 6.99 (m, 2H), 6.45 (d, J = 3.1 Hz, 1H), 4.02 (t, J = 7.2 Hz, 2H), 1.77 (t, J = 7.0 Hz, 2H), 1.25 (dd, J = 12.9, 5.8 Hz, 10H), 0.86 (t, J = 6.7 Hz, 3H).

1-Decyl-1H-indole (1d)

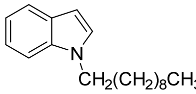

According to the general procedure (GP1) for the preparation of *N*-alkyl indole, the 1-octyl-1H-indole was prepared as yellow oil in 95% yield. ^{1}H NMR (400 MHz, chloroform-*d*) δ 7.62 (dt, J = 7.9, 1.0 Hz, 1H), 7.32 (dq, J = 8.2, 0.9 Hz, 1H), 7.19 – 7.16 (m, 1H), 7.11 – 7.08 (m, 1H), 7.08 – 7.06 (m, 1H), 6.47 (dd, J = 3.2, 0.9 Hz, 1H), 4.07 (t, J = 7.2 Hz, 2H), 1.85 – 1.75 (m, 2H), 1.31 – 1.18 (m, 14H), 0.90 – 0.85 (m, 3H).

1,4-Dimethyl-1H-indole (1e)

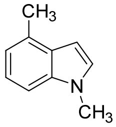

According to the general procedure (GP2) for the preparation of *N*-alkyl indole, the 1-octyl-1H-indole was prepared as yellow oil in 64% yield. ^{1}H NMR (400 MHz, chloroform-*d*) δ 7.13 – 7.09 (m, 2H), 6.96 (d, J = 3.2 Hz, 1H), 6.89 (td, J = 4.0, 0.9 Hz, 1H), 6.47 (dd, J = 3.2, 0.6 Hz, 1H), 3.67 (s, 3H), 2.54 (d, J = 1.1 Hz, 3H).

1,7-Dimethyl-1H-indole (1f)

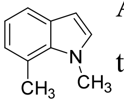

According to the general procedure (GP2) for the preparation of *N*-alkyl indole, the 1-octyl-1H-indole was prepared as white oil in 80% yield. ^{1}H NMR (400 MHz, chloroform-*d*) δ 7.43 (ddd, J = 7.8, 1.3, 0.7 Hz, 1H), 6.95 (dd, J = 7.8, 7.1 Hz, 1H), 6.90 – 6.86 (m, 2H), 6.41 (d, J = 3.1 Hz, 1H), 4.00 (s, 3H), 2.74 (d, J = 0.8 Hz, 3H).

7-Chloro-1-methyl-1H-indole (1g)

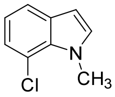

According to the general procedure (GP2) for the preparation of *N*-alkyl indole, the 1-octyl-1H-indole was prepared as white solid in 93% yield. ^{1}H NMR (400 MHz, chloroform-*d*) δ 7.47 (dd, *J* = 7.9, 1.0 Hz, 1H), 7.12 (dd, *J* = 7.6, 1.0 Hz, 1H), 6.98 – 6.92 (m, 2H), 6.44 (d, *J* = 3.1 Hz, 1H), 4.10 (s, 3H).

7-Bromo-1-methyl-1H-indole (1h)

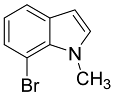

According to the general procedure (GP2) for the preparation of *N*-alkyl indole, the 1-octyl-1H-indole was prepared as yellow solid in 82% yield. ^{1}H NMR (400 MHz, chloroform-*d*) δ 7.52 (dd, *J* = 7.8, 1.0 Hz, 1H), 7.33 (dd, *J* = 7.6, 1.0 Hz, 1H), 6.97 (d, *J* = 3.2 Hz, 1H), 6.90 (t, *J* = 7.7 Hz, 1H), 6.44 (d, *J* = 3.1 Hz, 1H), 4.14 (s, 3H).

1-Phenyl-1H-indole (1i)

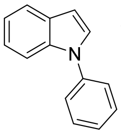

According to the general procedure (GP) for the preparation of *N*-aryl indole, the 1-phenyl-1H-indole was prepared as yellow oil in 56% yield. ^{1}H NMR (400 MHz, chloroform-*d*) δ 7.72 – 7.64 (m, 1H), 7.60 – 7.52 (m, 1H), 7.48 (d, *J* = 4.4 Hz, 4H), 7.32 (p, *J* = 4.6 Hz, 2H), 7.25 – 7.11 (m, 2H), 6.67 (dd, *J* = 3.3, 0.8 Hz, 1H).

1-Benzyl-1H-indole (1j)

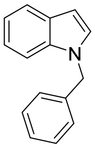

According to the general procedure (GP) for the preparation of *N*-aryl indole, the 1-benzyl-1H-indole was prepared as white solid in 83% yield. ^{1}H NMR (400 MHz, chloroform-*d*) δ 8.03 – 7.98 (m, 1H), 7.59 – 7.43 (m, 6H), 7.34 (q, *J* = 2.9 Hz, 3H), 6.88 (dd, *J* = 3.1, 0.9 Hz, 1H), 5.44 (s, 2H).

1-(2-Isopropylphenyl)-1H-indole (1k)

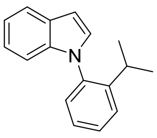

According to the general procedure (GP) for the preparation of *N*-aryl indole, the 1-(2-isopropylphenyl)-1H-indole was prepared as red oil in 50% yield. ^{1}H NMR (400 MHz, chloroform-*d*) δ 7.71 – 7.63 (m, 1H), 7.49 – 7.36 (m, 2H), 7.28 – 7.19 (m, 2H), 7.17 – 7.07 (m, 3H), 7.04 – 6.98 (m, 1H), 6.65 (dd, *J* =

3.2, 0.9 Hz, 1H), 2.64 (hept, J = 6.9 Hz, 1H), 1.08 (dd, J = 20.6, 6.8 Hz, 6H).

1-(Naphthalen-1-yl)-1H-indole (1l)

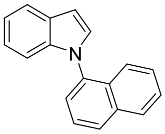

According to the general procedure (GP) for the preparation of *N*-aryl indole, the 1-(2-isopropylphenyl)-1H-indole was prepared as light blue solid in 52% yield. ^{1}H NMR (400 MHz, chloroform-*d*) δ 7.99 – 7.90 (m, 2H), 7.74 (dt, J = 8.0, 1.0 Hz, 1H), 7.60 – 7.48 (m, 3H), 7.47 – 7.31 (m, 3H), 7.24 – 7.08 (m, 3H), 7.02 (dd, J = 8.4, 1.2 Hz, 1H), 6.76 (dd, J = 3.2, 0.9 Hz, 1H).

1-Isopropyl-1H-indole (1m)

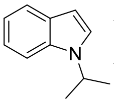

According to the general procedure (GP1) for the preparation of N-alkyl indole, the 1-isopropyl-1H-indole was prepared as yellow oil in 76% yield. ^{1}H NMR (400 MHz, chloroform-*d*) δ 7.61 (dt, J = 7.9, 1.0 Hz, 1H), 7.32 (dd, J = 8.4, 1.1 Hz, 1H), 7.20 – 7.16 (m, 1H), 7.16 – 7.13 (m, 1H), 7.08 (ddd, J = 7.9, 6.9, 1.0 Hz, 1H), 6.48 (dd, J = 3.3, 0.9 Hz, 1H), 4.57 (hept, J = 6.7 Hz, 1H), 1.43 (d, J = 6.8 Hz, 6H).

***N*-Br methyl phenylsulfoximine (2a)**

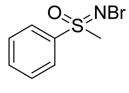

According to the general procedure (GP) for the preparation of N-bromosulfoximines, the *N*-Br methyl phenylsulfoximine was prepared as yellow oil in 76% yield. ^{1}H NMR (400 MHz, chloroform-*d*) δ 7.94-7.88 (m, 2H), 7.73-7.66 (m, 1H), 7.65-7.58 (m, 2H), 3.30 (s, 3H).

***N*-Br methyl *p*-tolylsulfoximine (2b)**

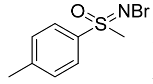

According to the general procedure (GP) for the preparation of *N*-bromosulfoximines, the *N*-Br methyl *p*-tolylsulfoximine was prepared as yellow solid in 81% yield. ^{1}H NMR (400 MHz, chloroform-*d*) δ 7.80 (d, J = 8.0 Hz, 2H), 7.40 (d, J = 8.0 Hz, 2H), 3.26 (s, 3H), 2.47 (s, 3H).

***N*-Br methyl 4-chlorophenylsulfoximine (2c)**

According to the general procedure (GP) for the preparation of *N*-bromosulfoximines,

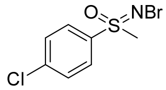
the *N*-Br methyl 4-chlorophenylsulfoximine was prepared as yellow solid in 90% yield. ^{1}H NMR (400 MHz, chloroform-*d*) δ 7.85 (d, *J* = 8.6 Hz, 2H), 7.60 (d, *J* = 8.6 Hz, 2H), 3.32 (s, 3H).

N-Br methyl 4-bromophenylsulfoximine (2d)

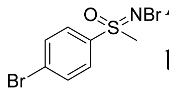
According to the general procedure (GP) for the preparation of *N*-bromosulfoximines, the *N*-Br methyl 4-bromophenylsulfoximine was prepared as yellow solid in 86% yield. ^{1}H NMR (400 MHz, chloroform-*d*) δ 7.77 (s, 4H), 3.31 (s, 3H).

N-Br methyl 4-nitrophenylsulfoximine (2e)

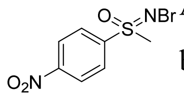
According to the general procedure (GP) for the preparation of *N*-bromosulfoximines, the *N*-Br methyl 4-nitrophenylsulfoximine was prepared as yellow solid in 52% yield. ^{1}H NMR (400 MHz, chloroform-*d*) δ 8.49-8.44 (m, 2H), 8.15-8.10 (m, 2H), 3.38 (s, 3H).

N-Br methyl 3-bromophenylsulfoximine (2f)

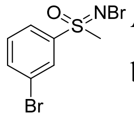
According to the general procedure (GP) for the preparation of *N*-bromosulfoximines, the *N*-Br methyl 3-bromophenylsulfoximine was prepared as yellow solid in 87% yield. ^{1}H NMR (400 MHz, chloroform-*d*) δ 8.06 (t, *J* = 1.8 Hz, 1H), 7.83 (dddd, *J* = 8.1, 7.2, 1.9, 1.0 Hz, 2H), 7.54-7.47 (m, 1H), 3.32 (s, 3H).

N-Br methyl 3-chlorophenylsulfoximine (2g)

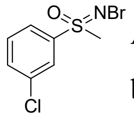
According to the general procedure (GP) for the preparation of *N*-bromosulfoximines, the *N*-Br methyl 3-chlorophenylsulfoximine was prepared as yellow solid in 72% yield. ^{1}H NMR (400 MHz, chloroform-*d*) δ 7.92-7.90 (m, 1H), 7.80 (ddd, *J* = 7.8, 1.8, 1.1 Hz, 1H), 7.67 (ddd, *J* = 8.0, 2.1, 1.1 Hz, 1H), 7.60-7.55 (m, 1H), 3.32 (s, 3H).

N-Br methyl 2-bromophenylsulfoximine (2h)

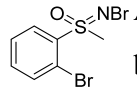

According to the general procedure (GP) for the preparation of *N*-bromosulfoximines, the *N*-Br methyl 2-bromophenylsulfoximine was prepared as yellow solid in 82% yield. ^{1}H NMR (400 MHz, chloroform-*d*) *δ* 8.24 (dd, *J* = 7.9, 1.8 Hz, 1H), 7.81 (dd, *J* = 7.8, 1.4 Hz, 1H), 7.62-7.55 (m, 1H), 7.51 (td, *J* = 7.6, 1.8 Hz, 1H), 3.52 (s, 3H).

N-Br methyl 2-chlorophenylsulfoximine (2i)

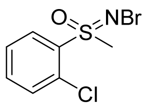

According to the general procedure (GP) for the preparation of *N*-bromosulfoximines, the *N*-Br methyl 2-chlorophenylsulfoximine was prepared as yellow solid in 78% yield. ^{1}H NMR (400 MHz, chloroform-*d*) δ 8.19 (ddd, *J* = 7.9, 1.6, 0.5 Hz, 1H), 7.64 – 7.58 (m, 2H), 7.54 (ddd, *J* = 7.9, 6.7, 2.0 Hz, 1H), 3.51 (s, 3H).

N-Br diphenylsulfoximine (2j)

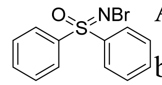

According to the general procedure (GP) for the preparation of *N*-bromosulfoximines, the *N*-Br diphenylsulfoximine was prepared as yellow solid in 72% yield. ^{1}H NMR (400 MHz, chloroform-*d*) *δ* 8.01-7.96 (m, 4H), 7.63-7.51 (m, 6H).

N-Br isopropyl phenylsulfoximine (2k)

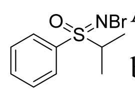

According to the general procedure (GP) for the preparation of *N*-bromosulfoximines, the *N*-Br isopropyl phenylsulfoximine was prepared as yellow solid in 57% yield. ^{1}H NMR (400 MHz, chloroform-*d*) *δ* 7.88-7.81 (m, 2H), 7.73-7.67 (m, 1H), 7.65-7.58 (m, 2H), 3.62 (p, *J* = 6.9 Hz, 1H), 1.47-1.40 (m, 3H), 1.28 (dd, *J* = 6.9, 1.0 Hz, 3H).

N-Br butyl phenylsulfoximine (2l)

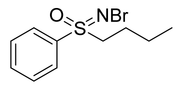

According to the general procedure (GP) for the preparation of *N*-bromosulfoximines, the *N*-Br butyl phenylsulfoximine was prepared as yellow oil in 52% yield. ^{1}H NMR (400 MHz, chloroform-*d*) *δ* 7.88 (d, *J* = 7.5 Hz, 2H), 7.70 (t, *J* = 7.4 Hz, 1H), 7.62 (t, *J* = 7.6 Hz, 2H), 3.40 (dddd, *J* = 51.1, 14.0, 11.0, 5.2 Hz, 2H), 1.87-1.57 (m, 2H), 1.44-1.32 (m, 2H), 0.89 (t, *J* = 7.4 Hz, 3H).

***N*-Br dibutylsulfoximine (2m)**

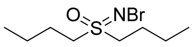

According to the general procedure (GP) for the preparation of *N*-bromosulfoximines, the *N*-Br dibutylsulfoximine was prepared as yellow oil in 39% yield. ^{1}H NMR (400 MHz, chloroform-*d*) δ 3.21 (hept, J = 7.2 Hz, 4H), 1.75 (qd, J = 8.5, 6.6 Hz, 4H), 1.45 (h, J = 7.4 Hz, 4H), 0.94 (t, J = 7.4 Hz, 6H).

4. X-Ray Crystallographic Data

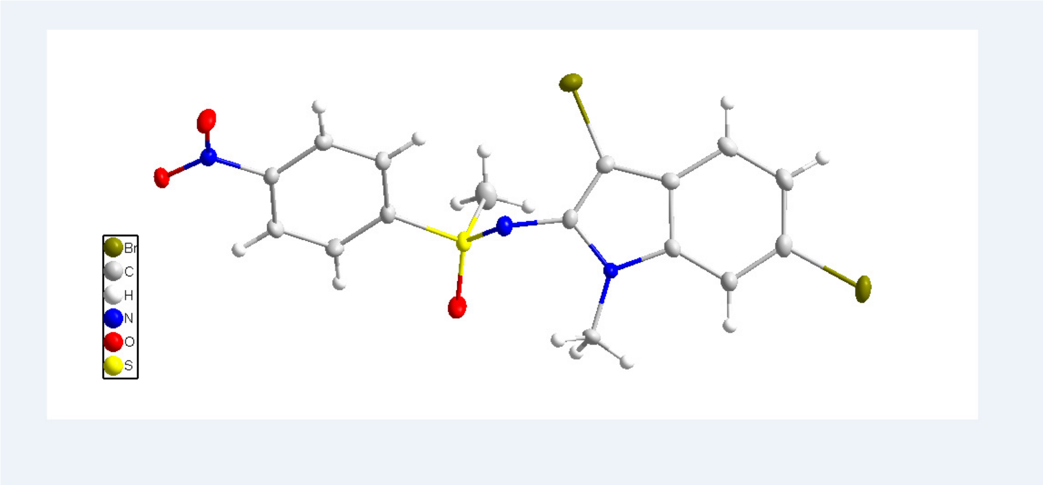

Table S2 Crystal data and structure refinement

Identification code	3bd
Empirical formula	$C_{16}H_{13}Br_2N_3O_3S$
Formula weight	487.17
Temperature/K	293(2)
Crystal system	monoclinic
Space group	$P2_1/c$
a/Å	14.1638(2)
b/Å	11.04160(10)
c/Å	11.36030(10)
α/°	90
β/°	90.3890(10)
γ/°	90

Volume/Å^3	1776.61(3)
Z	4
ρ_{calc}g/cm^3	1.821
μ/mm^{-1}	7.080
F(000)	960.0
Radiation	CuKα (λ = 1.54178)
2θ range for data collection/°	6.24 to 148.15
Index ranges	$-17 \leq h \leq 17$, $-9 \leq k \leq 13$, $-14 \leq l \leq 13$
Reflections collected	17436
Independent reflections	3562 [R_{int} = 0.0652, R_{sigma} = 0.0300]
Data/restraints/parameters	3562/0/228
Goodness of fit on F^2	0.932
Final R indices [I>=2σ (I)]	R_1 = 0.0465, wR_2 = 0.1290
Final R indices [all data]	R_1 = 0.0467, wR_2 = 0.1293
Largest diff. peak/hole / e Å^{-3}	1.89/-1.04

5. Reference

[1] (a) Xie, Y.; Zhou, B.; Zhou, S.; Zhou, S.; Wei, W.; Liu, J.; Zhan, Y.; Cheng, D.; Chen, M.; Li, Y.; Wang, B.; Xue, X.-s.; Li, Z., *ChemistrySelect* 2017**,** *2*, 1620; (b) Bohnen, C.; Bolm, C., *Org. Lett.* 2015**,** *17*, 3011.

[2] (a) Hong, X.; Tan, Q.; Liu, B.; Xu, B., *Angew. Chem. Int. Ed.* 2017**,** *56*, 3961; (b) Antilla, J. C.; Klapars, A.; Buchwald, S. L., *J. Am. Chem. Soc.* 2002**,** *124*, 11684.

[3] (a) Wang, C.; Shi, P.; Bolm, C. *Org. Chem, Fron.* 2021, *8,* 2919; (b) Banjare, S. K.; Nanda, T.; Pati, B. V.; Adhikari, G. K. D.; Dutta, J.; Ravikumar, P. C. *ACS Catalysis* 2021, *11*, 11579; (c) Das, D.; Bhutia, Z. T.; Chatterjee, A.; Banerjee, M. *J. Org. Chem.* 2019, *84*, 10764; (d) Bohnen, C.; Bolm, C. *Org. Lett.* 2015, *17*, 3011.

7 NMR Spectra

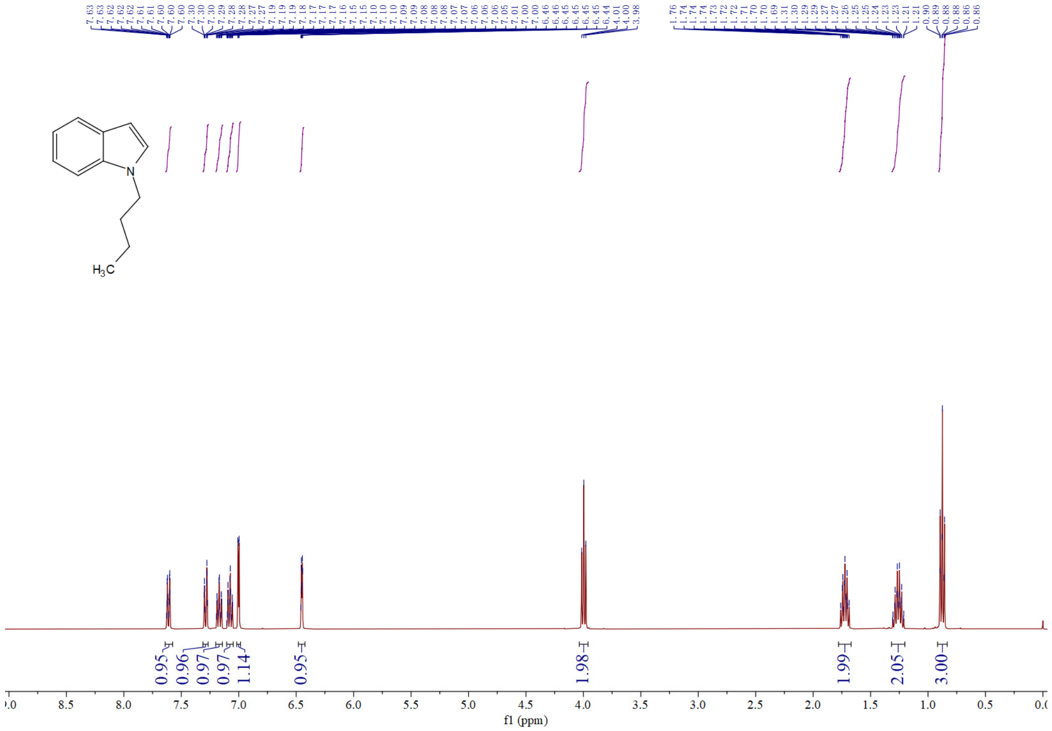
N
H3C
0.95
0.96
0.97
0.97
1.14
0.95
1.98
1.99
2.05
3.00
f1 (ppm)

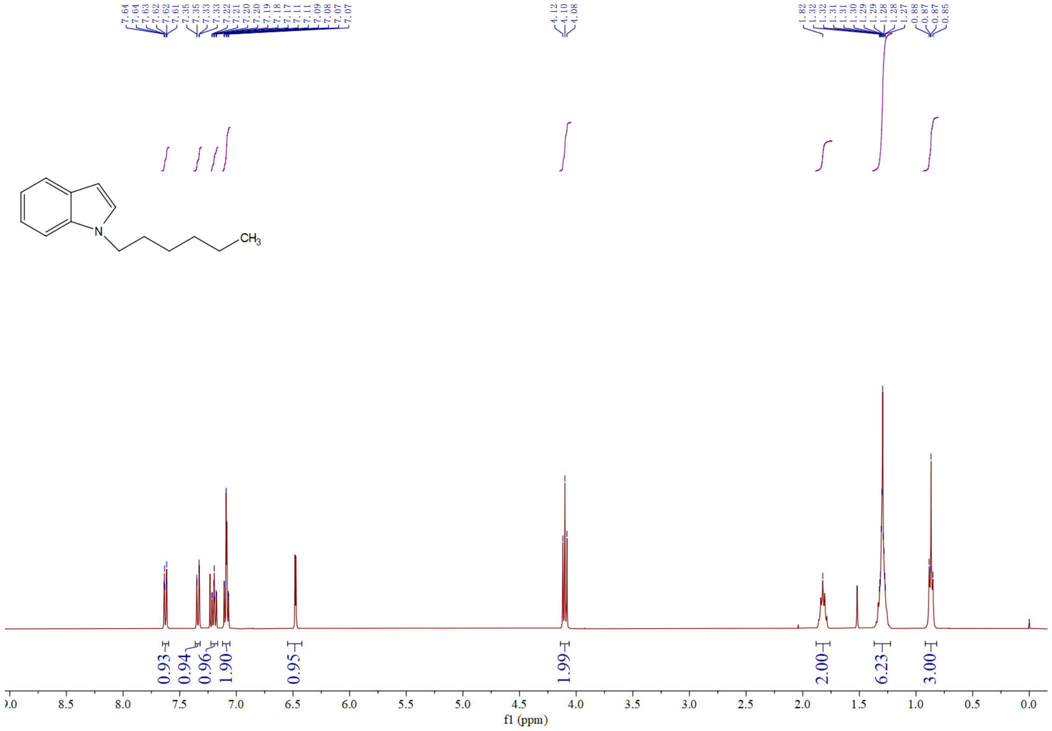
N
CH3
0.93
0.94
0.96
1.90
0.95
1.99
2.00
6.23
3.00
f1 (ppm)

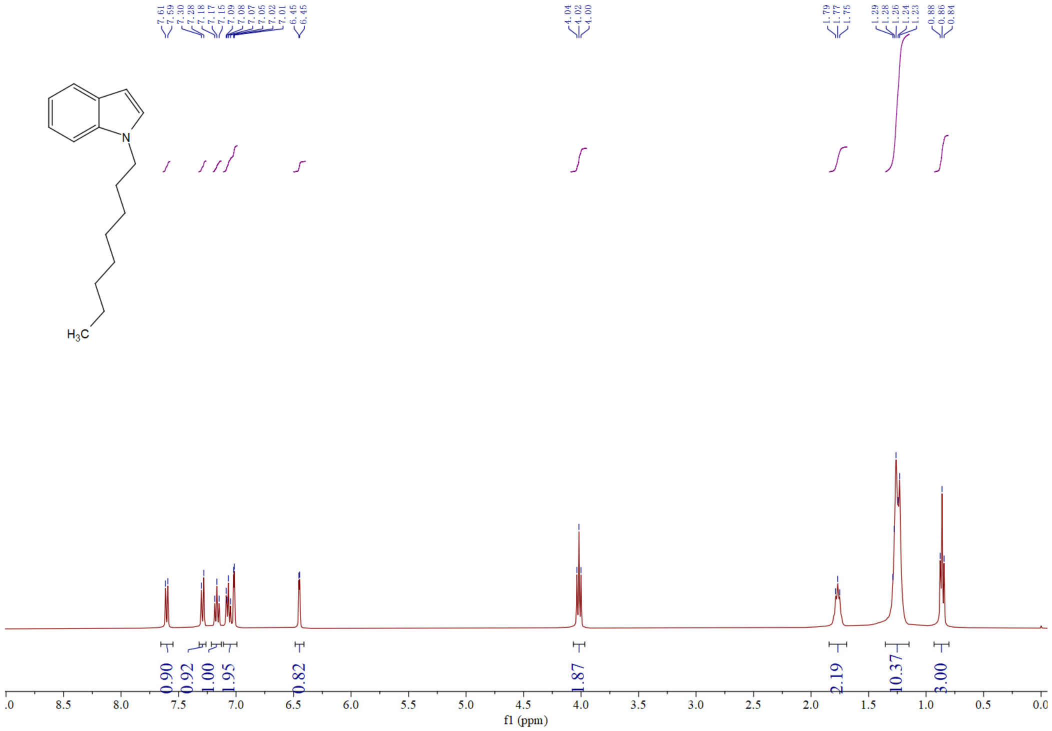

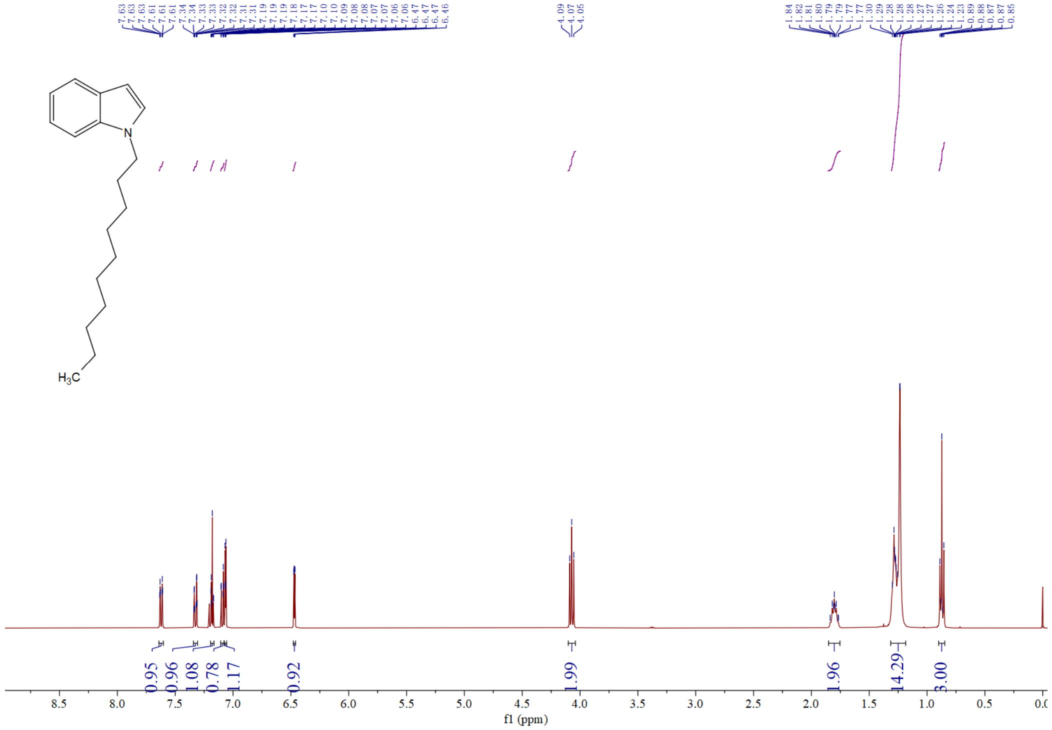

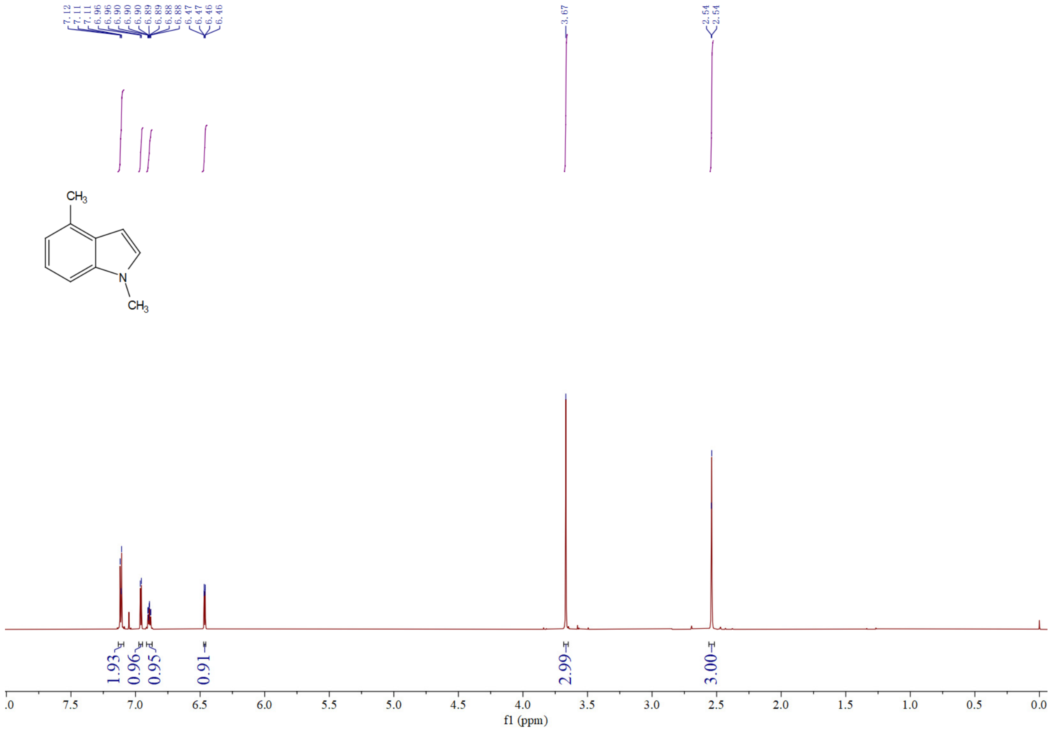

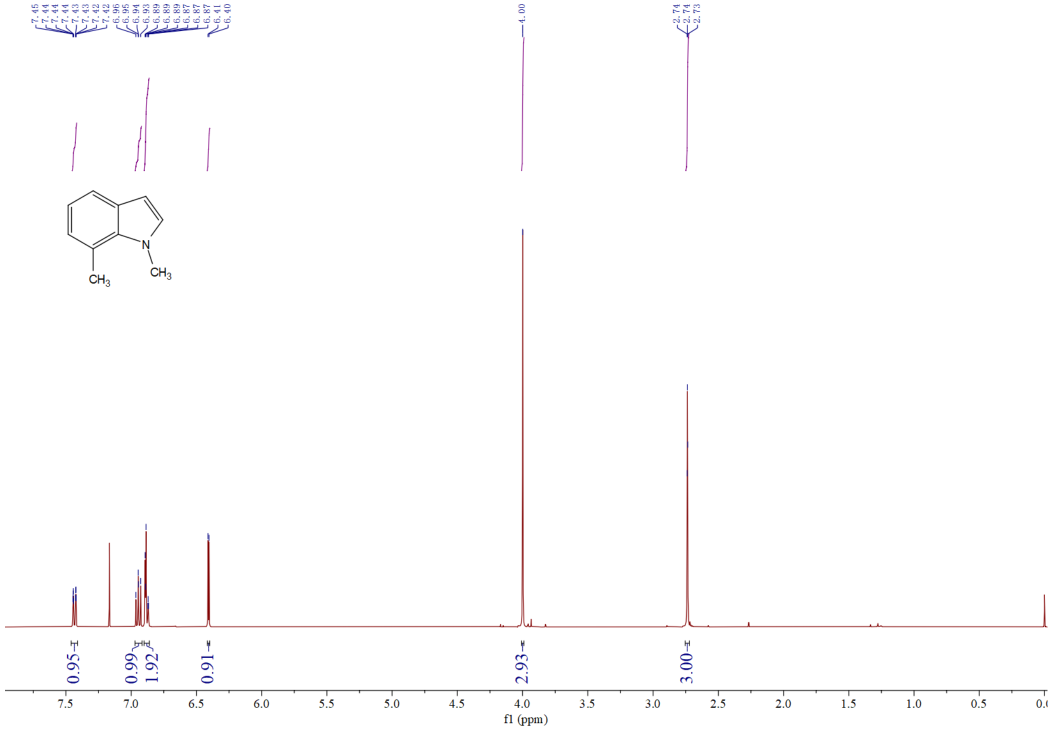

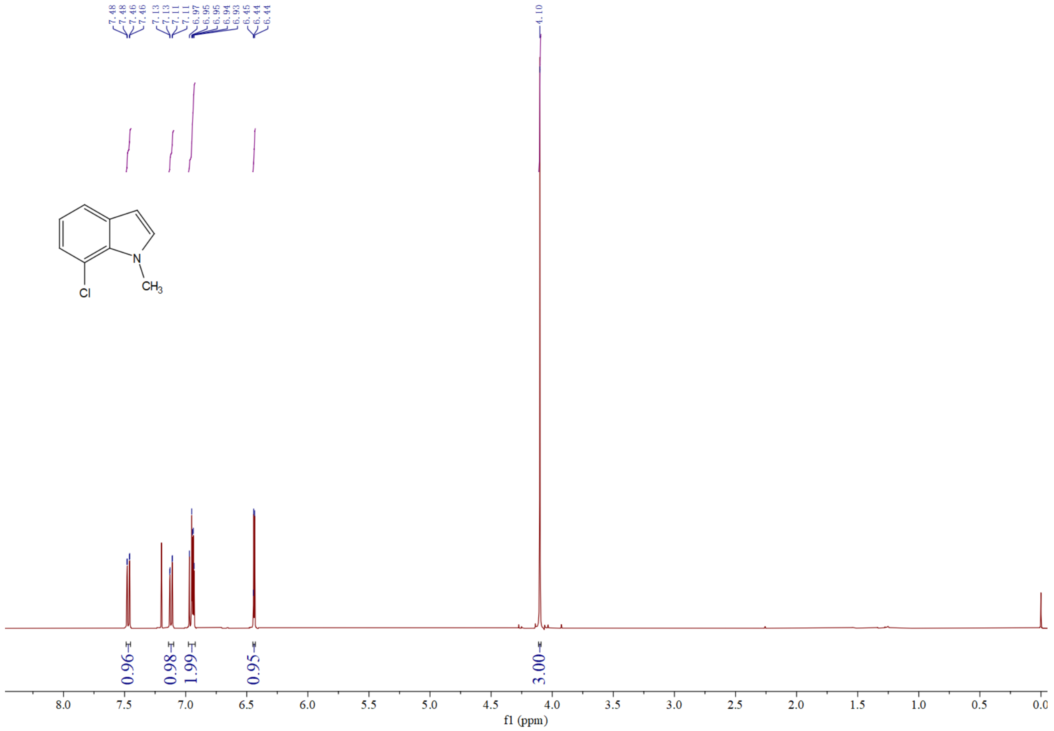
7.48
7.48
7.46
7.46
7.13
7.13
7.11
7.11
6.97
6.95
6.95
6.94
6.93
6.45
6.44
6.44
4.10
N
CH3
Cl
0.96
0.98
1.99
0.95
3.00
f1 (ppm)

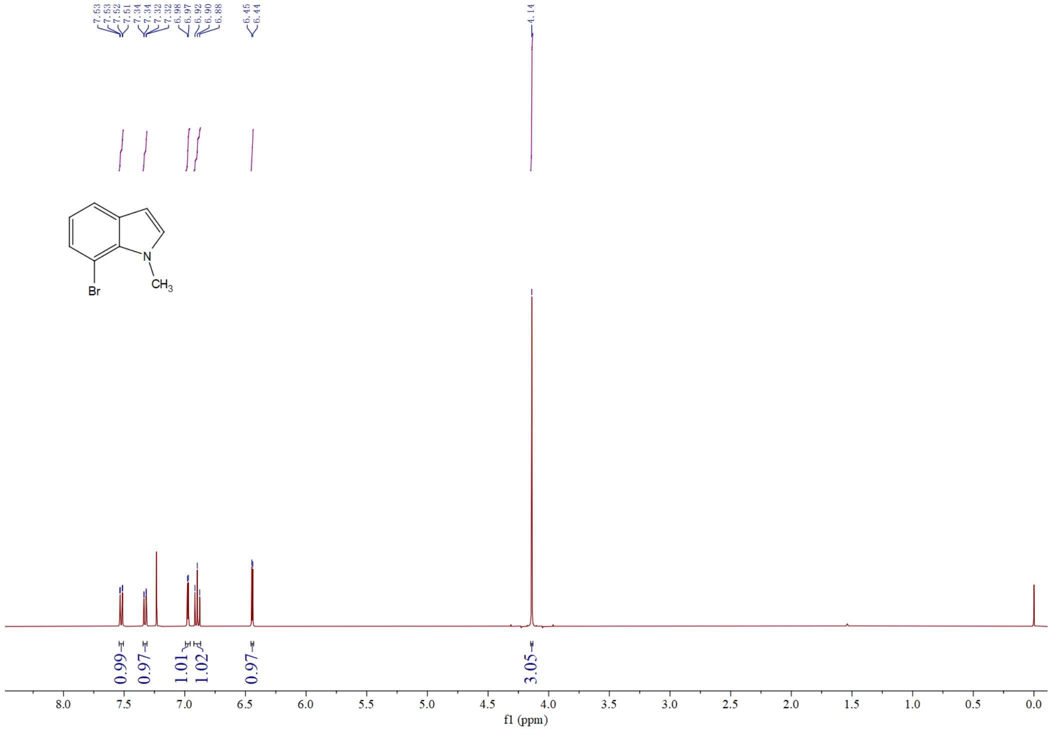
7.53
7.53
7.52
7.51
7.34
7.34
7.32
7.32
6.98
6.97
6.92
6.90
6.88
6.45
6.44
4.14
N
CH3
Br
0.99
0.97
1.01
1.02
0.97
3.05
f1 (ppm)

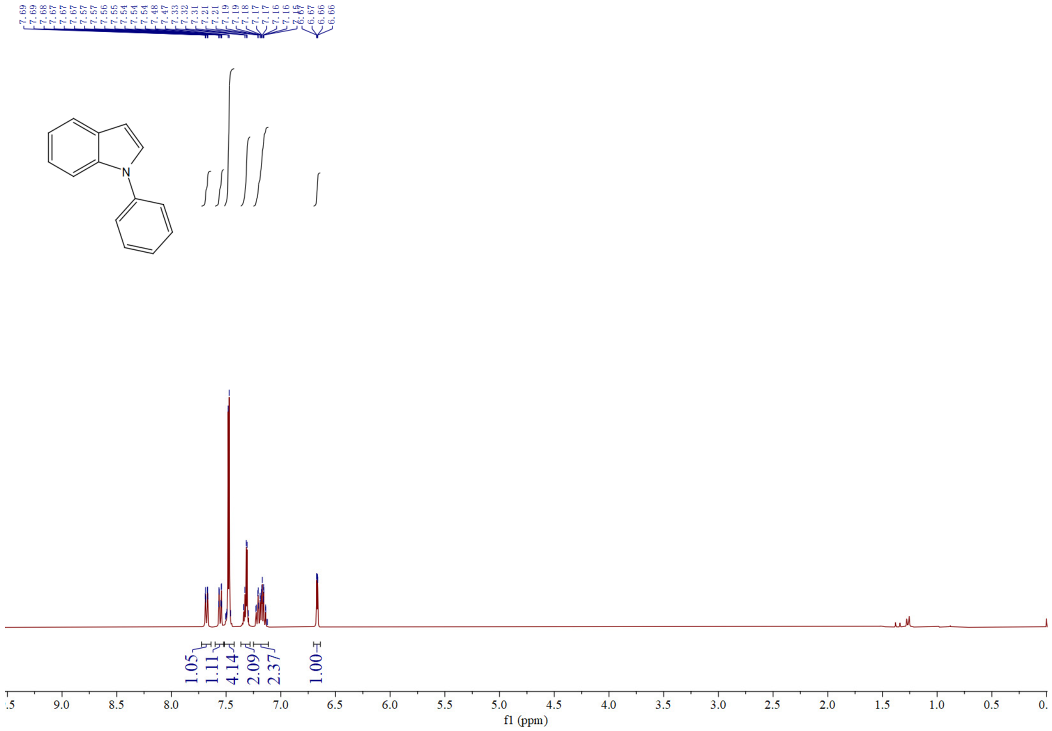
1.05
1.11
4.14
2.09
2.37
1.00
f1 (ppm)

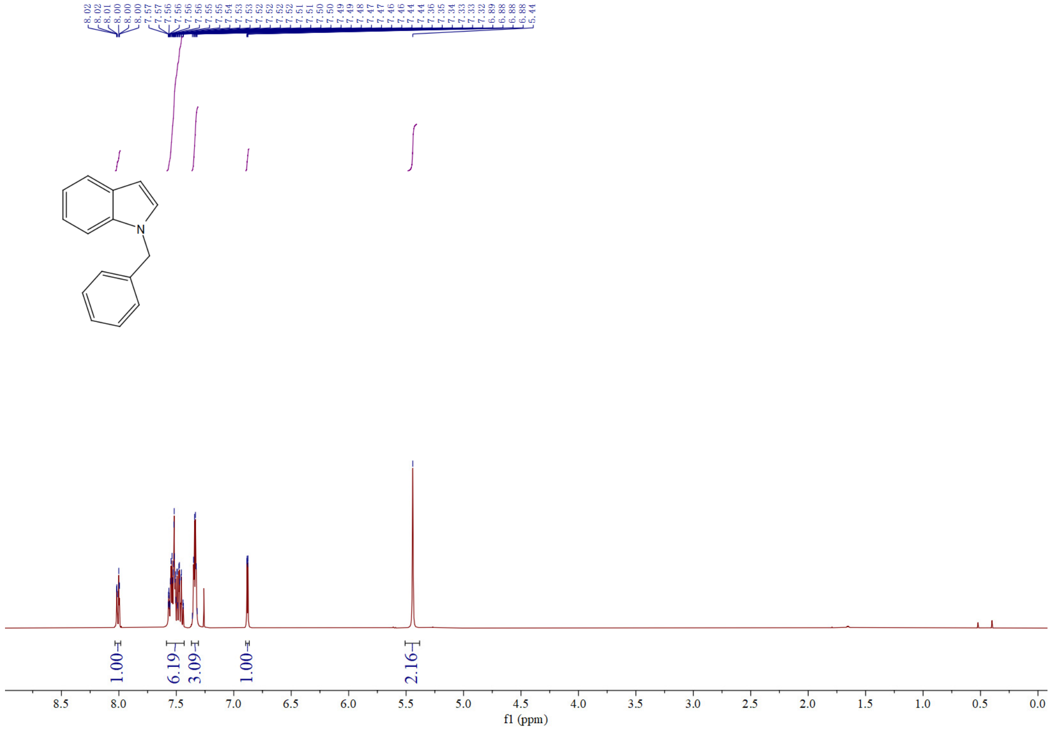
1.00
6.19
3.09
1.00
2.16
f1 (ppm)

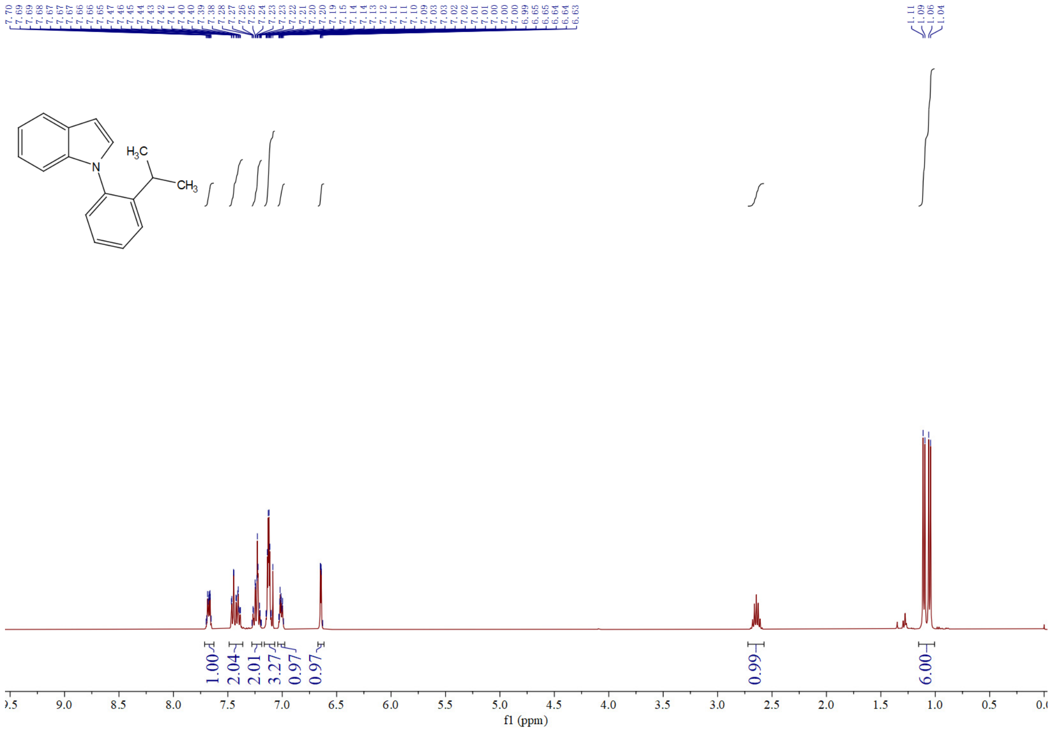
N
H3C
CH3
1.00
2.04
2.01
3.27
0.97
0.97
0.99
6.00
f1 (ppm)

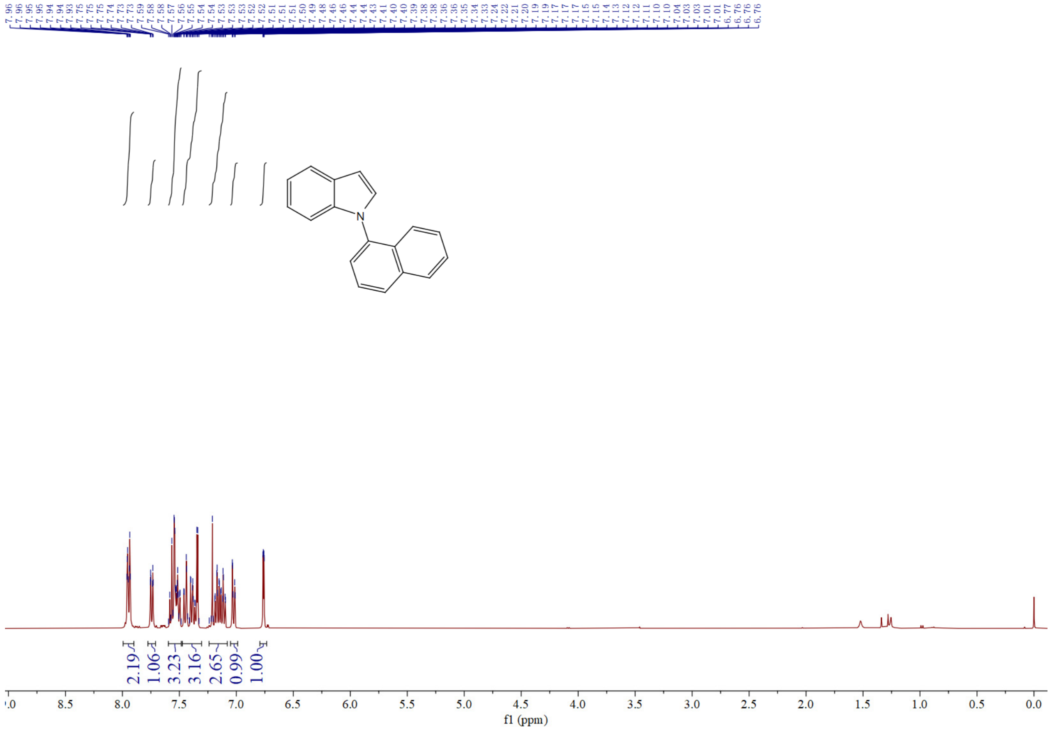
N
2.19
1.06
3.23
3.16
2.65
0.99
1.00
f1 (ppm)

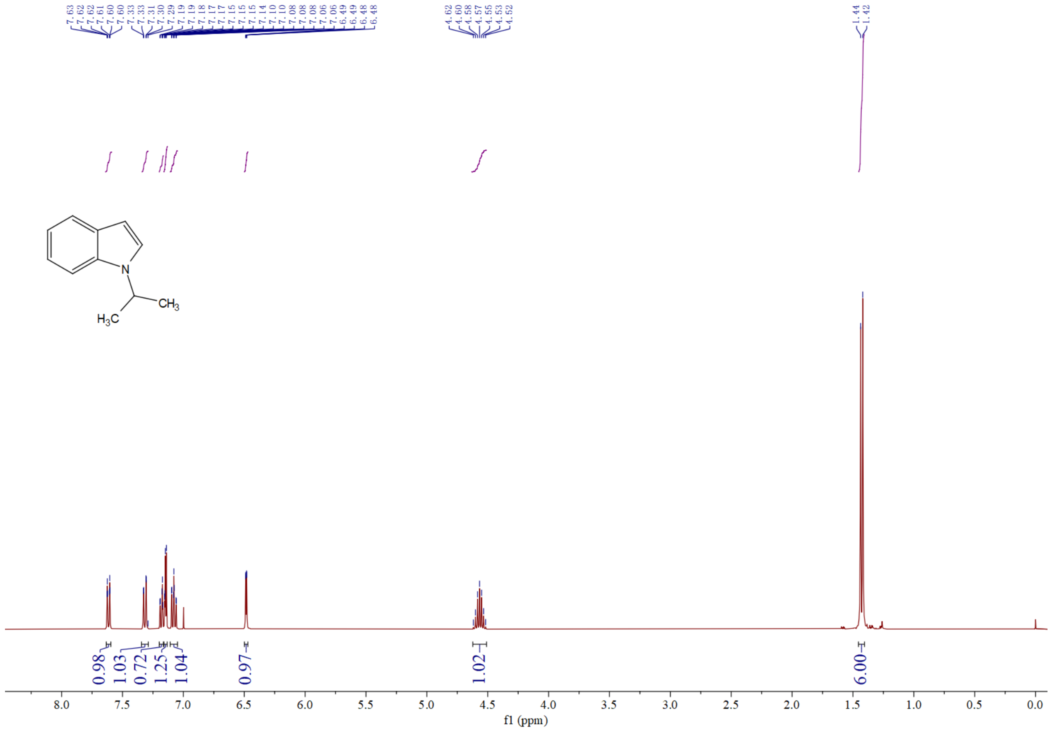
N
CH3
H3C
f1 (ppm)

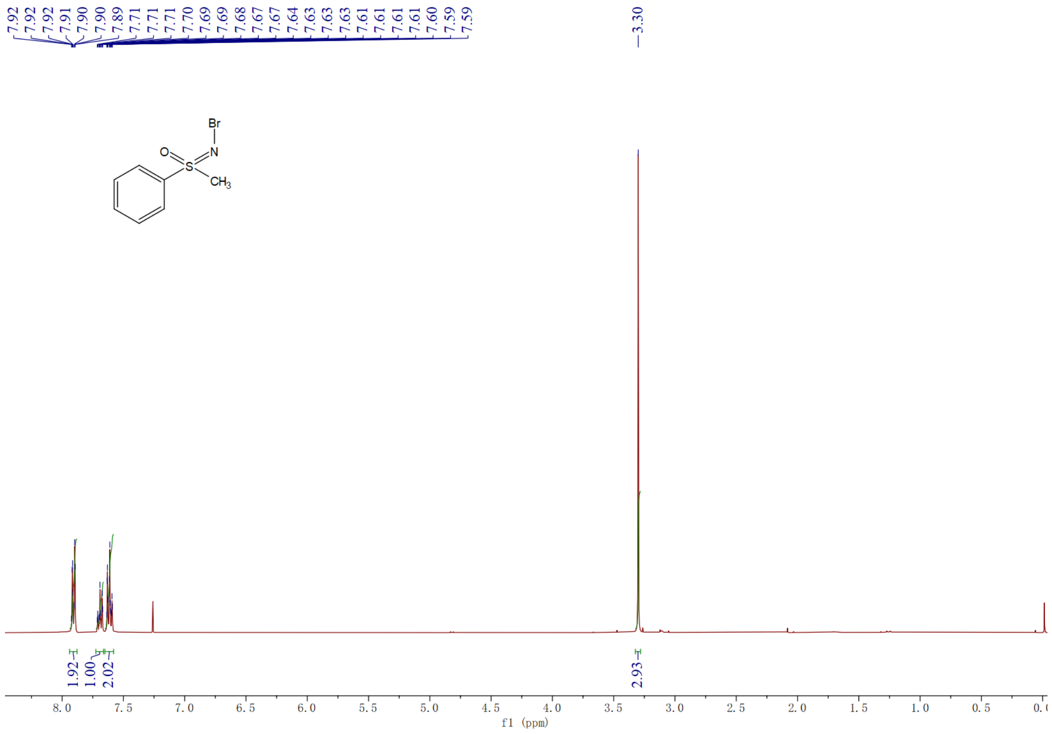
Br
N
O
S
CH3
f1 (ppm)

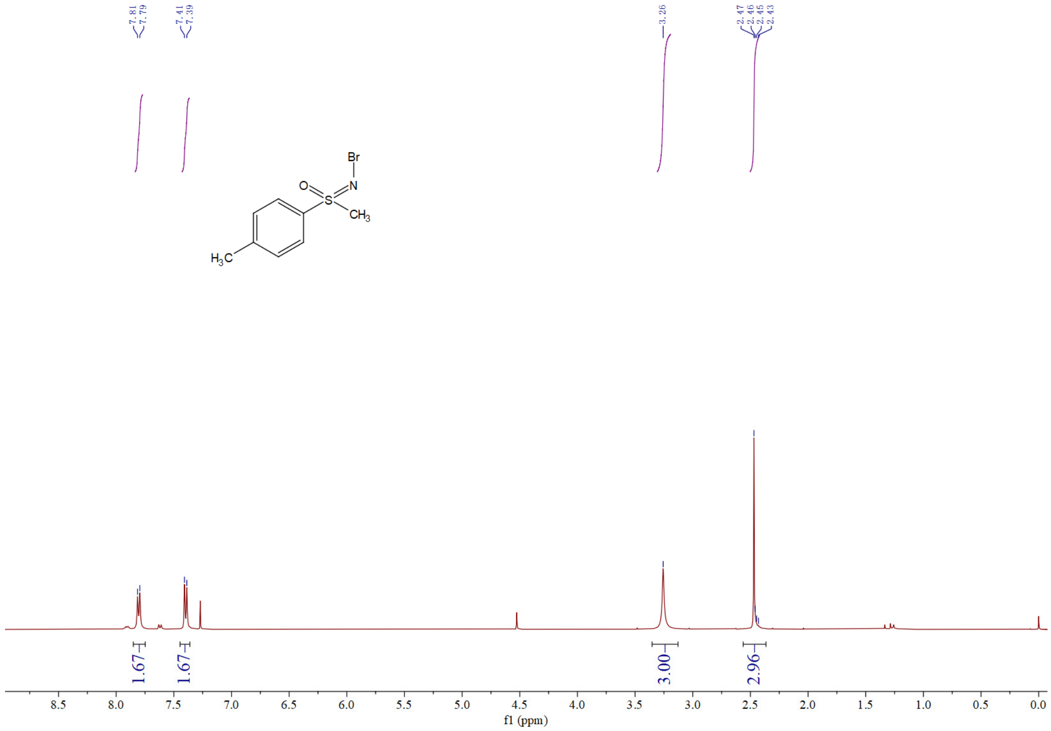
7.81
7.79
7.41
7.39
3.26
2.47
2.46
2.45
2.43
Br
O
S
N
CH3
H3C
1.67
1.67
3.00
2.96
f1 (ppm)

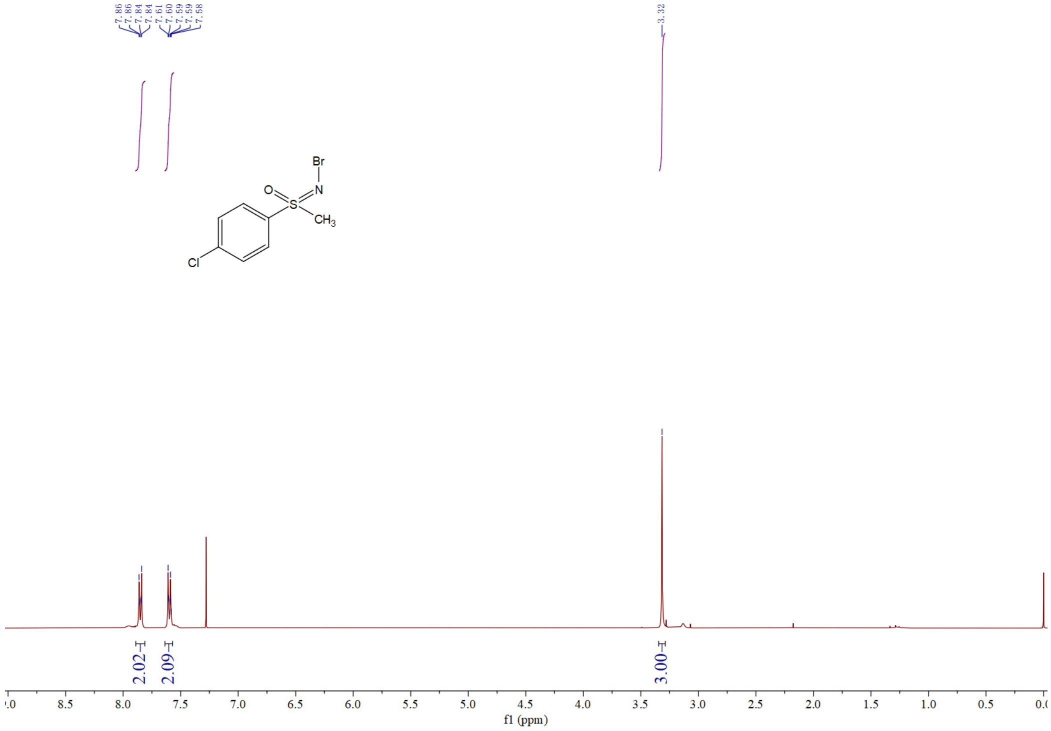
7.86
7.86
7.84
7.84
7.61
7.60
7.59
7.59
7.58
3.32
Br
O
S
N
CH3
Cl
2.02
2.09
3.00
f1 (ppm)

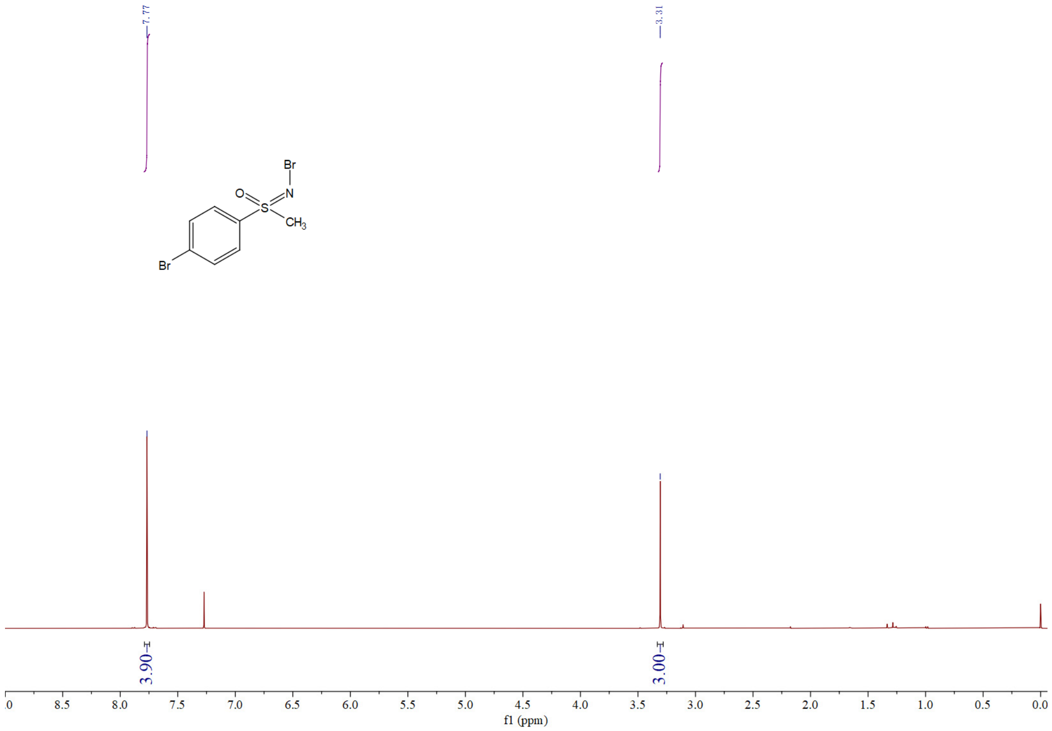
7.77
3.31
Br
N
O
S
CH3
Br
3.90
3.00
f1 (ppm)

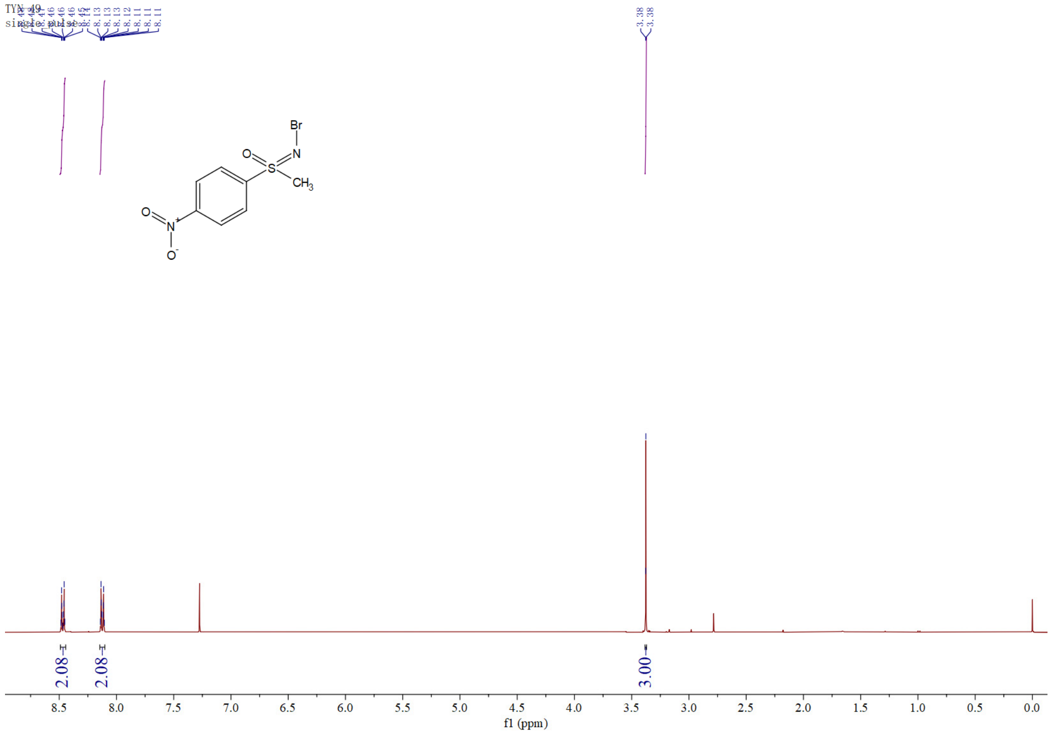
TYN-49
single_pulse
3.38
3.38
Br
N
O
S
CH3
N+
O
O-
2.08
2.08
3.00
f1 (ppm)

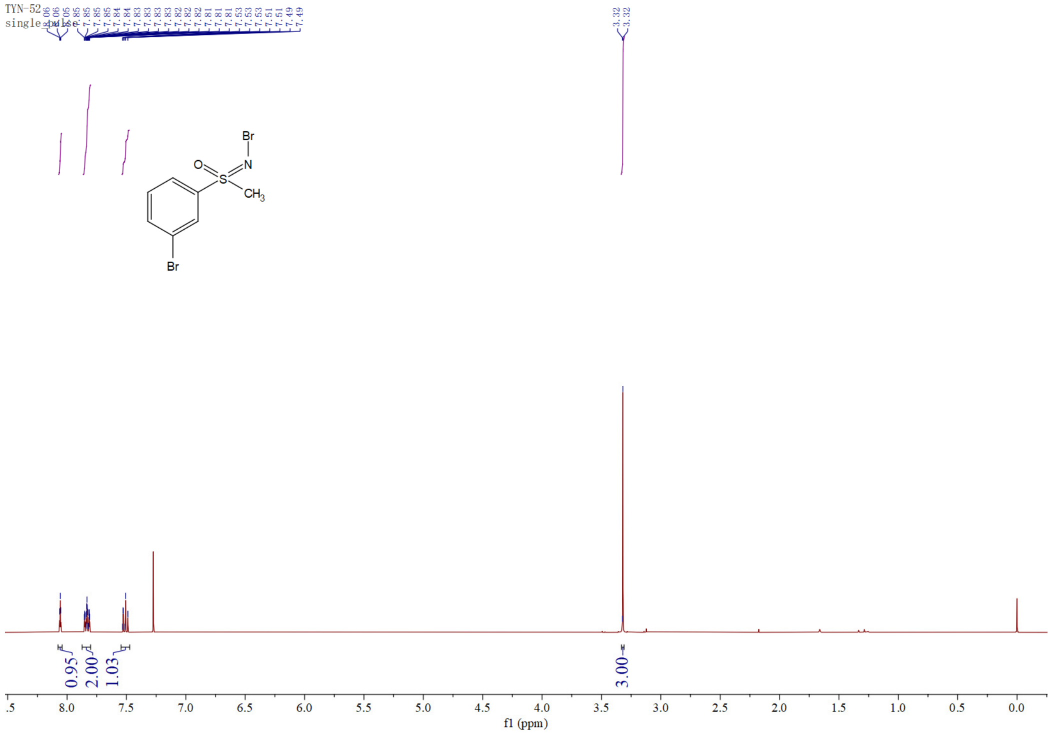

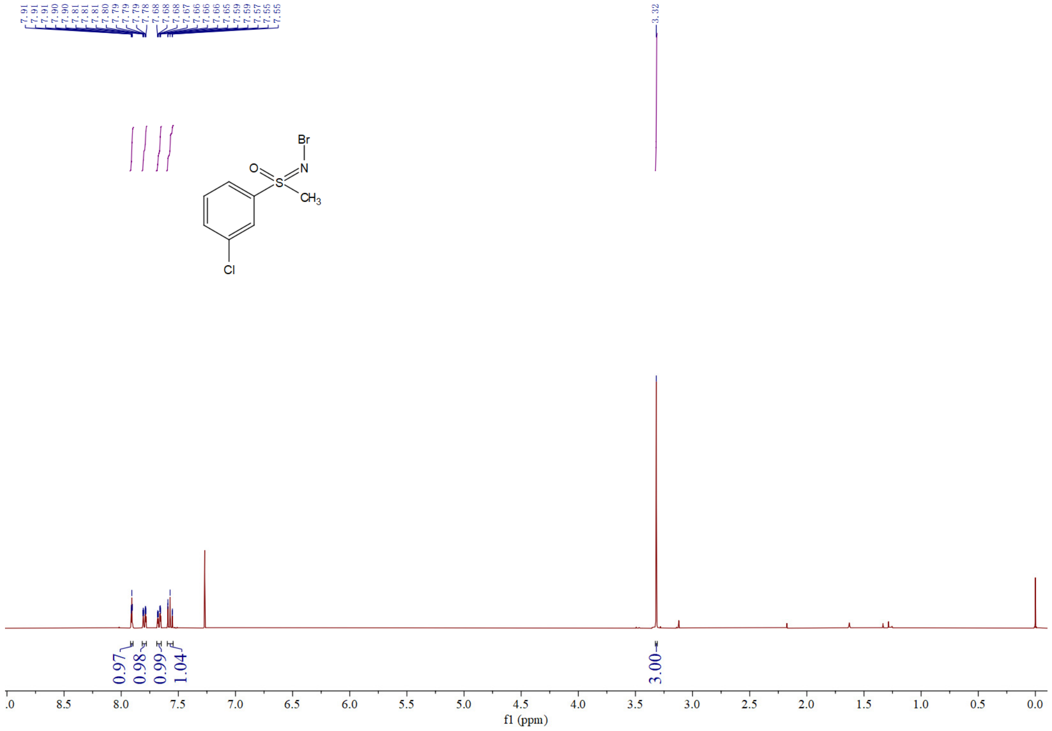

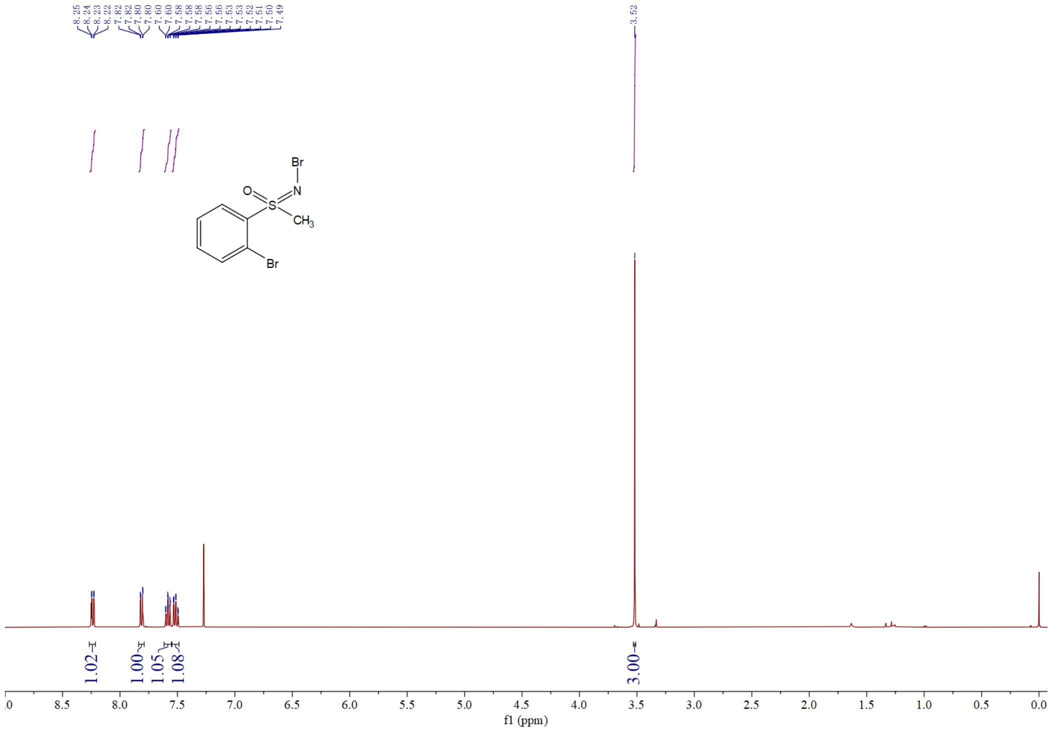

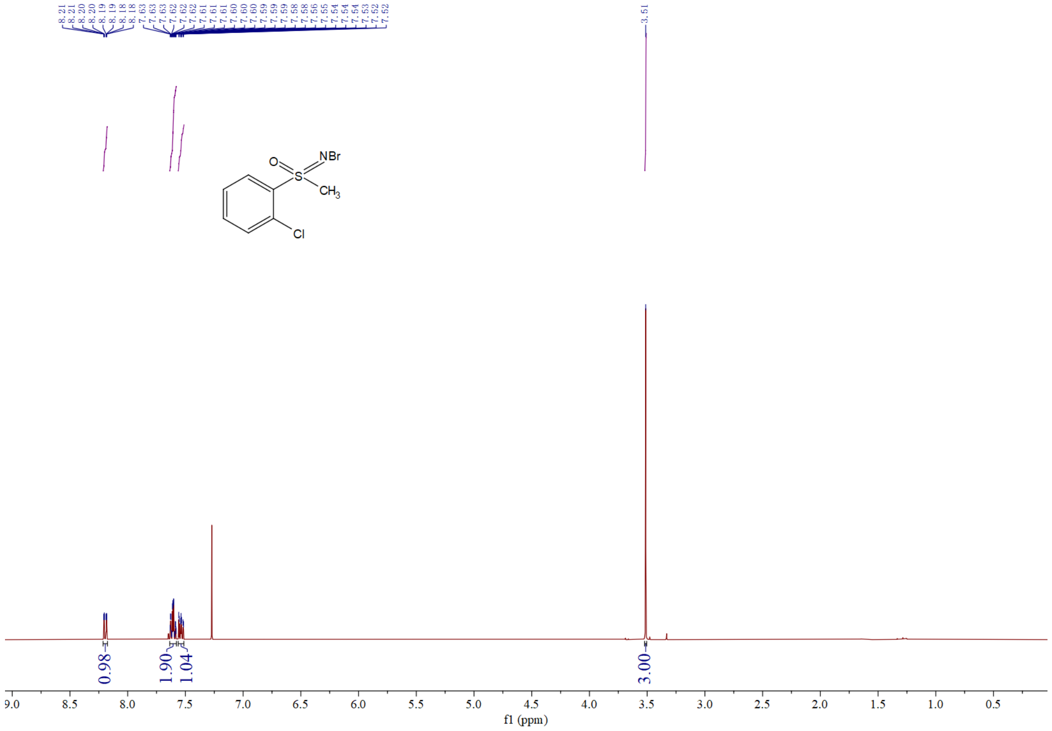

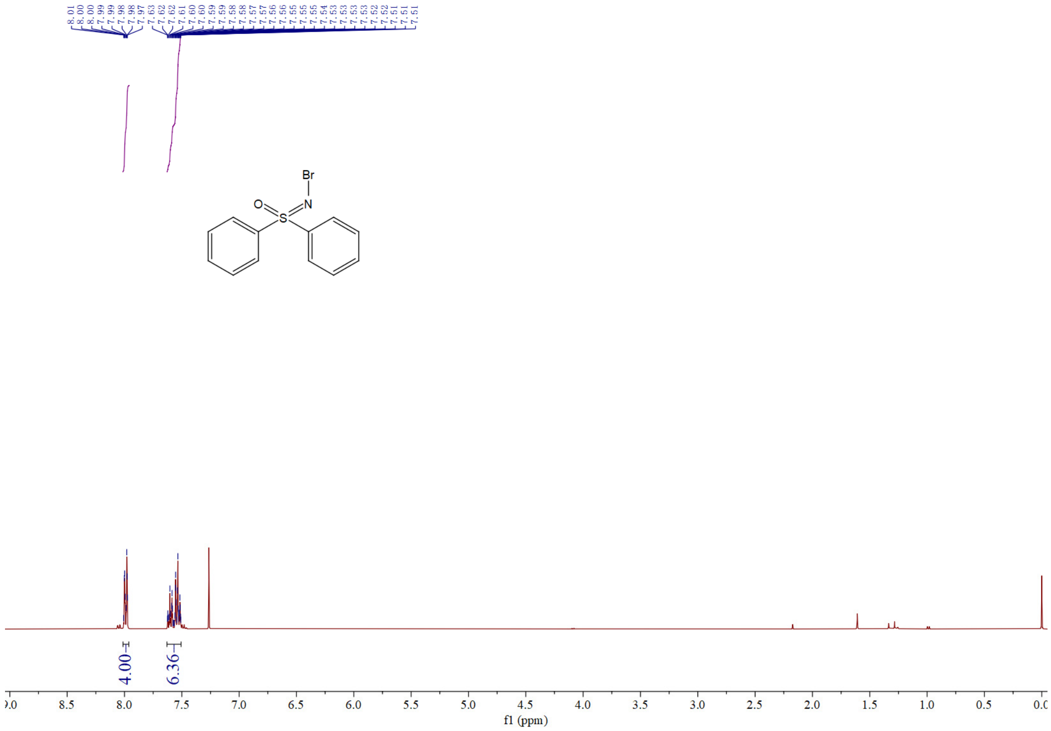
Br
O
S
N
4.00
6.36
f1 (ppm)

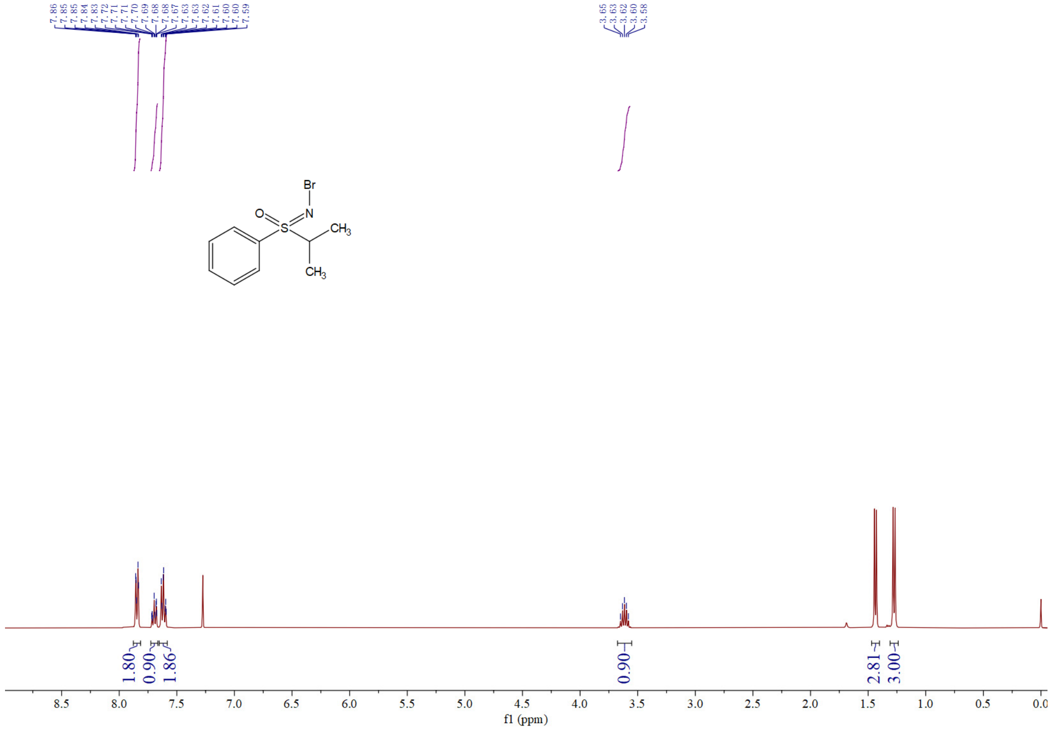
Br
O
S
N
CH_3
CH_3
1.80
0.90
1.86
0.90
2.81
3.00
f1 (ppm)

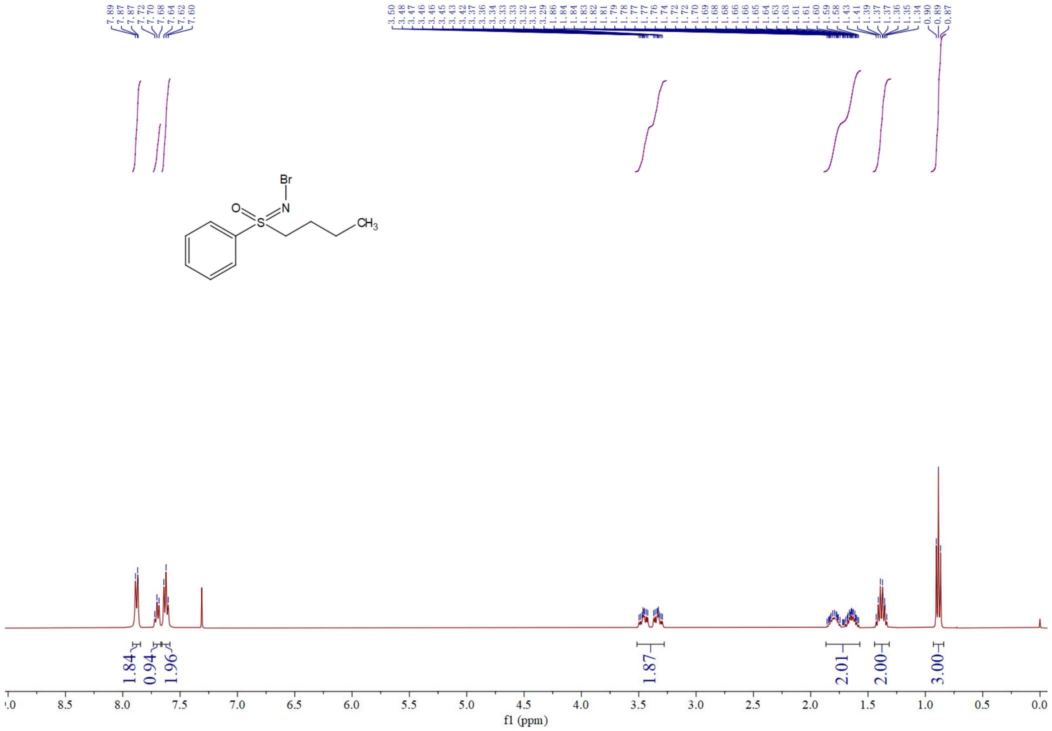
Br
N
O
S
CH3
1.84
0.94
1.96
1.87
2.01
2.00
3.00
f1 (ppm)

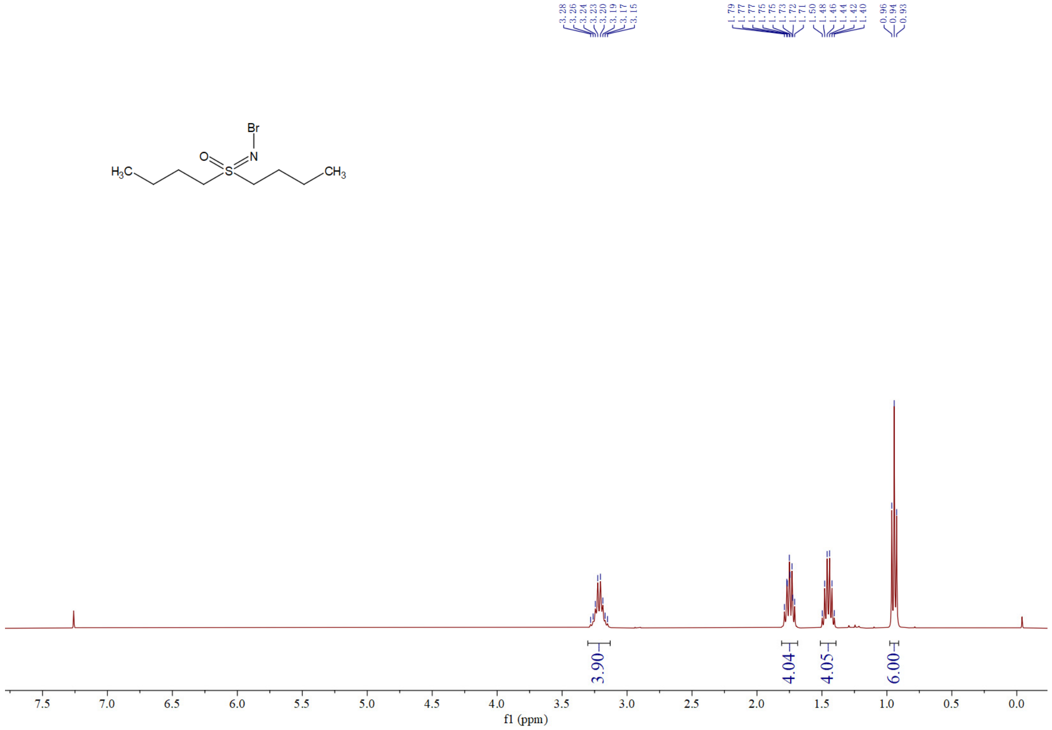
Br
N
O
S
H3C
CH3
3.90
4.04
4.05
6.00
f1 (ppm)

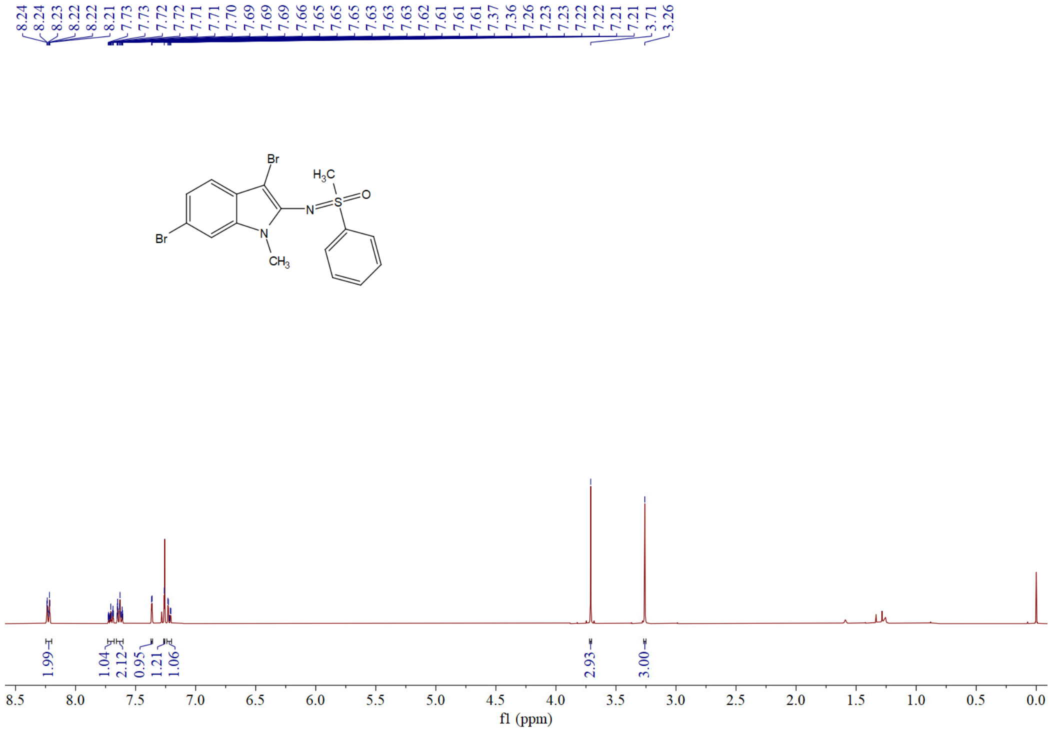

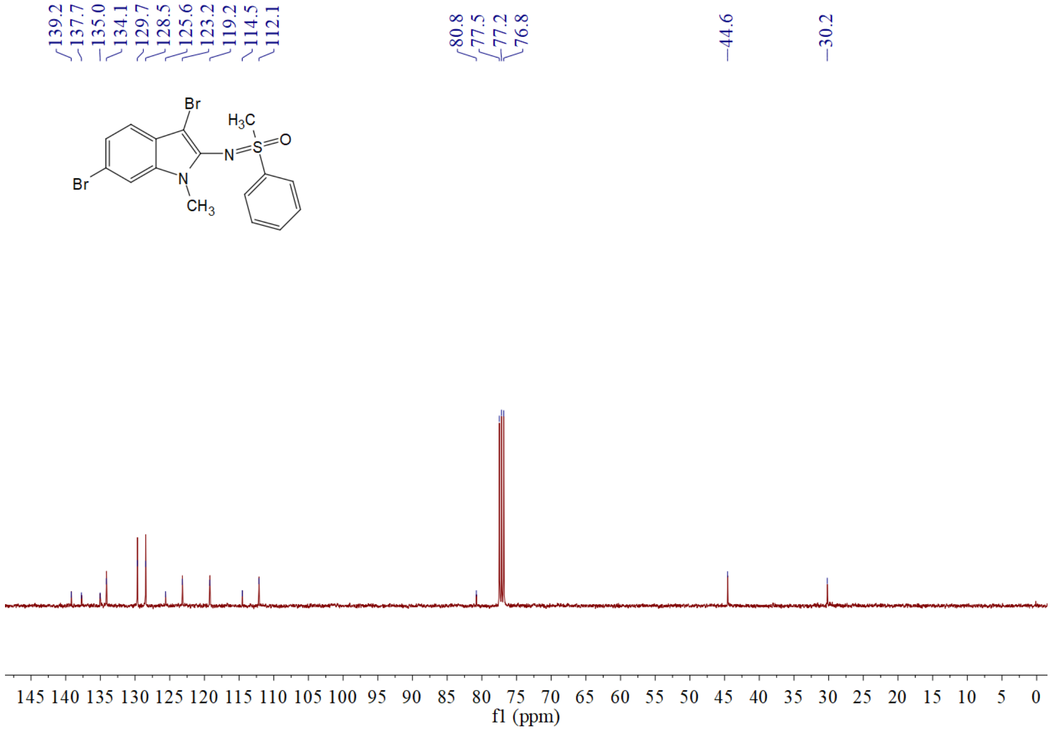

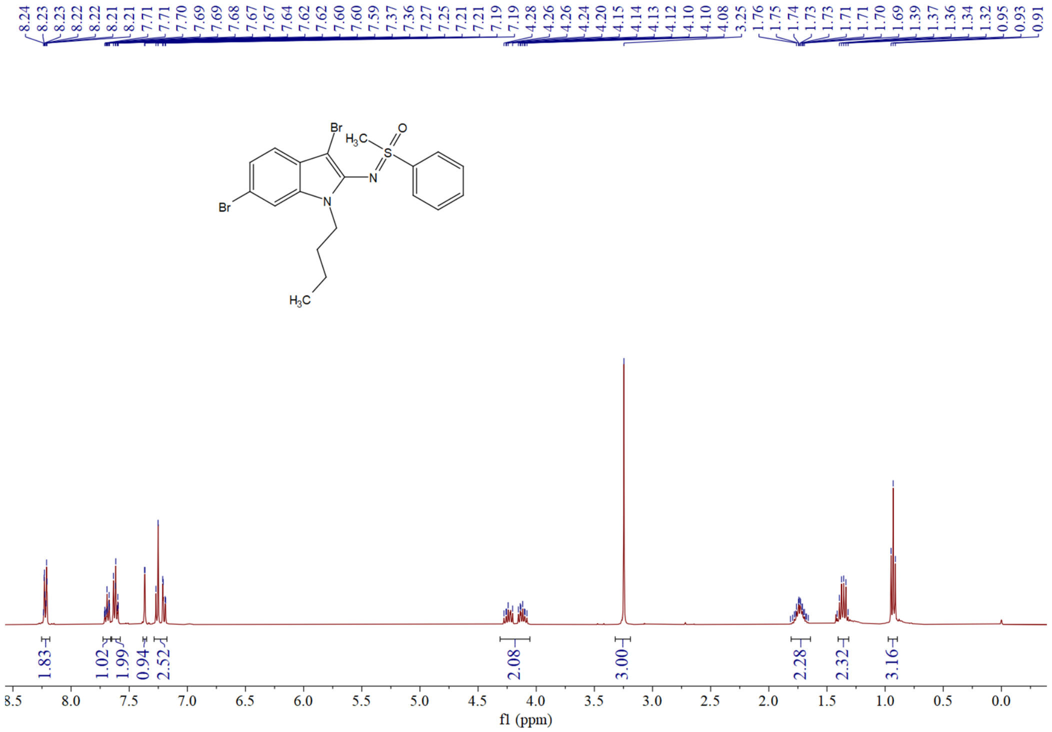
8.24 8.23 8.23 8.22 8.22 8.21 8.21 7.71 7.71 7.70 7.69 7.69 7.68 7.67 7.67 7.64 7.62 7.62 7.60 7.60 7.59 7.37 7.36 7.27 7.25 7.21 7.21 7.19 7.19 4.28 4.26 4.26 4.24 4.20 4.15 4.14 4.13 4.12 4.10 4.10 4.08 3.25 1.76 1.75 1.74 1.73 1.73 1.71 1.71 1.70 1.69 1.39 1.37 1.36 1.34 1.32 0.95 0.93 0.91
Br
H3C
O
S
N
Br
N
H3C
1.83
1.02
1.99
0.94
2.52
2.08
3.00
2.28
2.32
3.16
8.5 8.0 7.5 7.0 6.5 6.0 5.5 5.0 4.5 4.0 3.5 3.0 2.5 2.0 1.5 1.0 0.5 0.0
f1 (ppm)

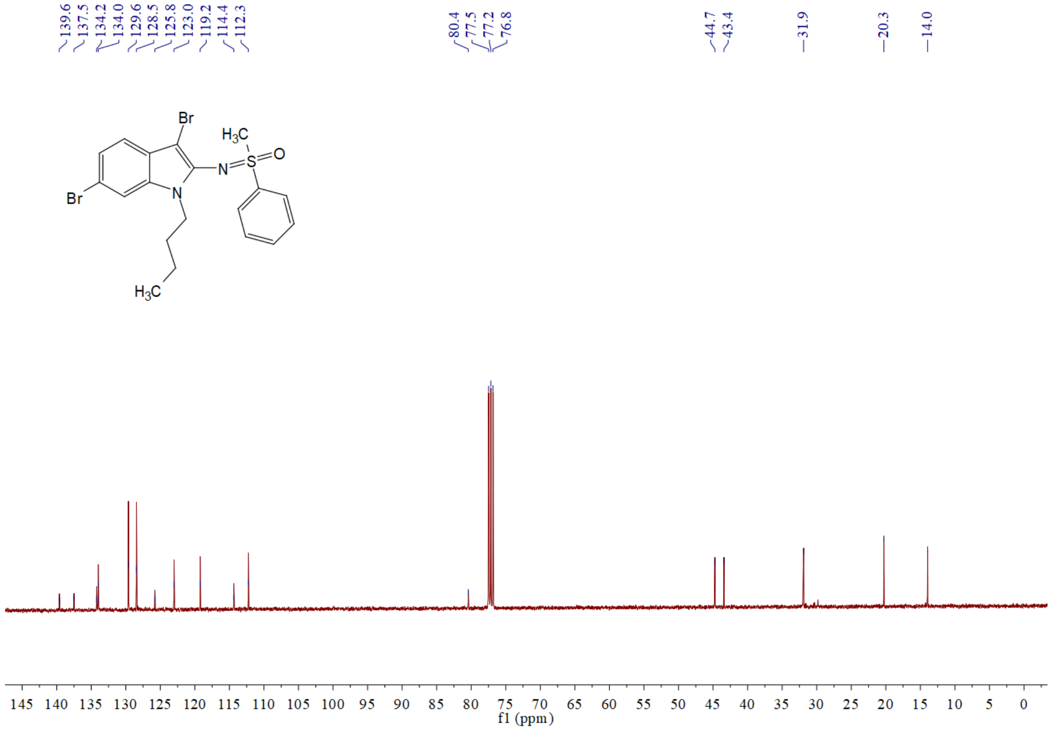
139.6 137.5 134.2 134.0 129.6 128.5 125.8 123.0 119.2 114.4 112.3
80.4 77.5 77.2 76.8
44.7 43.4
31.9
20.3
14.0
Br
H3C
N
S
O
Br
N
H3C
145 140 135 130 125 120 115 110 105 100 95 90 85 80 75 70 65 60 55 50 45 40 35 30 25 20 15 10 5 0
f1 (ppm)

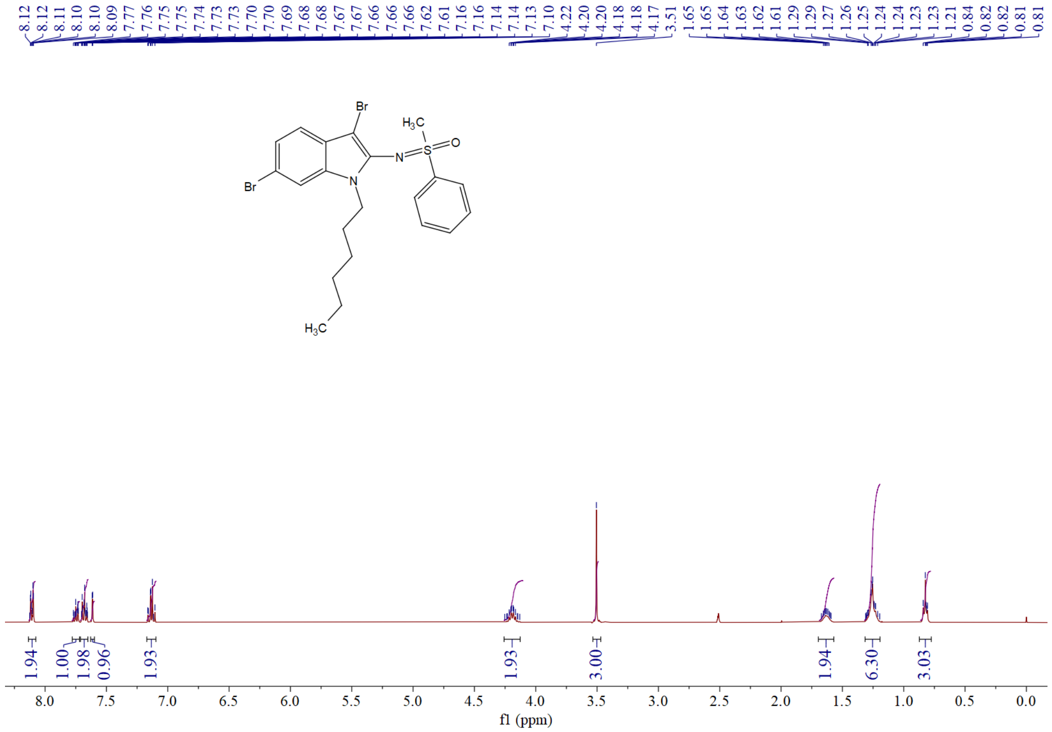

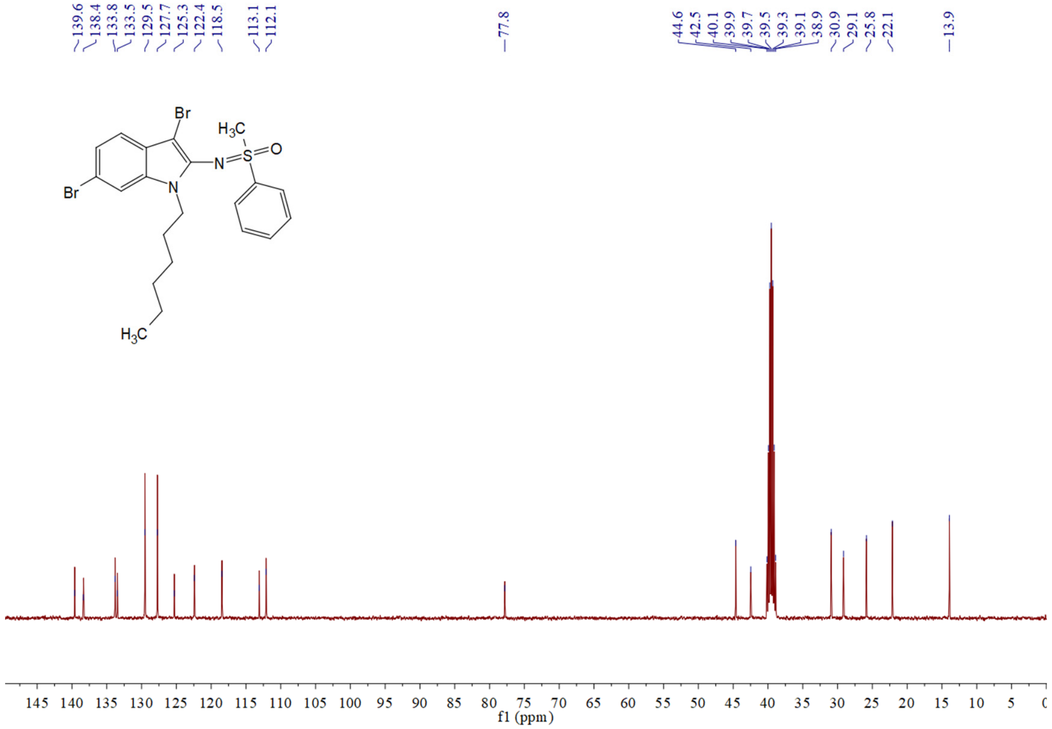

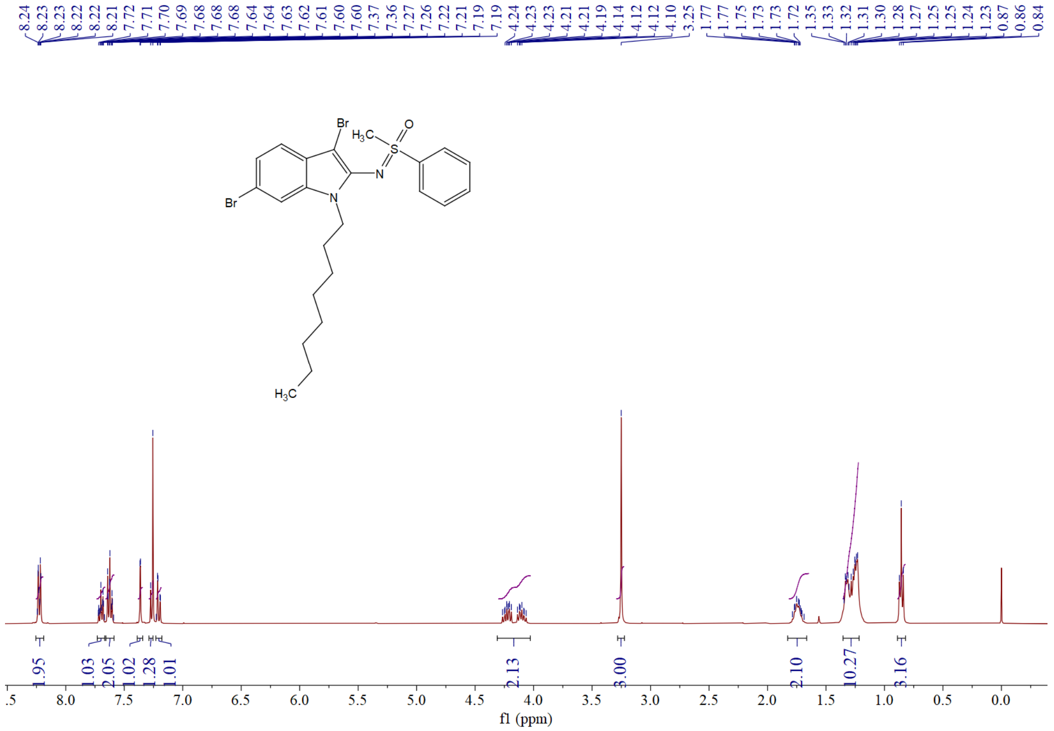
8.24 8.23 8.23 8.22 8.22 8.21 7.72 7.71 7.70 7.69 7.68 7.68 7.68 7.64 7.64 7.63 7.62 7.61 7.60 7.60 7.37 7.36 7.27 7.26 7.22 7.21 7.19 7.19 4.24 4.23 4.23 4.21 4.21 4.19 4.14 4.12 4.12 4.10 3.25 1.77 1.77 1.75 1.73 1.73 1.72 1.35 1.33 1.32 1.31 1.30 1.28 1.27 1.25 1.25 1.24 1.23 0.87 0.86 0.84
Br
H3C
O
S
N
N
Br
H3C
1.95 1.03 2.05 1.02 1.28 1.01 2.13 3.00 2.10 10.27 3.16
f1 (ppm)

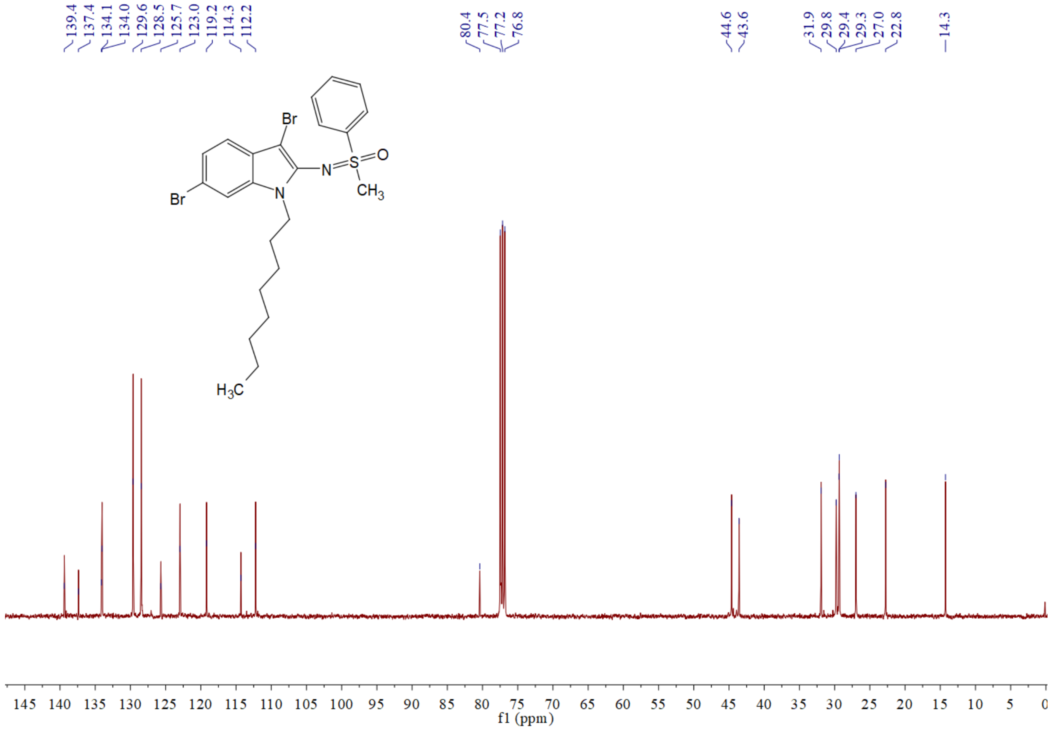
139.4 137.4 134.1 134.0 129.6 128.5 125.7 123.0 119.2 114.3 112.2 80.4 77.5 77.2 76.8 44.6 43.6 31.9 29.8 29.4 29.3 27.0 22.8 14.3
Br
N
S
O
CH3
Br
N
H3C
f1 (ppm)

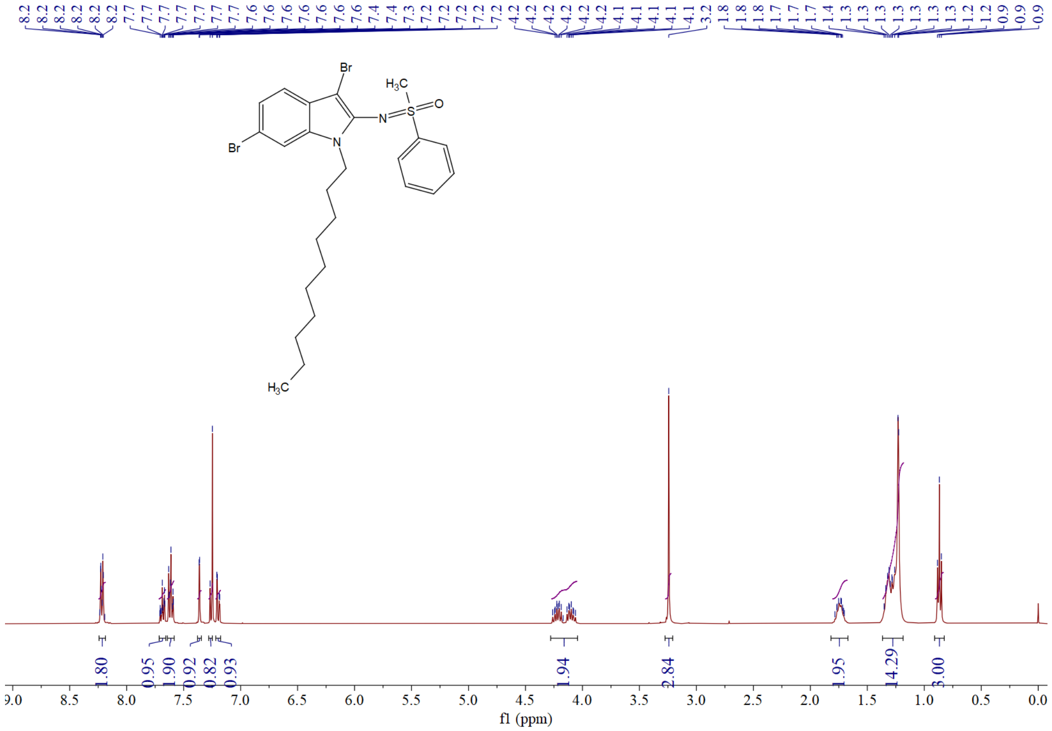

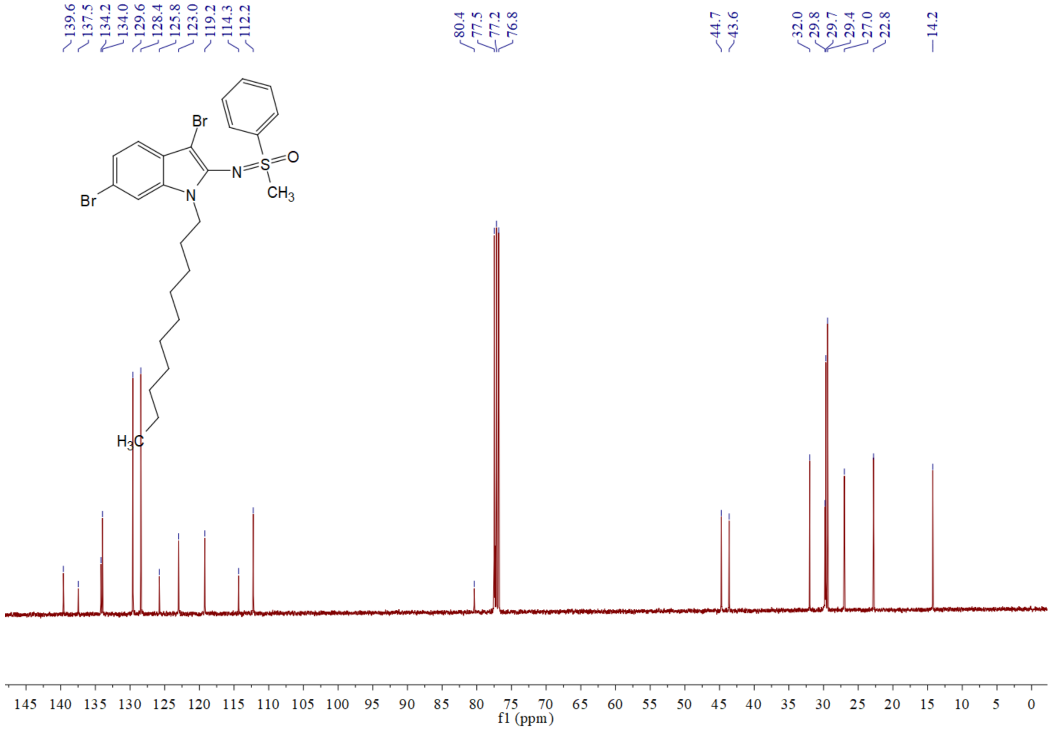

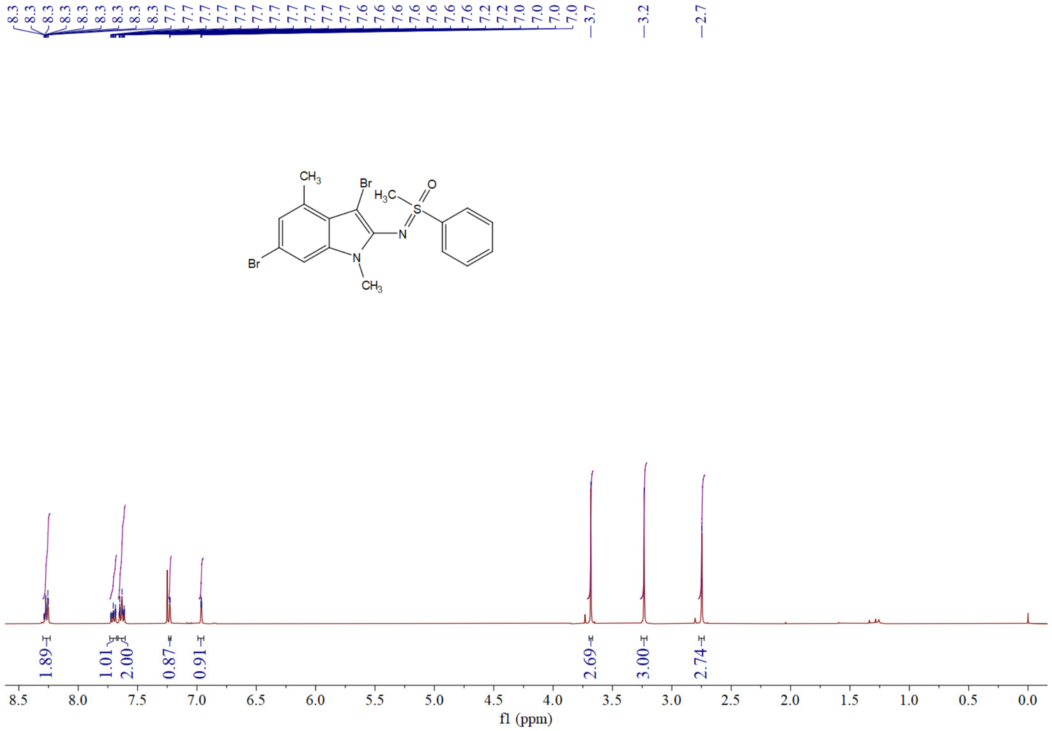

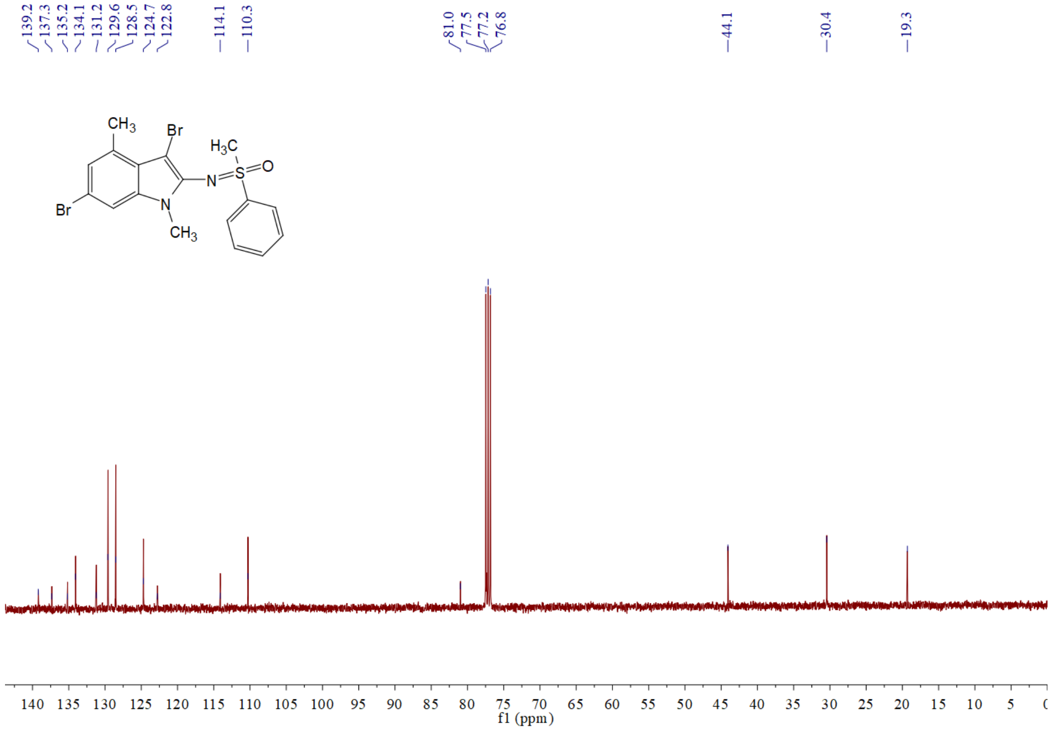

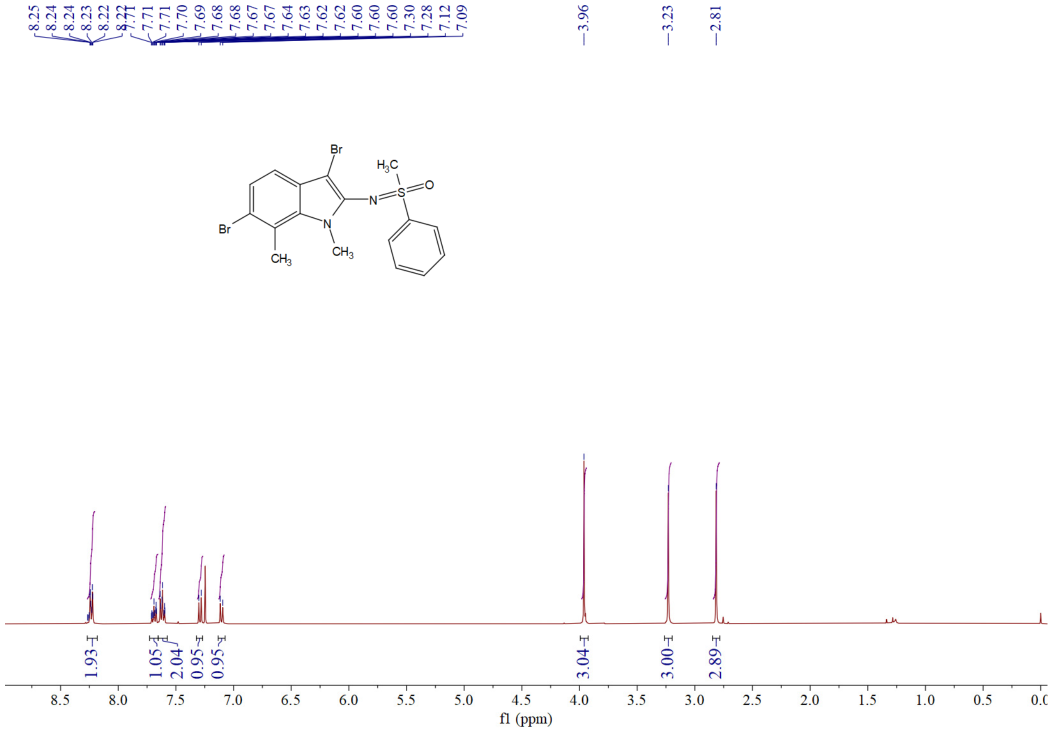
8.25
8.24
8.24
8.23
8.22
8.22
7.71
7.71
7.71
7.70
7.69
7.68
7.68
7.67
7.67
7.64
7.63
7.62
7.62
7.60
7.60
7.60
7.30
7.28
7.12
7.09
3.96
3.23
2.81
Br
H3C
N
S
O
Br
N
CH3
CH3
1.93
1.05
2.04
0.95
0.95
3.04
3.00
2.89
8.5 8.0 7.5 7.0 6.5 6.0 5.5 5.0 4.5 4.0 3.5 3.0 2.5 2.0 1.5 1.0 0.5 0.0
f1 (ppm)

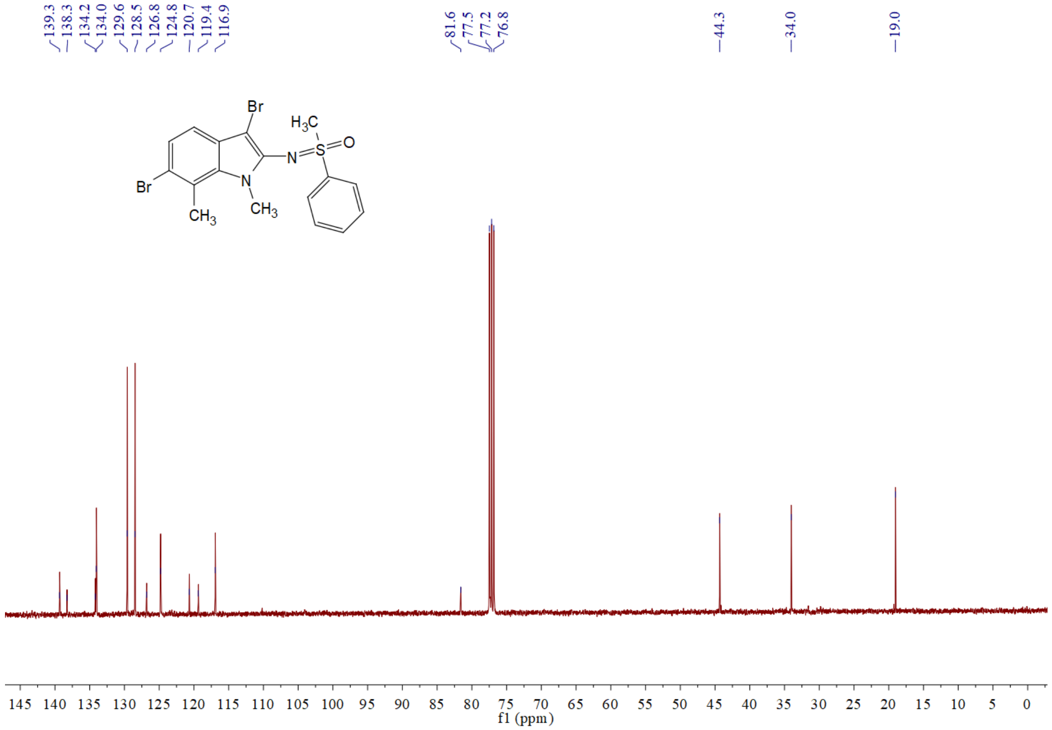
139.3
138.3
134.2
134.0
129.6
128.5
126.8
124.8
120.7
119.4
116.9
81.6
77.5
77.2
76.8
44.3
34.0
19.0
Br
H3C
N
S
O
Br
N
CH3
CH3
145 140 135 130 125 120 115 110 105 100 95 90 85 80 75 70 65 60 55 50 45 40 35 30 25 20 15 10 5 0
f1 (ppm)

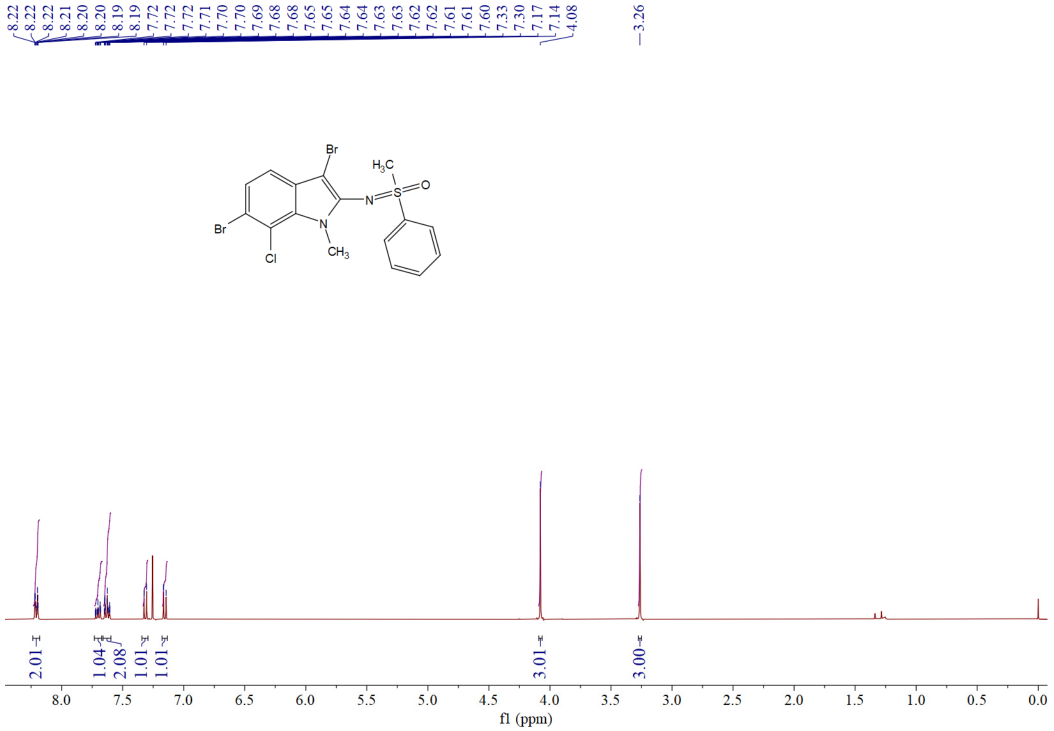
f1 (ppm)

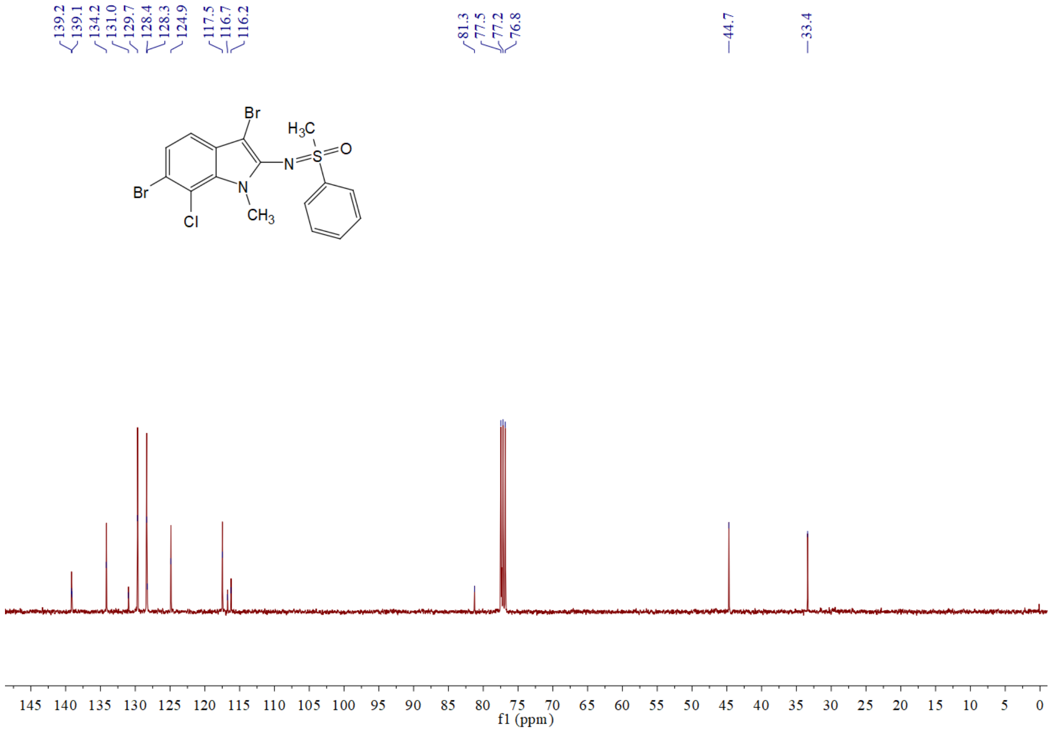
f1 (ppm)

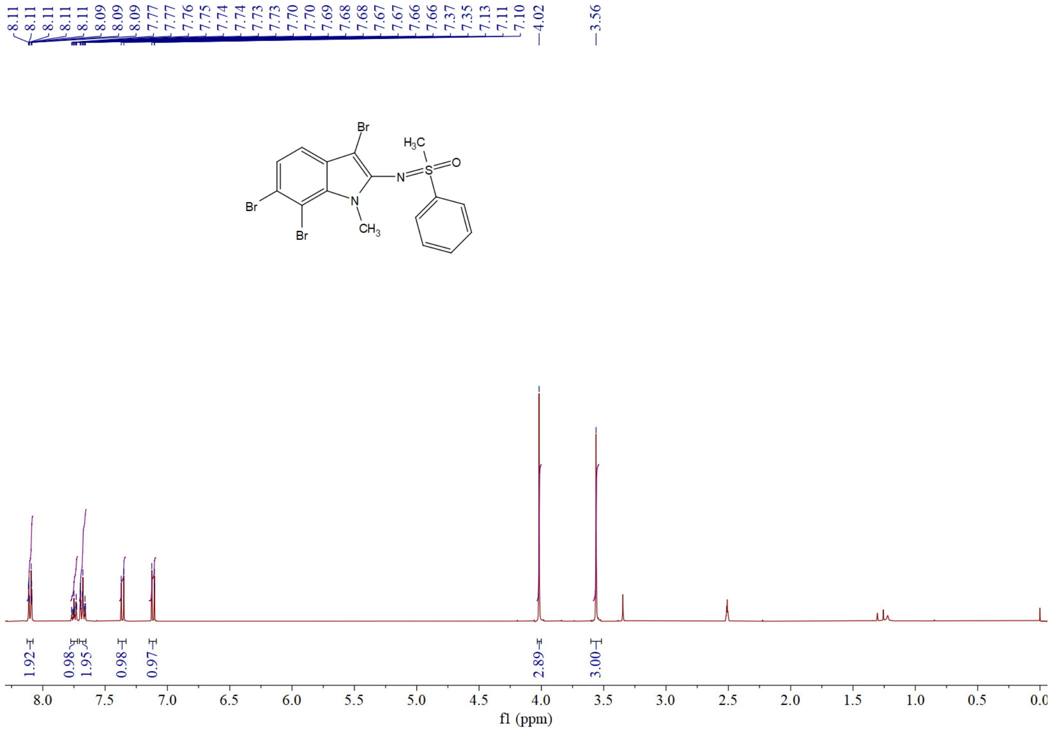
8.11
8.11
8.11
8.11
8.11
8.09
8.09
8.09
7.77
7.77
7.76
7.75
7.74
7.74
7.73
7.73
7.70
7.70
7.69
7.68
7.68
7.67
7.67
7.66
7.66
7.37
7.35
7.13
7.11
7.10
4.02
3.56
Br
H3C
N
S
O
Br
Br
N
CH3
1.92
0.98
1.95
0.98
0.97
2.89
3.00
f1 (ppm)

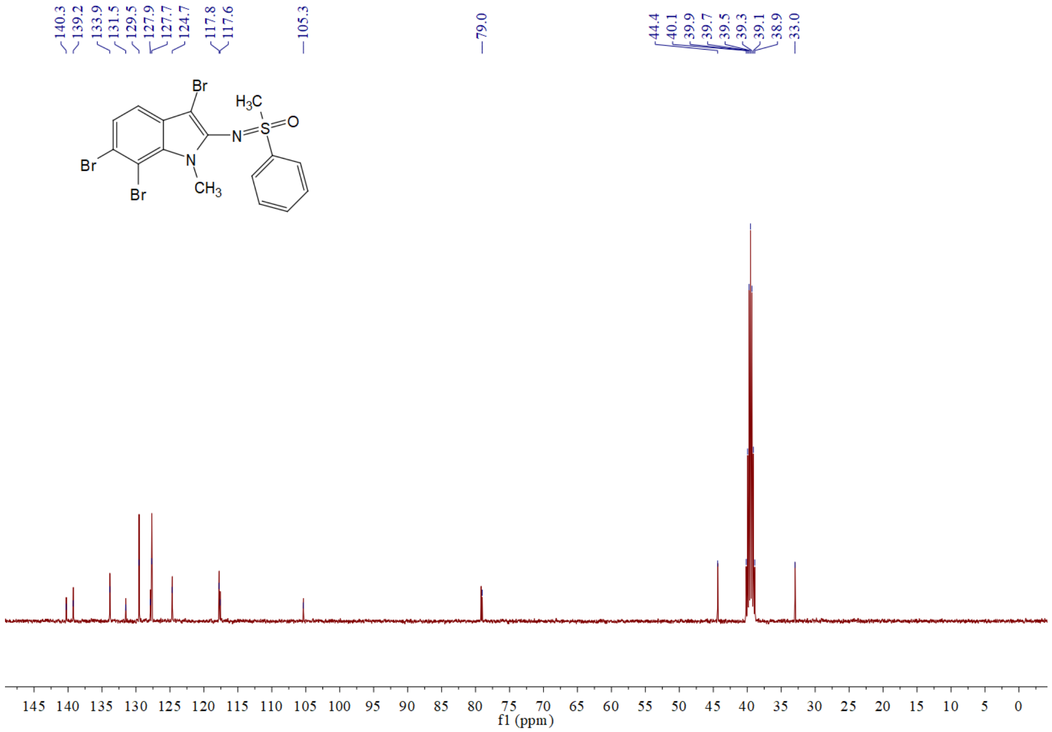
140.3
139.2
133.9
131.5
129.5
127.9
127.7
124.7
117.8
117.6
105.3
79.0
44.4
40.1
39.9
39.7
39.5
39.3
39.1
38.9
33.0
Br
H3C
N
S
O
Br
Br
N
CH3
f1 (ppm)

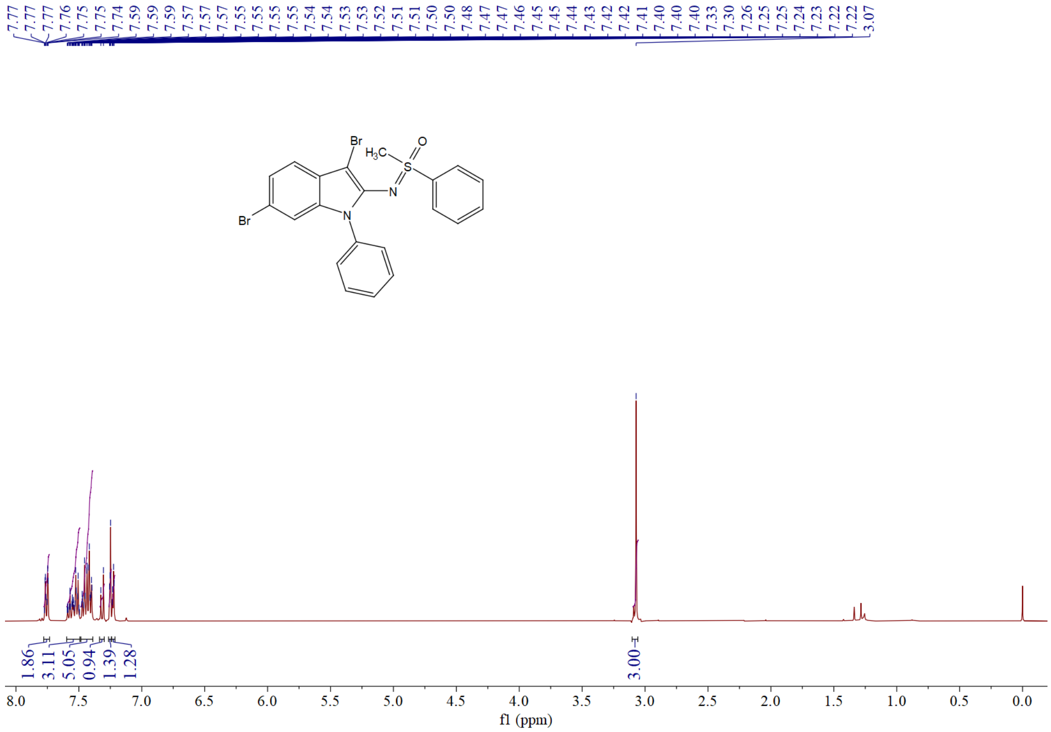
7.77 7.77 7.77 7.76 7.75 7.75 7.74 7.59 7.59 7.59 7.57 7.57 7.57 7.55 7.55 7.55 7.54 7.54 7.53 7.53 7.52 7.51 7.51 7.50 7.50 7.48 7.47 7.47 7.46 7.45 7.45 7.44 7.43 7.42 7.42 7.41 7.40 7.40 7.33 7.30 7.26 7.25 7.25 7.24 7.23 7.22 7.22 3.07
Br
H3C
O
S
N
Br
N
1.86 3.11 5.05 0.94 1.39 1.28
3.00
f1 (ppm)

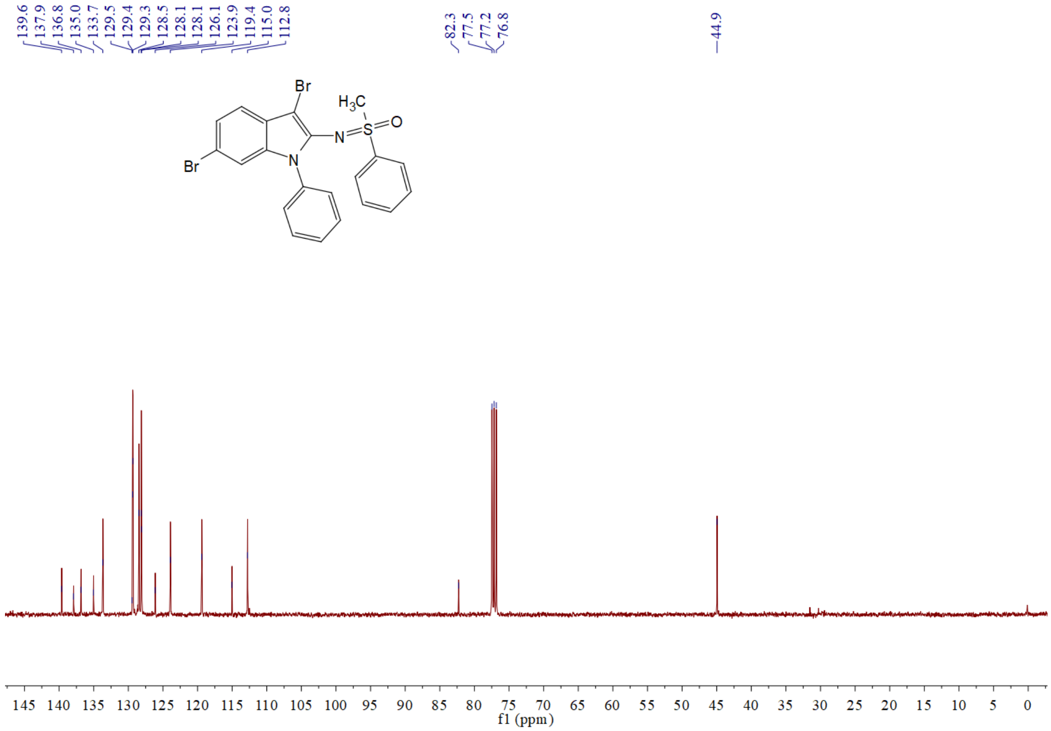
139.6 137.9 136.8 135.0 133.7 129.5 129.4 129.3 128.5 128.1 128.1 126.1 123.9 119.4 115.0 112.8
82.3 77.5 77.2 76.8
44.9
Br
H3C
N
S
O
Br
N
f1 (ppm)

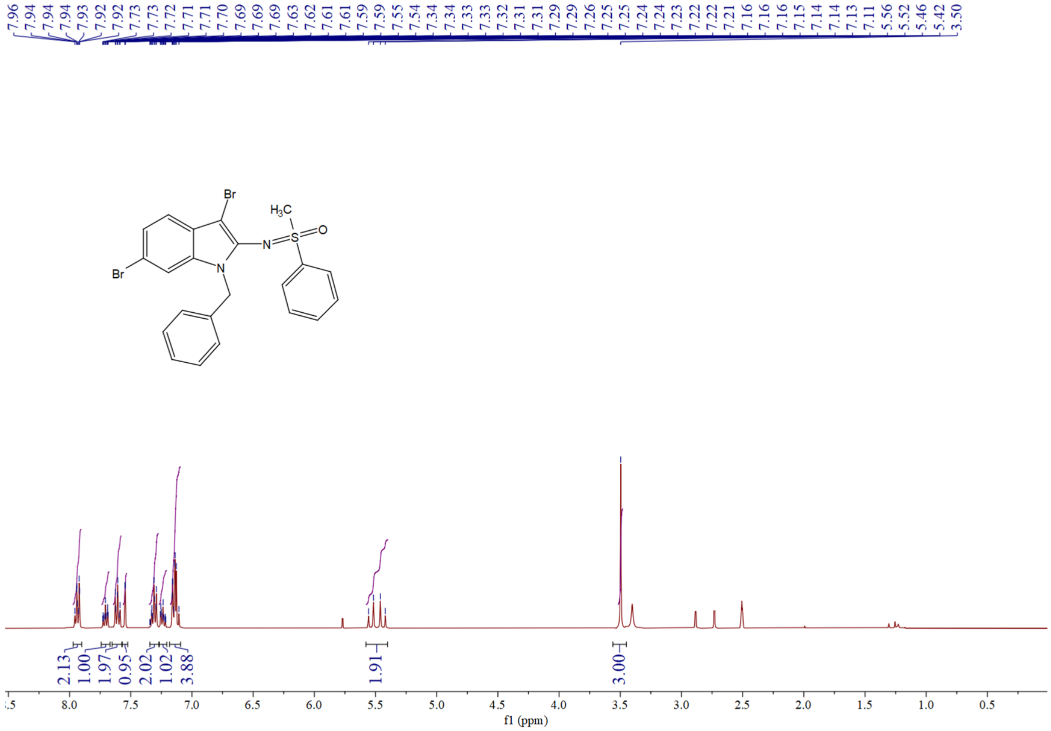
7.96 7.94 7.94 7.94 7.93 7.92 7.92 7.73 7.73 7.72 7.71 7.71 7.70 7.69 7.69 7.69 7.63 7.62 7.61 7.61 7.59 7.59 7.55 7.54 7.34 7.34 7.33 7.33 7.32 7.31 7.31 7.29 7.29 7.26 7.25 7.25 7.24 7.24 7.23 7.22 7.22 7.21 7.16 7.16 7.16 7.15 7.14 7.14 7.13 7.11 5.56 5.52 5.46 5.42 3.50
2.13 1.00 1.97 0.95 2.02 1.02 3.88 1.91 3.00
f1 (ppm)

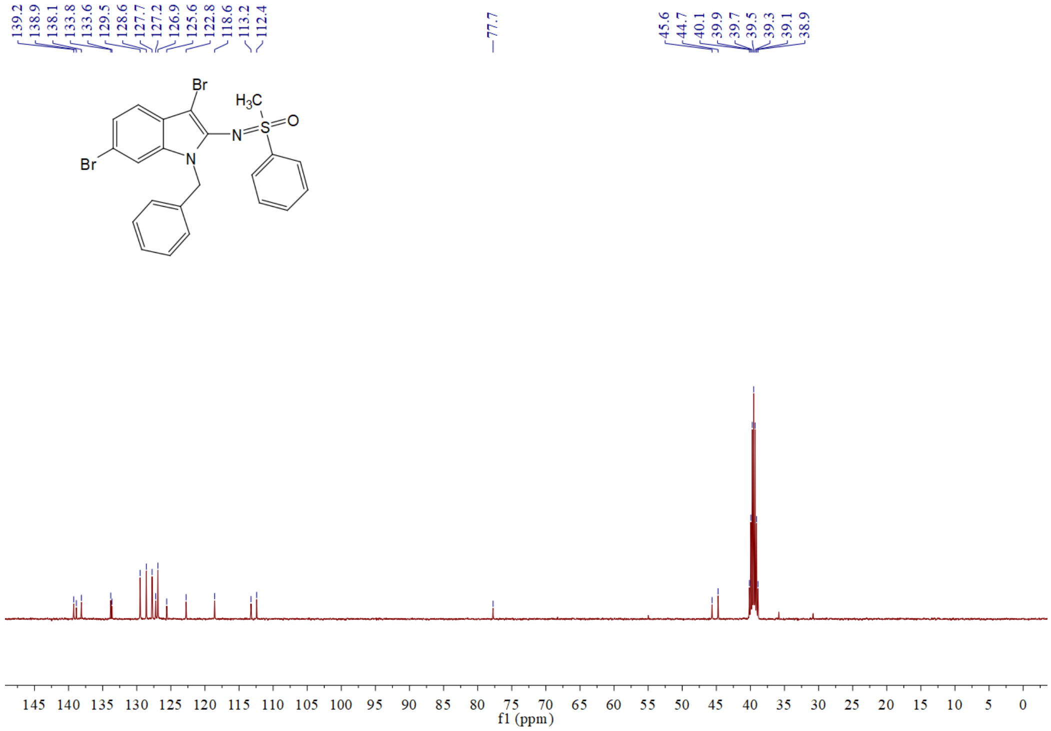
139.2 138.9 138.1 133.8 133.6 129.5 128.6 127.7 127.2 126.9 125.6 122.8 118.6 113.2 112.4 77.7 45.6 44.7 40.1 39.9 39.7 39.5 39.3 39.1 38.9
f1 (ppm)

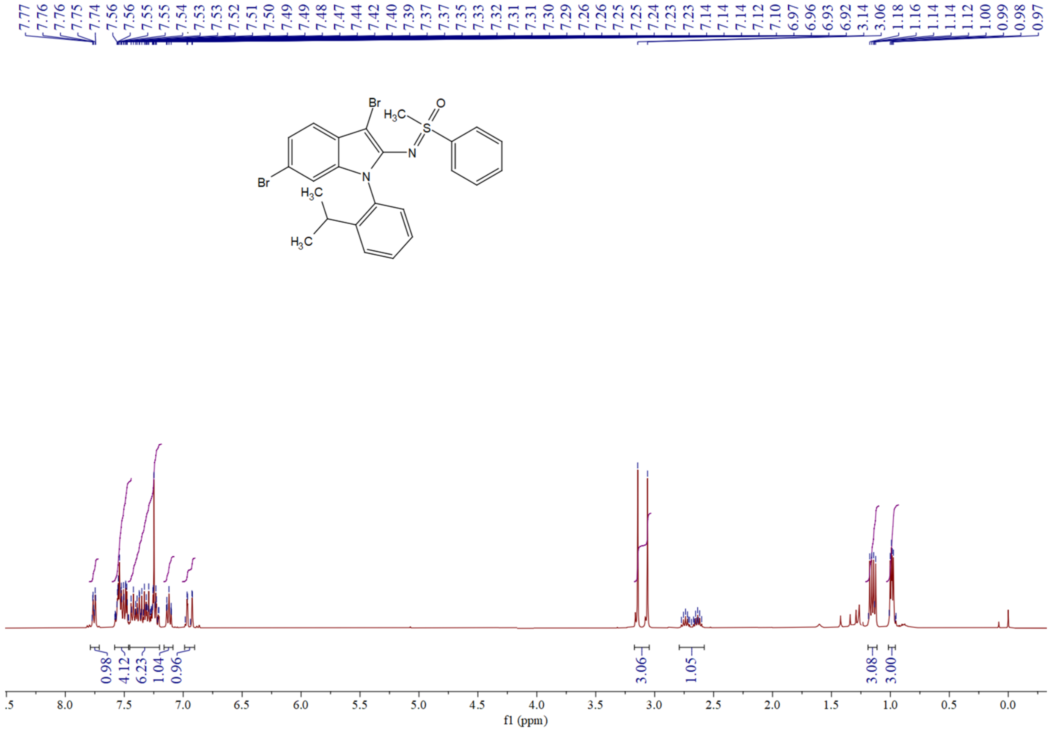
7.77 7.76 7.76 7.75 7.74 7.56 7.56 7.55 7.55 7.54 7.53 7.53 7.52 7.51 7.50 7.49 7.49 7.48 7.47 7.44 7.42 7.40 7.39 7.37 7.37 7.35 7.33 7.32 7.31 7.31 7.30 7.29 7.26 7.26 7.25 7.25 7.24 7.23 7.23 7.14 7.14 7.14 7.12 7.10 6.97 6.96 6.93 6.92 3.14 3.06 1.18 1.16 1.14 1.14 1.12 1.00 0.99 0.98 0.97
Br
Br
H3C
O
S
N
N
H3C
H3C
0.98
4.12
6.23
1.04
0.96
3.06
1.05
3.08
3.00
f1 (ppm)

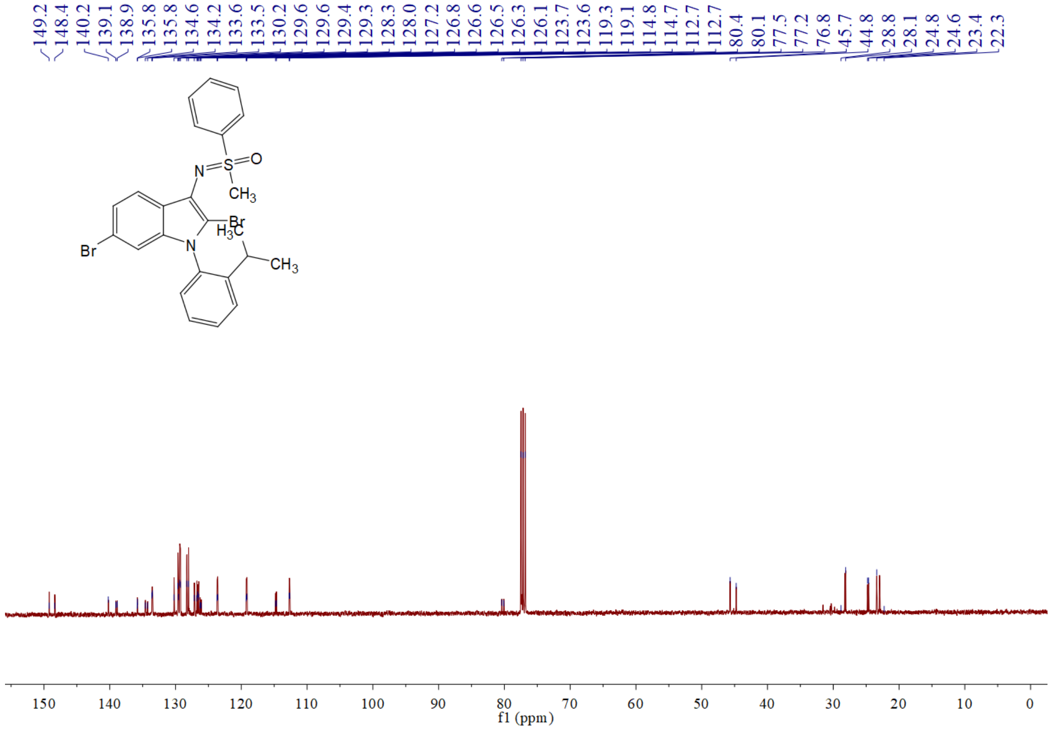
149.2 148.4 140.2 139.1 138.9 135.8 135.8 134.6 134.2 133.6 133.5 130.2 129.6 129.6 129.4 129.3 128.3 128.0 127.2 126.8 126.6 126.5 126.3 126.1 123.7 123.6 119.3 119.1 114.8 114.7 112.7 112.7 80.4 80.1 77.5 77.2 76.8 45.7 44.8 28.8 28.1 24.8 24.6 23.4 22.3
N
S
O
CH3
Br
H3C
CH3
Br
N
f1 (ppm)

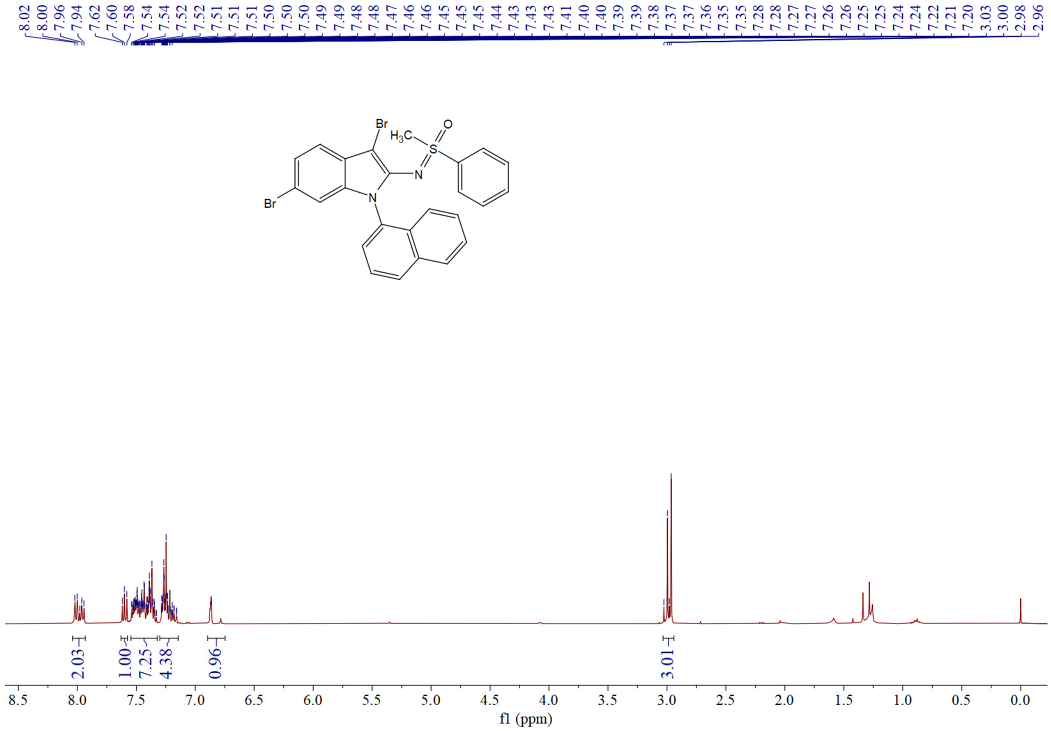
8.02 8.00 7.96 7.94 7.62 7.60 7.58 7.54 7.54 7.52 7.52 7.51 7.51 7.50 7.50 7.49 7.49 7.48 7.48 7.47 7.46 7.46 7.45 7.45 7.44 7.43 7.43 7.41 7.40 7.40 7.39 7.39 7.38 7.37 7.37 7.36 7.35 7.35 7.28 7.28 7.27 7.27 7.26 7.26 7.25 7.25 7.24 7.24 7.22 7.21 7.20 3.03 3.00 2.98 2.96
Br
H_3C
S
O
N
Br
N
2.03
1.00
7.25
4.38
0.96
3.01
8.5 8.0 7.5 7.0 6.5 6.0 5.5 5.0 4.5 4.0 3.5 3.0 2.5 2.0 1.5 1.0 0.5 0.0
f1 (ppm)

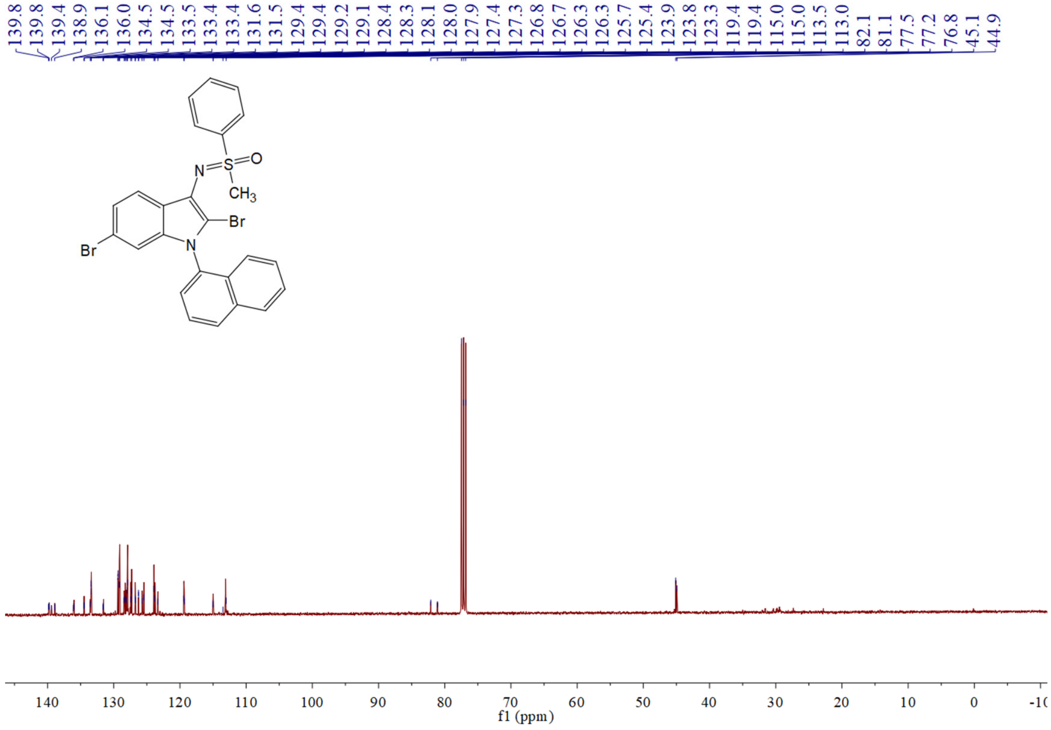
139.8 139.8 139.4 138.9 136.1 136.0 134.5 134.5 133.5 133.4 133.4 131.6 131.5 129.4 129.4 129.2 129.1 128.4 128.3 128.1 128.0 127.9 127.4 127.3 126.8 126.7 126.3 126.3 125.7 125.4 123.9 123.8 123.3 119.4 119.4 115.0 115.0 113.5 113.0 82.1 81.1 77.5 77.2 76.8 45.1 44.9
N
S
O
CH_3
Br
Br
N
140 130 120 110 100 90 80 70 60 50 40 30 20 10 0 -1(
f1 (ppm)

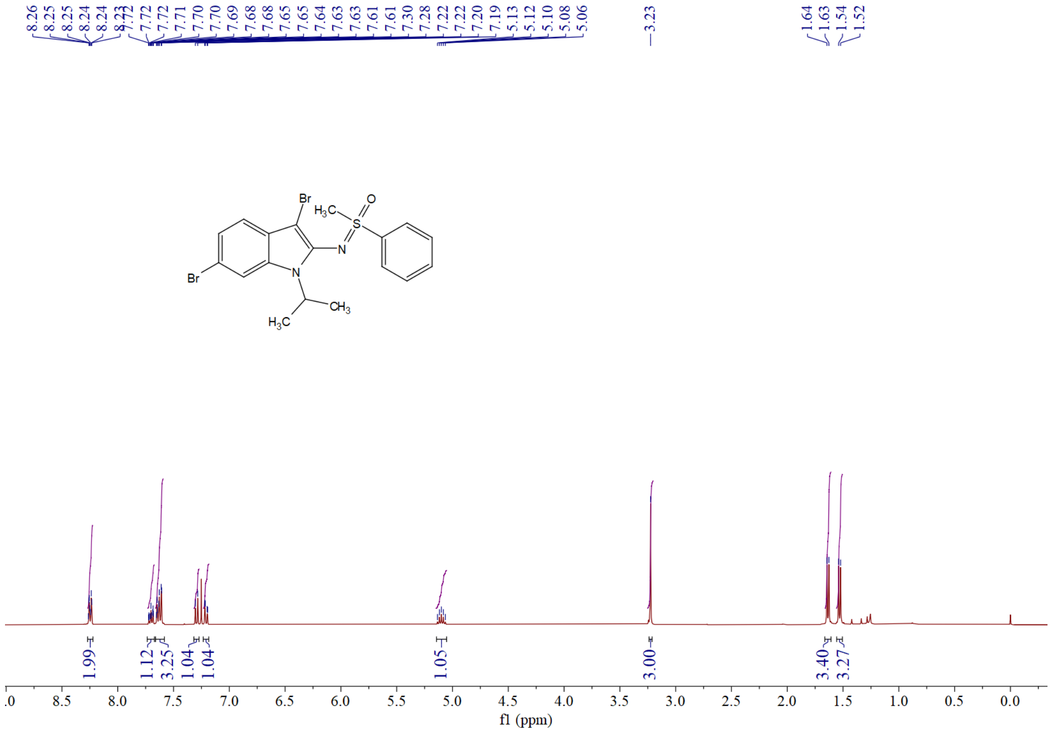
8.26
8.25
8.25
8.24
8.24
8.23
7.72
7.72
7.71
7.70
7.70
7.69
7.68
7.68
7.65
7.65
7.64
7.63
7.63
7.61
7.61
7.30
7.28
7.22
7.22
7.20
7.19
5.13
5.12
5.10
5.08
5.06
3.23
1.64
1.63
1.54
1.52
Br
H_3C
O
S
N
Br
N
CH_3
H_3C
1.99
1.12
3.25
1.04
1.04
1.05
3.00
3.40
3.27
f1 (ppm)

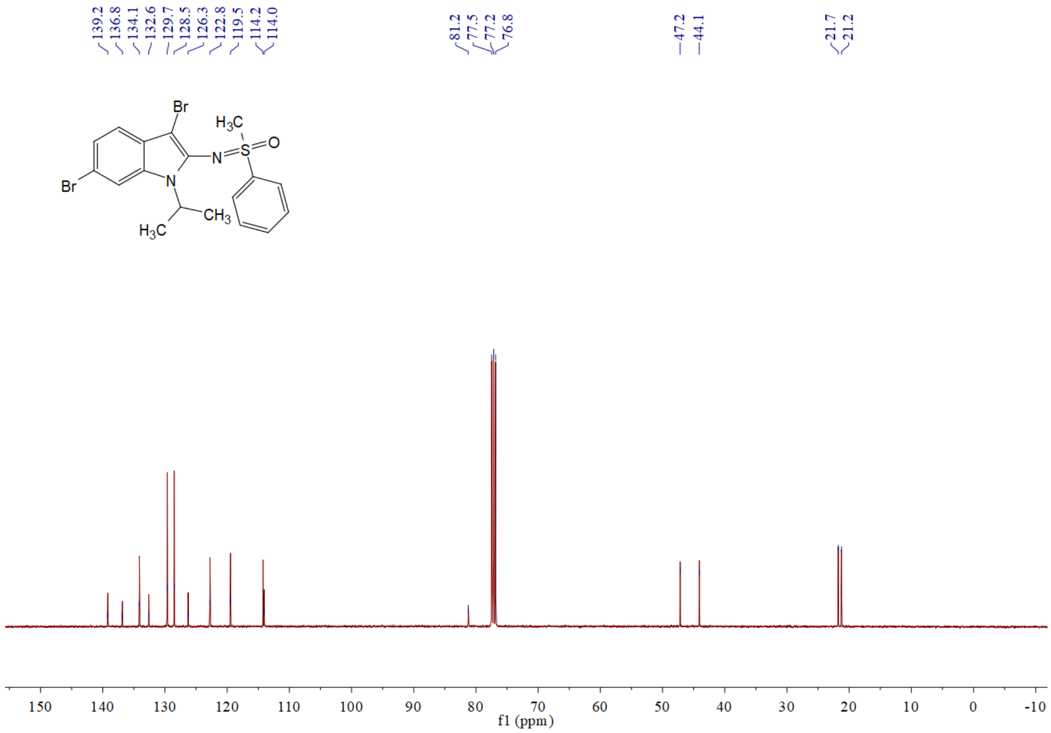
139.2
136.8
134.1
132.6
129.7
128.5
126.3
122.8
119.5
114.2
114.0
81.2
77.5
77.2
76.8
47.2
44.1
21.7
21.2
Br
H_3C
N
S
O
Br
N
CH_3
H_3C
f1 (ppm)

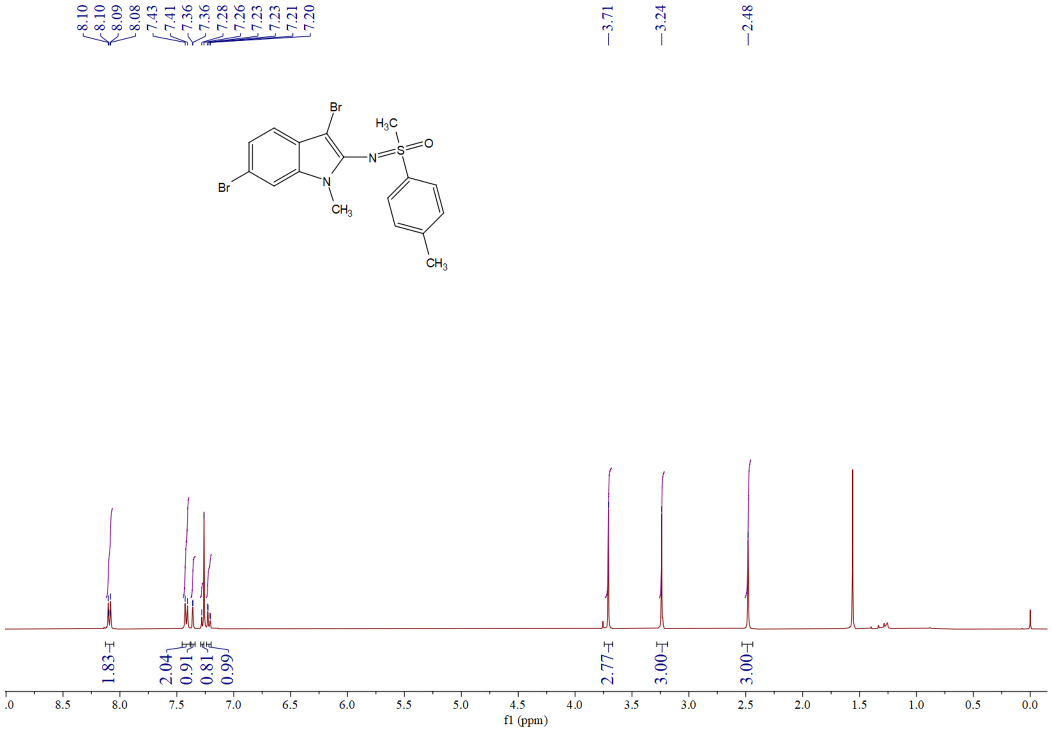
8.10
8.10
8.09
8.08
7.43
7.41
7.36
7.36
7.28
7.26
7.23
7.23
7.21
7.20
3.71
3.24
2.48
Br
Br
N
CH3
N
S
O
H3C
CH3
1.83
2.04
0.91
0.81
0.99
2.77
3.00
3.00
f1 (ppm)

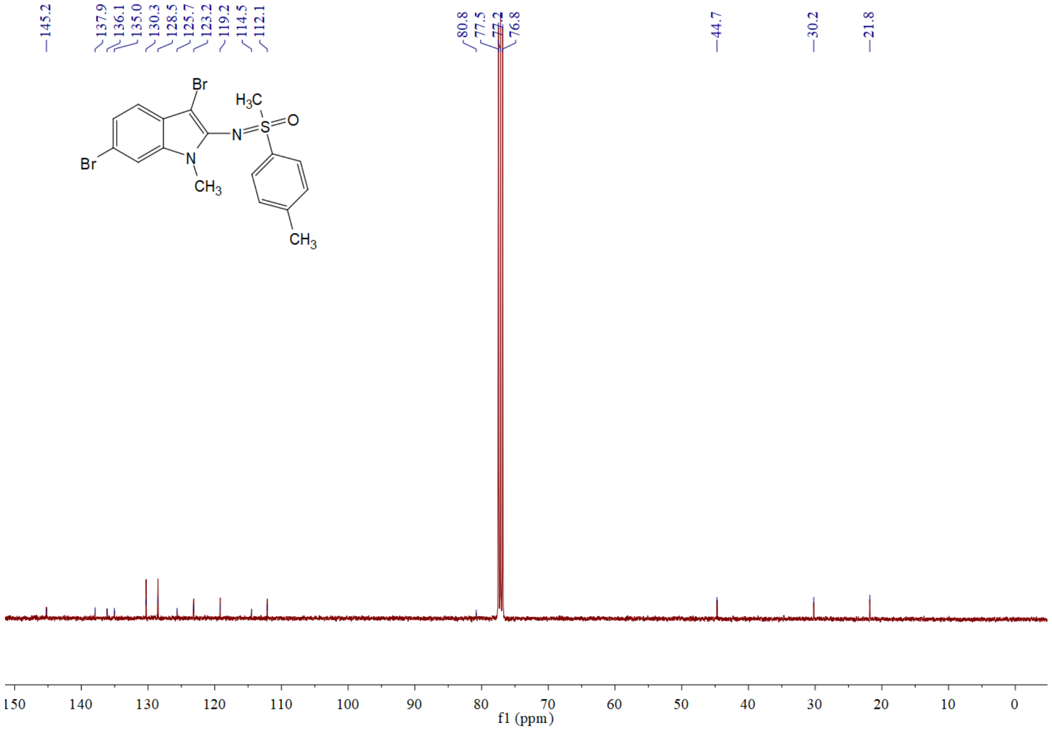
145.2
137.9
136.1
135.0
130.3
128.5
125.7
123.2
119.2
114.5
112.1
80.8
77.5
77.2
76.8
44.7
30.2
21.8
Br
Br
N
CH3
N
S
O
H3C
CH3
f1 (ppm)

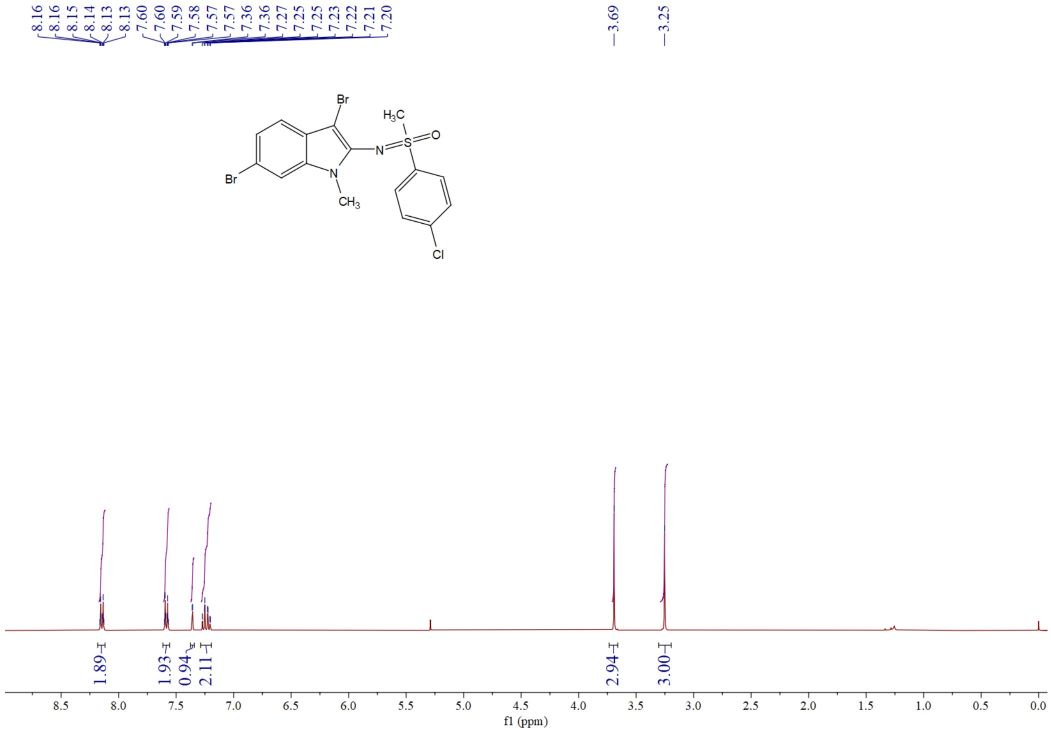
8.16
8.16
8.15
8.14
8.13
8.13
7.60
7.60
7.59
7.58
7.57
7.57
7.36
7.36
7.27
7.25
7.25
7.23
7.22
7.21
7.20
3.69
3.25
Br
H3C
N
S
O
Br
N
CH3
Cl
1.89
1.93
0.94
2.11
2.94
3.00
8.5 8.0 7.5 7.0 6.5 6.0 5.5 5.0 4.5 4.0 3.5 3.0 2.5 2.0 1.5 1.0 0.5 0.0
f1 (ppm)

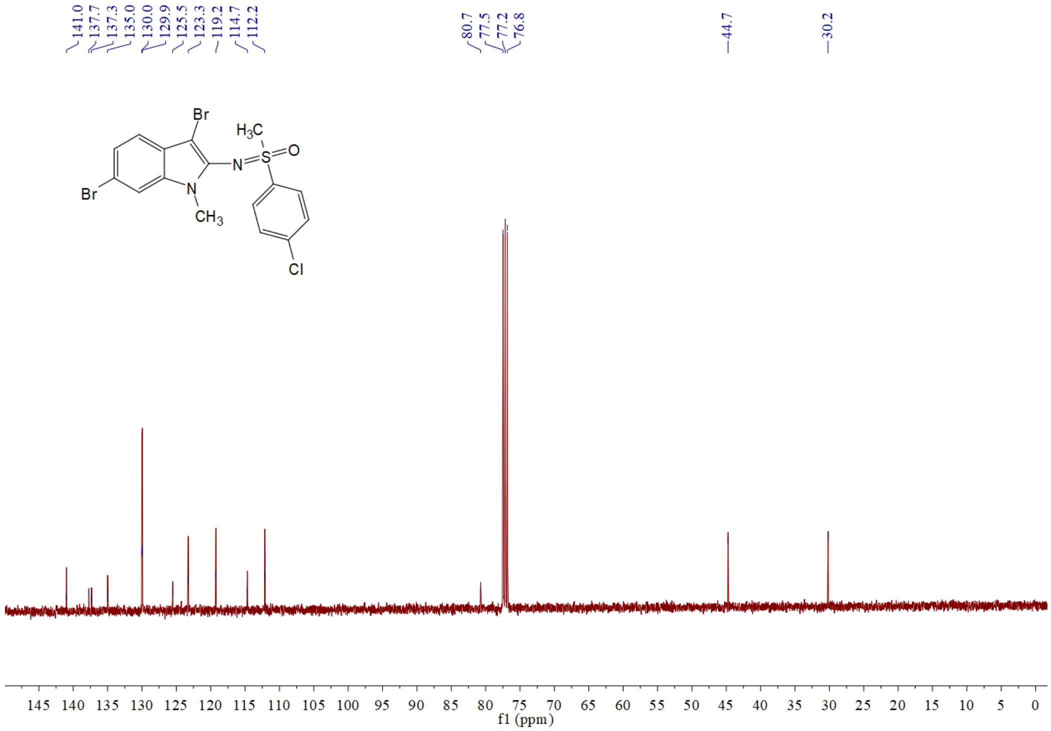
141.0
137.7
137.3
135.0
130.0
129.9
125.5
123.3
119.2
114.7
112.2
80.7
77.5
77.2
76.8
44.7
30.2
Br
H3C
N
S
O
Br
N
CH3
Cl
145 140 135 130 125 120 115 110 105 100 95 90 85 80 75 70 65 60 55 50 45 40 35 30 25 20 15 10 5 0
f1 (ppm)

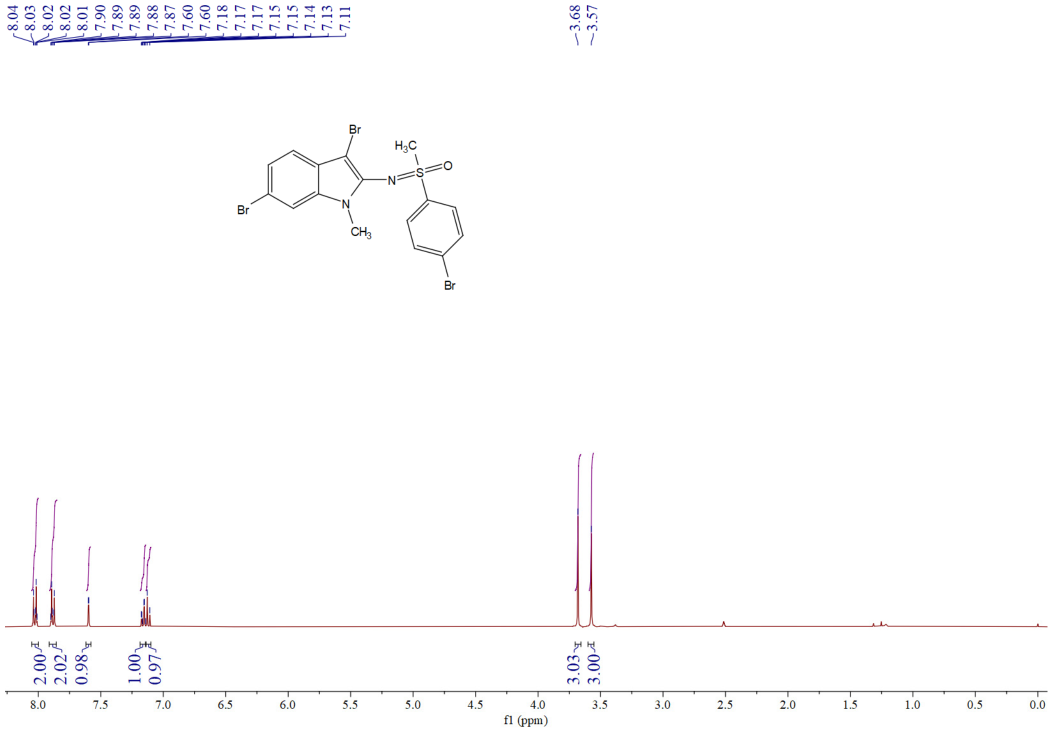
8.04
8.03
8.02
8.02
8.01
7.90
7.89
7.89
7.88
7.87
7.60
7.60
7.18
7.17
7.17
7.15
7.15
7.14
7.13
7.11
3.68
3.57
Br
H3C
N
S
O
Br
N
CH3
Br
2.00
2.02
0.98
1.00
0.97
3.03
3.00
f1 (ppm)

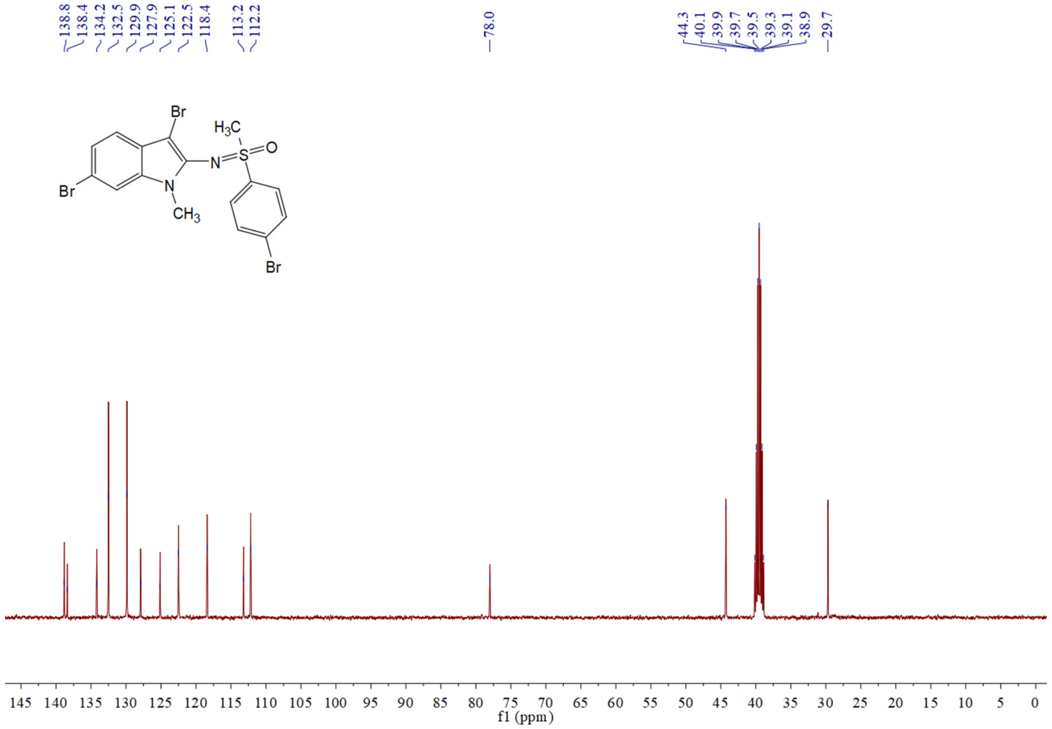
138.8
138.4
134.2
132.5
129.9
127.9
125.1
122.5
118.4
113.2
112.2
78.0
44.3
40.1
39.9
39.7
39.5
39.3
39.1
38.9
29.7
Br
H3C
N
S
O
Br
N
CH3
Br
f1 (ppm)

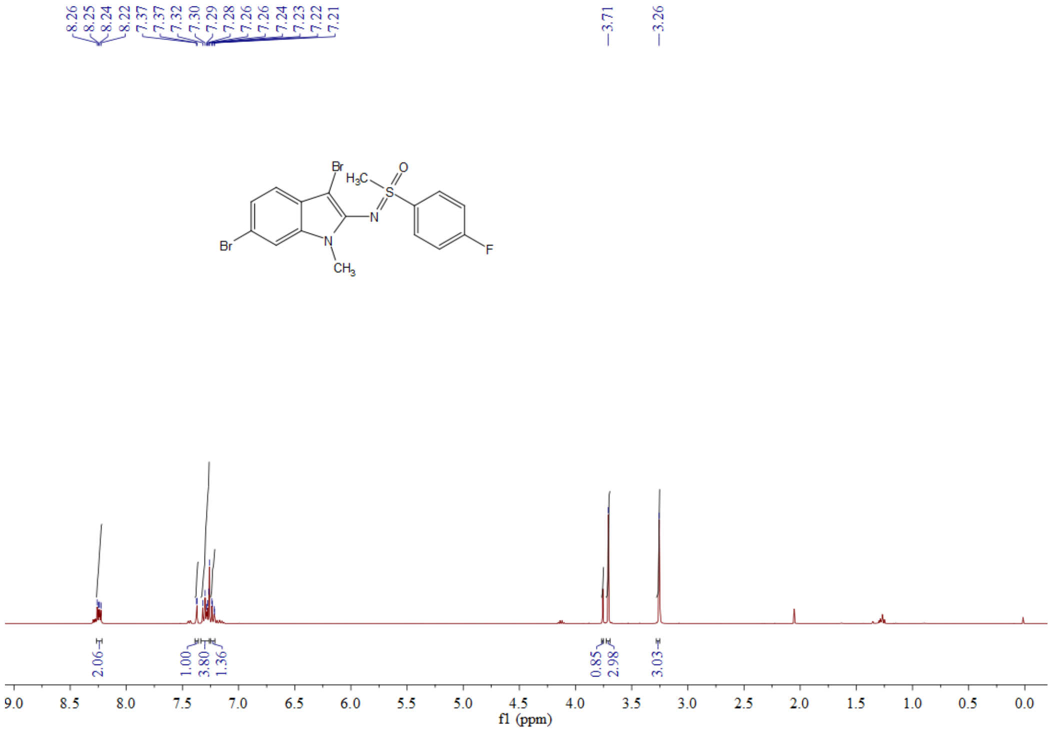
8.26
8.25
8.24
8.22
7.37
7.37
7.32
7.30
7.29
7.28
7.26
7.26
7.24
7.23
7.22
7.21
3.71
3.26
Br
H_3C
O
S
N
Br
N
CH_3
F
2.06
1.00
3.80
1.36
0.85
2.98
3.03
f1 (ppm)

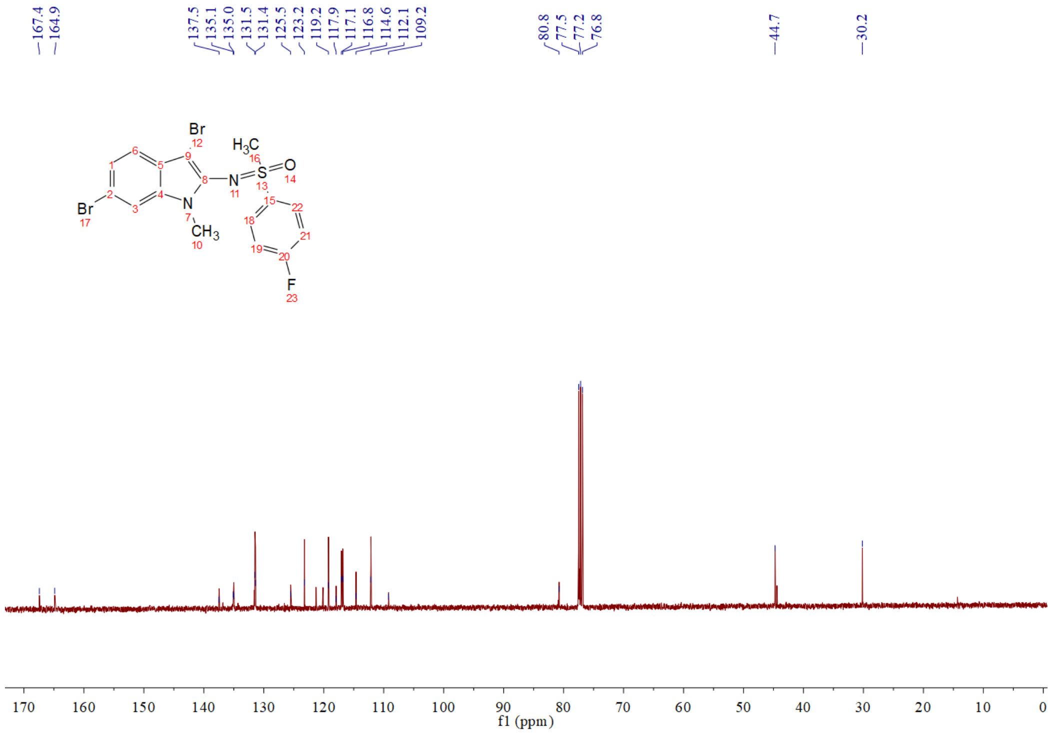
167.4
164.9
137.5
135.1
135.0
131.5
131.4
125.5
123.2
119.2
117.9
117.1
116.8
114.6
112.1
109.2
80.8
77.5
77.2
76.8
44.7
30.2
Br
12
H_3C
16
O
14
S
13
N
11
Br
17
N
7
CH_3
10
F
23
f1 (ppm)

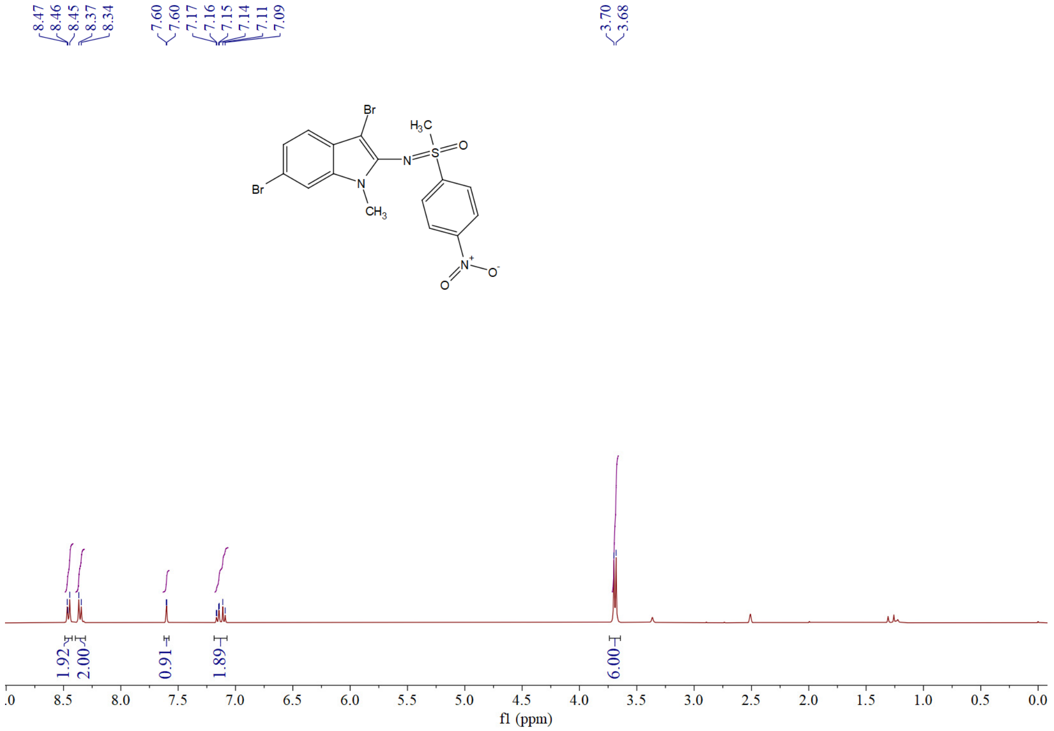
8.47
8.46
8.45
8.37
8.34
7.60
7.60
7.17
7.16
7.15
7.14
7.11
7.09
3.70
3.68
Br
H3C
N
S
O
Br
N
CH3
N+
O
O-
1.92
2.00
0.91
1.89
6.00
f1 (ppm)

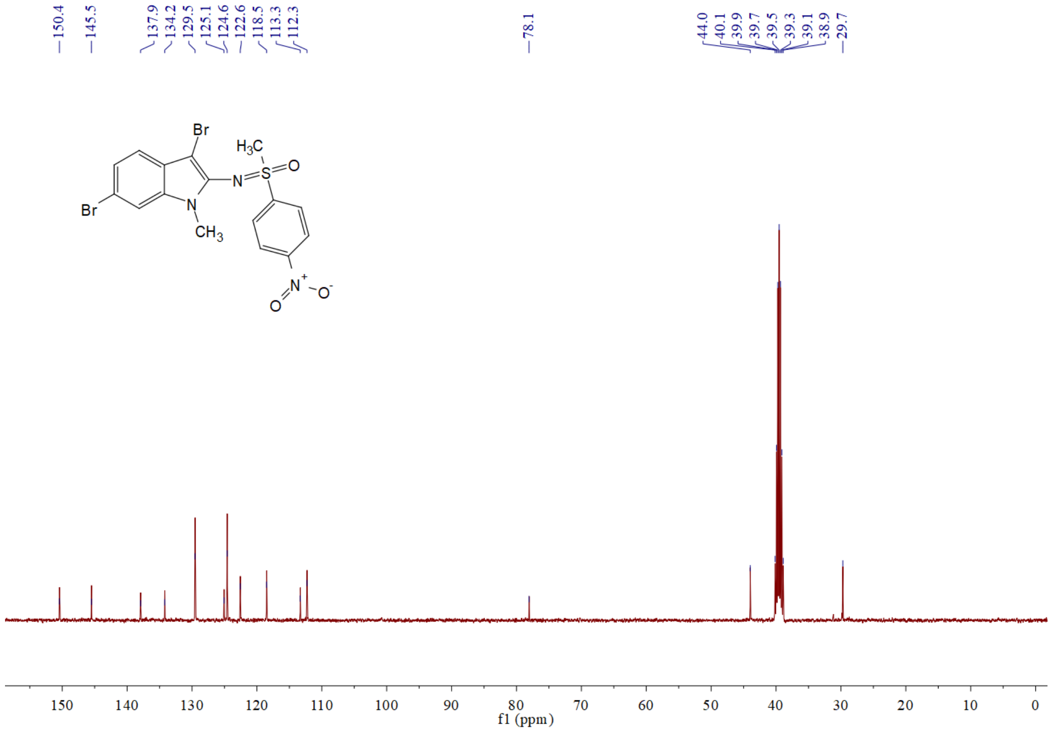
150.4
145.5
137.9
134.2
129.5
125.1
124.6
122.6
118.5
113.3
112.3
78.1
44.0
40.1
39.9
39.7
39.5
39.3
39.1
38.9
29.7
Br
H3C
N
S
O
Br
N
CH3
N+
O
O-
f1 (ppm)

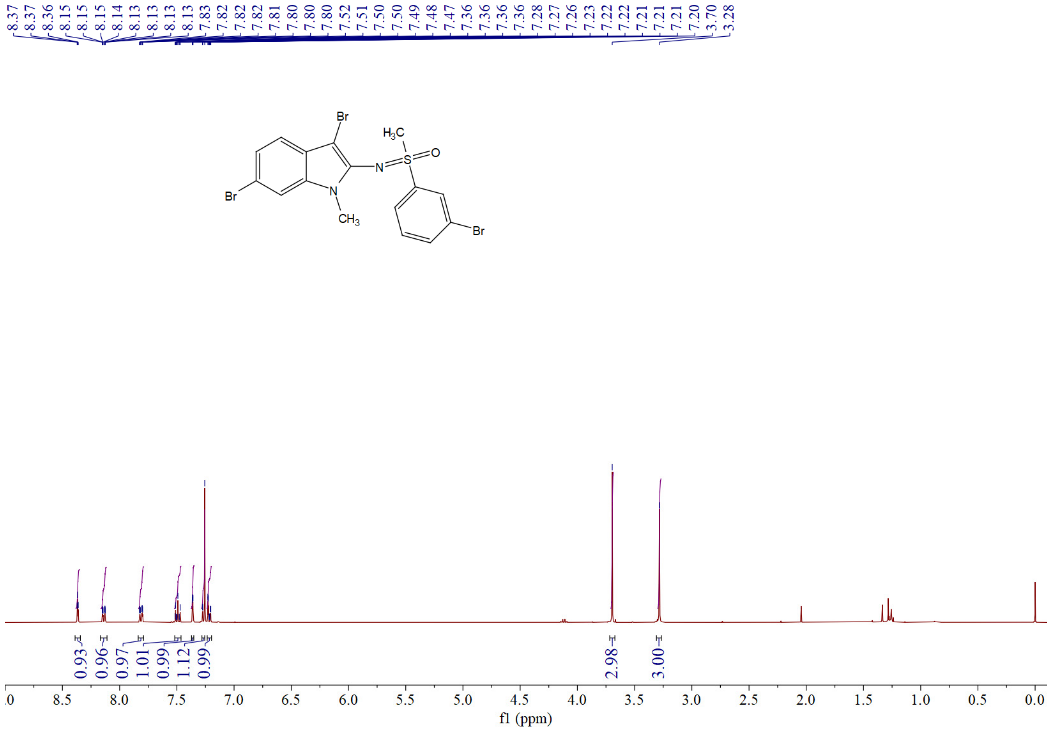

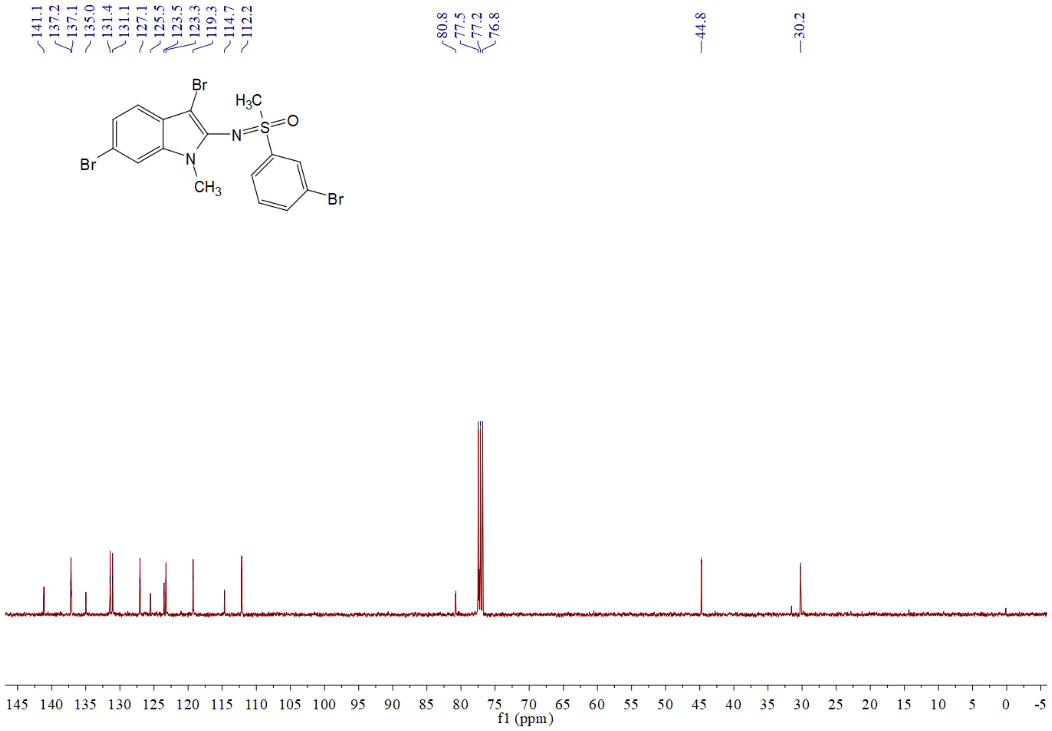

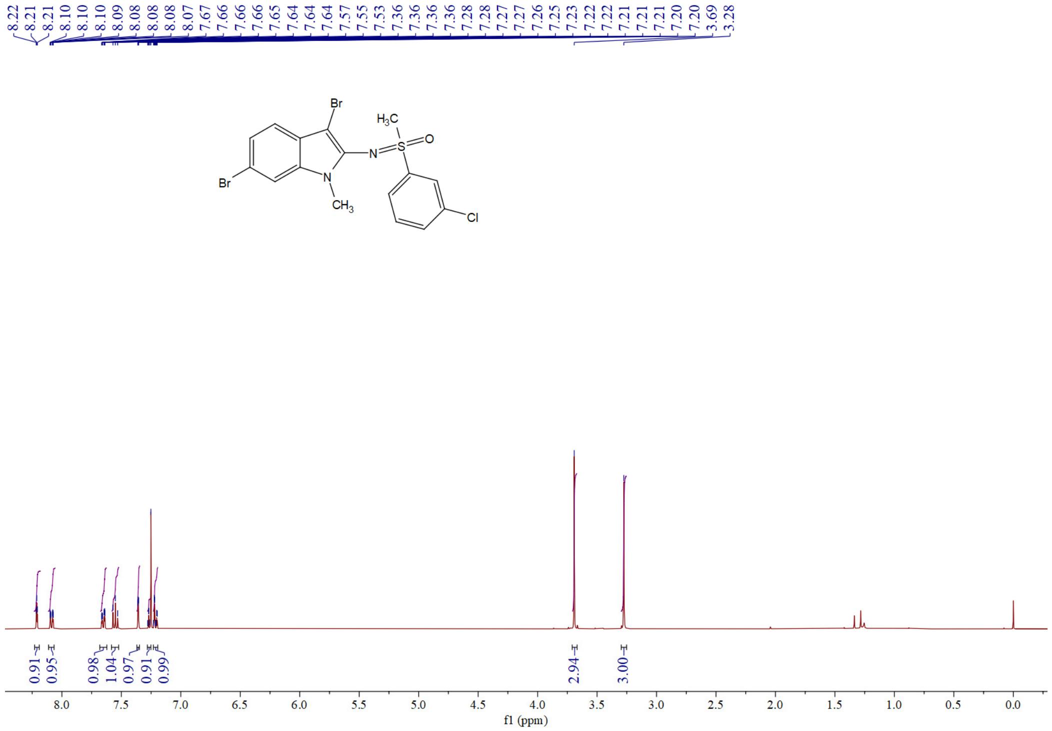
8.22 8.21 8.21 8.10 8.10 8.10 8.09 8.08 8.08 8.08 8.07 7.67 7.66 7.66 7.66 7.65 7.64 7.64 7.64 7.57 7.55 7.53 7.36 7.36 7.36 7.36 7.28 7.28 7.27 7.27 7.26 7.25 7.23 7.22 7.22 7.21 7.21 7.21 7.20 7.20 3.69 3.28
Br
H3C
N
S
O
Br
N
CH3
Cl
0.91 0.95 0.98 1.04 0.97 0.91 0.99 2.94 3.00
f1 (ppm)

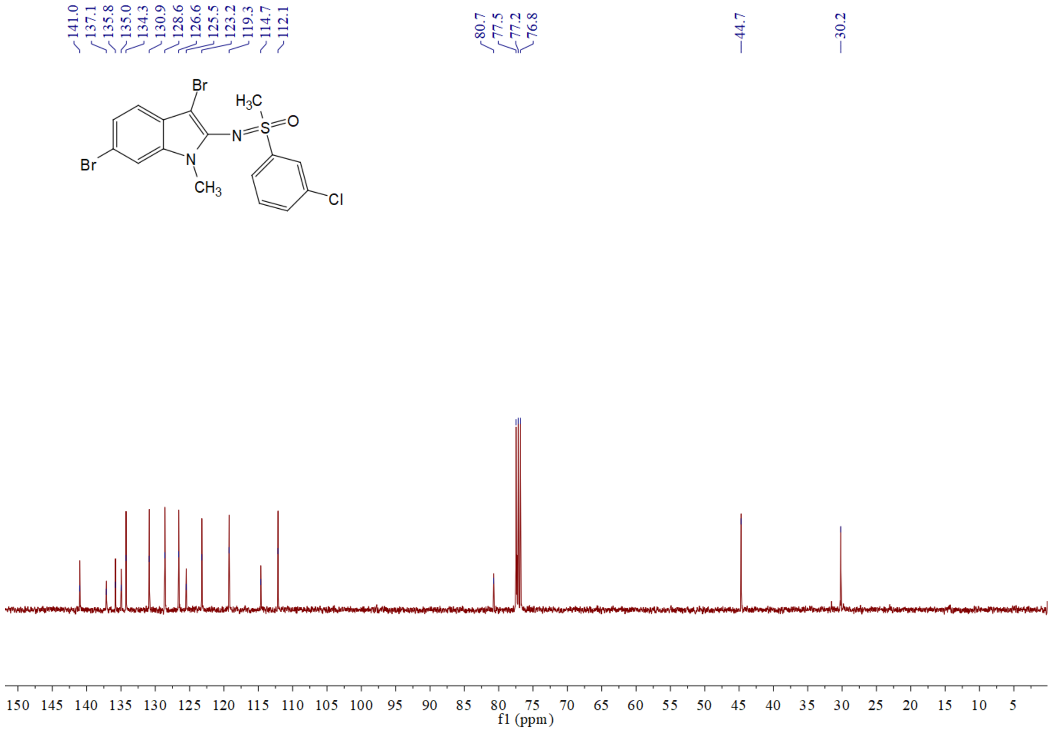
141.0 137.1 135.8 135.0 134.3 130.9 128.6 126.6 125.5 123.2 119.3 114.7 112.1
80.7 77.5 77.2 76.8
44.7
30.2
Br
H3C
N
S
O
Br
N
CH3
Cl
f1 (ppm)

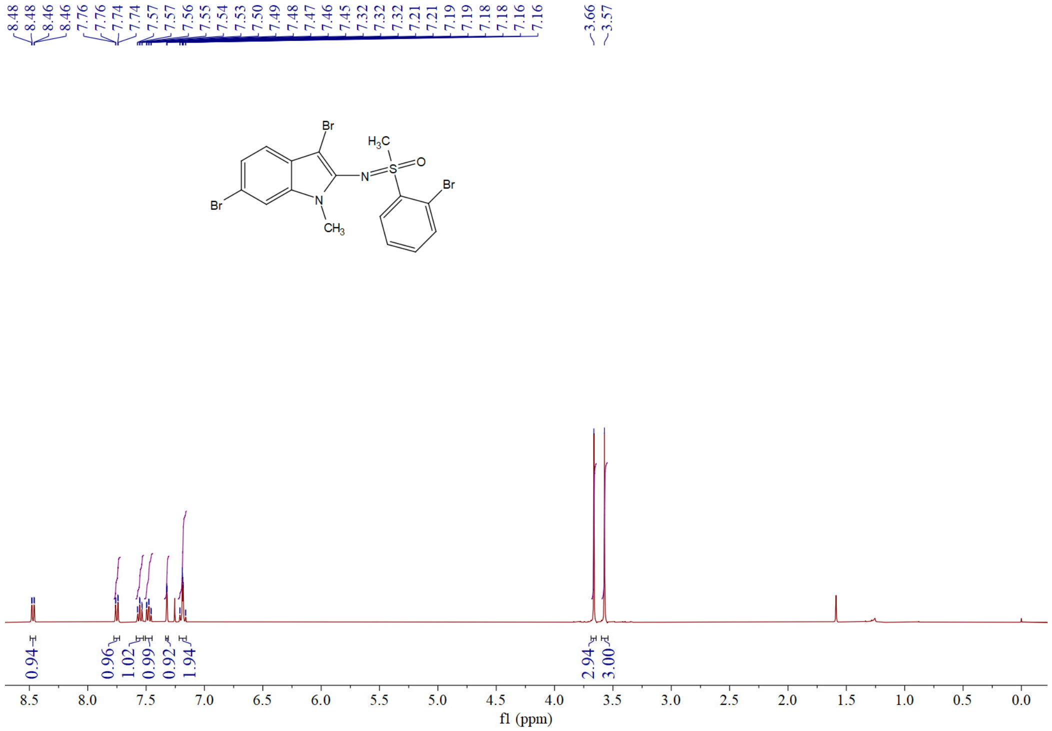
8.48
8.48
8.46
8.46
7.76
7.76
7.74
7.74
7.57
7.57
7.56
7.55
7.54
7.53
7.50
7.49
7.48
7.47
7.46
7.45
7.32
7.32
7.32
7.21
7.21
7.19
7.19
7.18
7.18
7.16
7.16
3.66
3.57
Br
H3C
N
S
O
Br
Br
N
CH3
0.94
0.96
1.02
0.99
0.92
1.94
2.94
3.00
f1 (ppm)

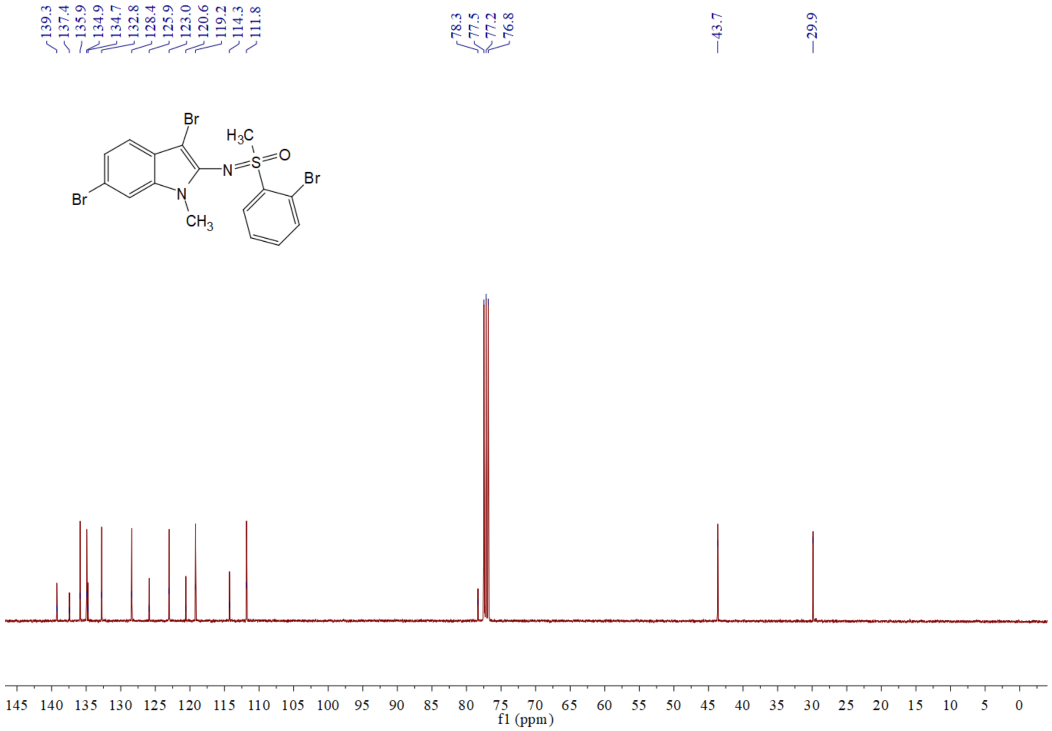
139.3
137.4
135.9
134.9
134.7
132.8
128.4
125.9
123.0
120.6
119.2
114.3
111.8
78.3
77.5
77.2
76.8
43.7
29.9
Br
H3C
N
S
O
Br
Br
N
CH3
f1 (ppm)

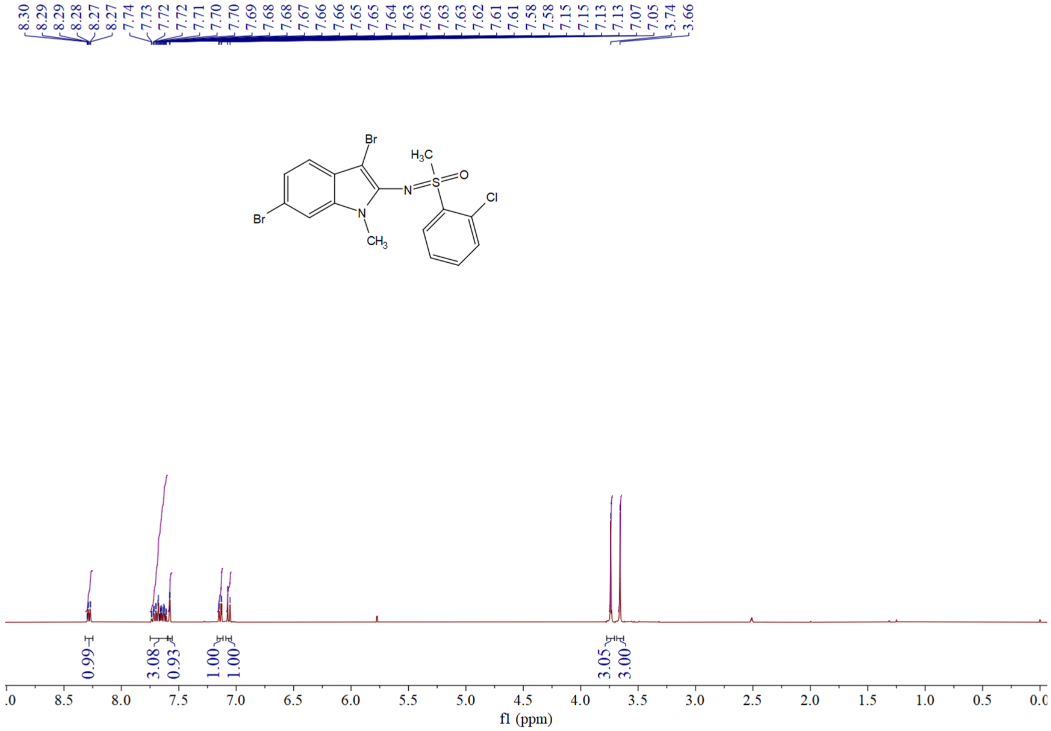
8.30 8.29 8.29 8.28 8.27 8.27 7.74 7.73 7.72 7.72 7.71 7.70 7.70 7.69 7.68 7.68 7.67 7.66 7.66 7.65 7.65 7.64 7.63 7.63 7.63 7.63 7.62 7.61 7.61 7.58 7.58 7.15 7.15 7.13 7.13 7.07 7.05 3.74 3.66
Br
H_3C
N
S
O
Cl
Br
N
CH_3
0.99
3.08
0.93
1.00
1.00
3.05
3.00
f1 (ppm)

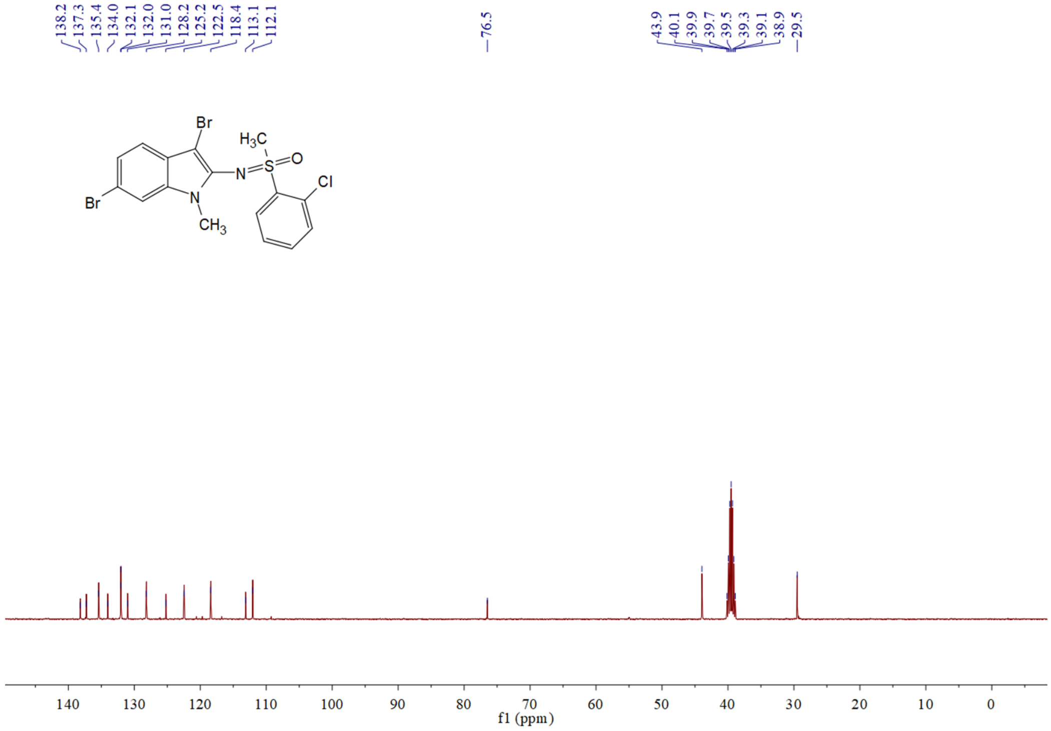
138.2 137.3 135.4 134.0 132.1 132.0 131.0 128.2 125.2 122.5 118.4 113.1 112.1
76.5
43.9 40.1 39.9 39.7 39.5 39.3 39.1 38.9 29.5
Br
H_3C
N
S
O
Cl
Br
N
CH_3
f1 (ppm)

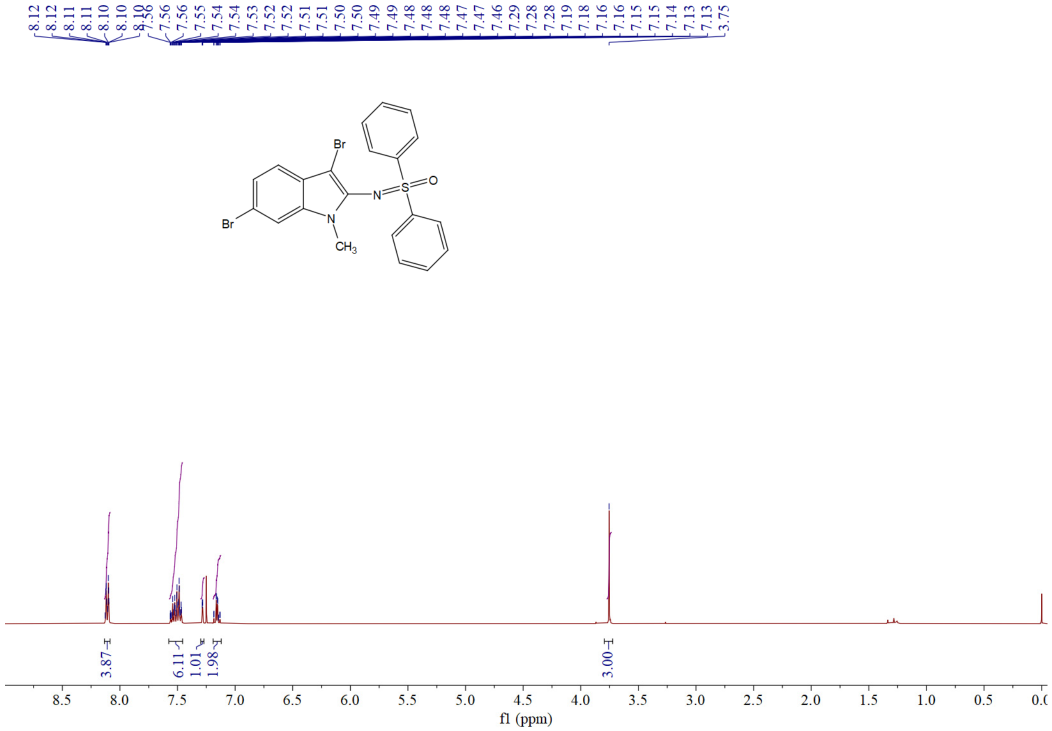
Br
Br
N
CH3
N
S
O
3.87
6.11
1.01
1.98
3.00
f1 (ppm)

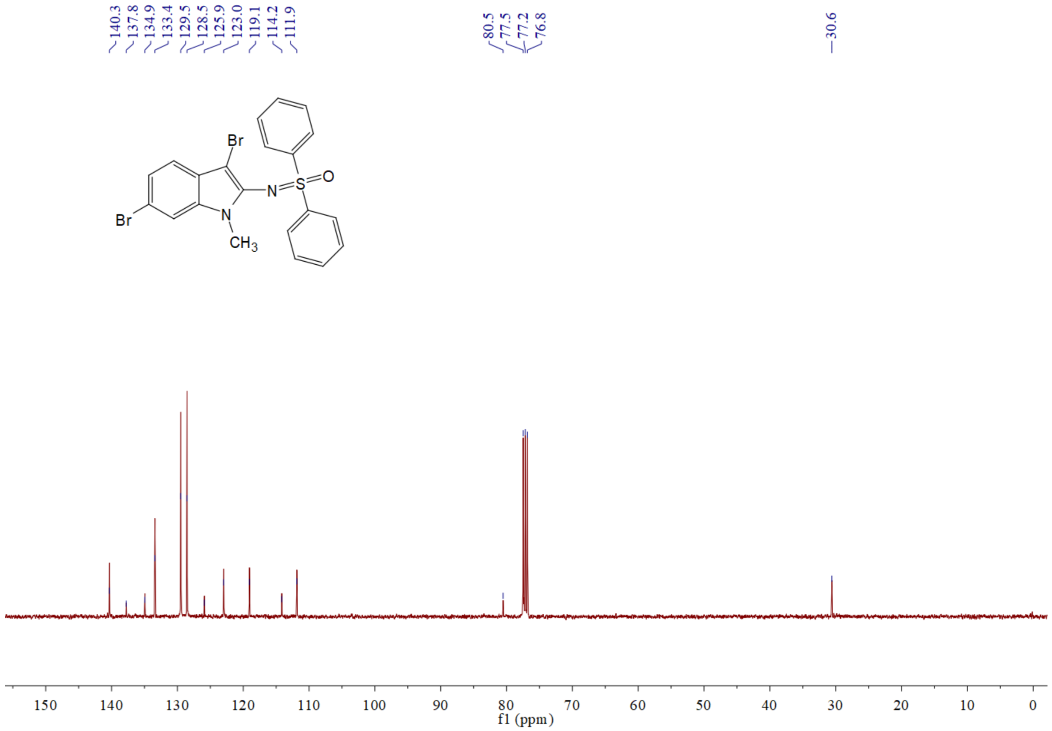
140.3
137.8
134.9
133.4
129.5
128.5
125.9
123.0
119.1
114.2
111.9
80.5
77.5
77.2
76.8
30.6
Br
Br
N
CH3
N
S
O
f1 (ppm)

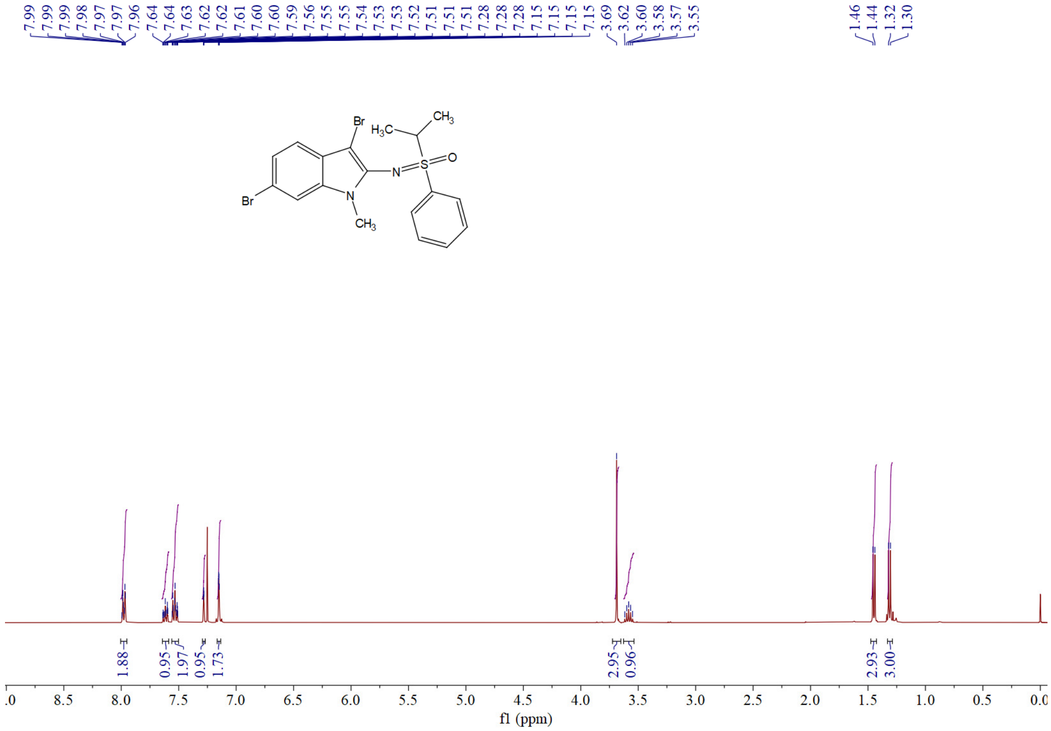
7.99 7.99 7.99 7.98 7.97 7.97 7.96 7.64 7.64 7.63 7.62 7.62 7.61 7.60 7.60 7.59 7.56 7.55 7.55 7.54 7.53 7.53 7.52 7.51 7.51 7.51 7.28 7.28 7.28 7.15 7.15 7.15 7.15 3.69 3.62 3.60 3.58 3.57 3.55
1.46 1.44 1.32 1.30
Br
H_3C
CH_3
N
S
O
Br
N
CH_3
1.88
0.95
1.97
0.95
1.73
2.95
0.96
2.93
3.00
f1 (ppm)

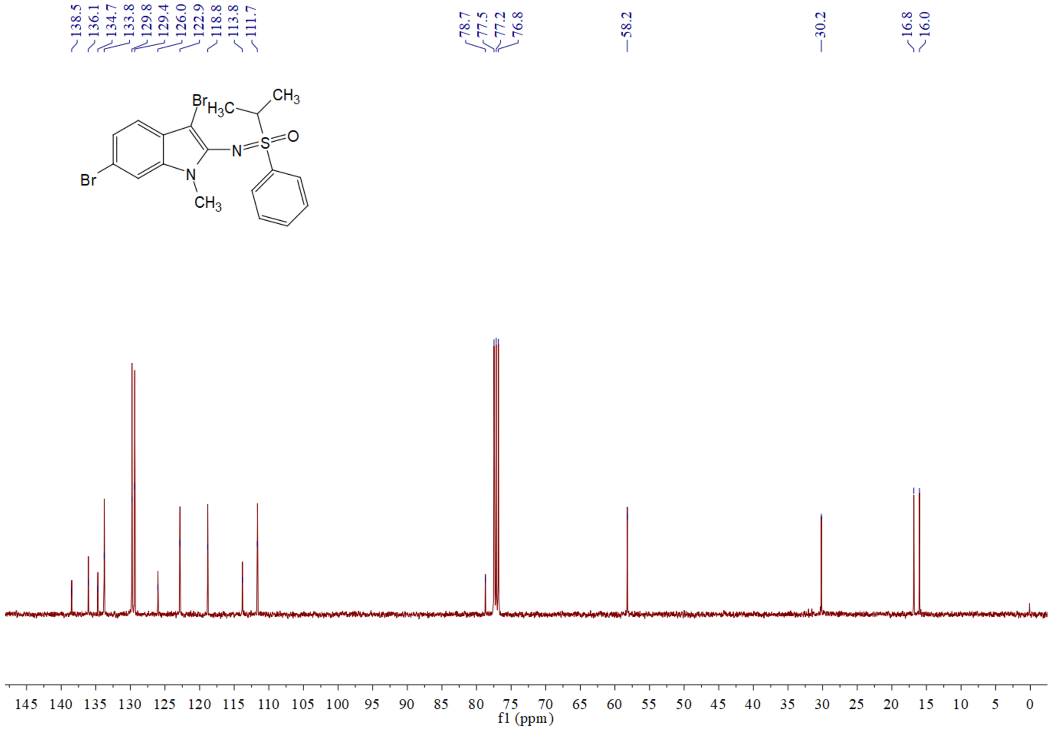
138.5 136.1 134.7 133.8 129.8 129.4 126.0 122.9 118.8 113.8 111.7
78.7 77.5 77.2 76.8
58.2
30.2
16.8 16.0
Br
H_3C
CH_3
N
S
O
Br
N
CH_3
f1 (ppm)

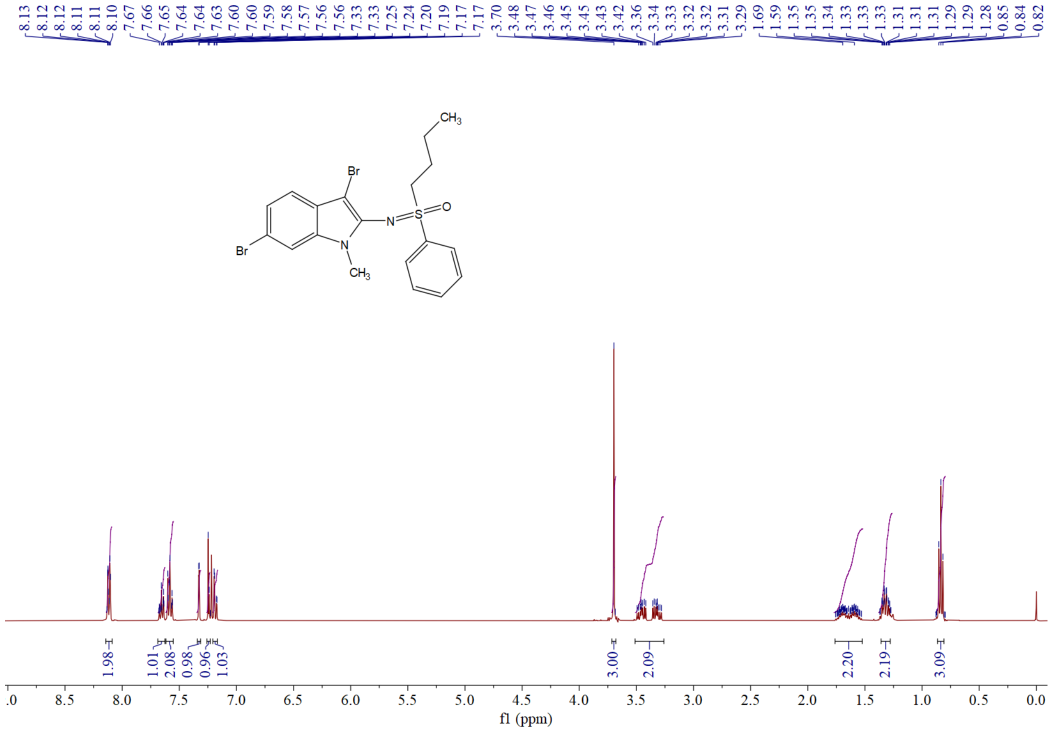
8.13 8.12 8.12 8.11 8.11 8.10 7.67 7.66 7.65 7.64 7.64 7.63 7.60 7.60 7.59 7.58 7.57 7.56 7.56 7.33 7.33 7.25 7.24 7.20 7.19 7.17 7.17 3.70 3.48 3.47 3.46 3.45 3.45 3.43 3.42 3.36 3.34 3.33 3.32 3.32 3.31 3.29 1.69 1.59 1.35 1.35 1.34 1.33 1.33 1.33 1.31 1.31 1.31 1.29 1.29 1.28 0.85 0.84 0.82
CH3
Br
N
S
O
Br
N
CH3
1.98 1.01 2.08 0.98 0.96 1.03 3.00 2.09 2.20 2.19 3.09
.0 8.5 8.0 7.5 7.0 6.5 6.0 5.5 5.0 4.5 4.0 3.5 3.0 2.5 2.0 1.5 1.0 0.5 0.0
f1 (ppm)

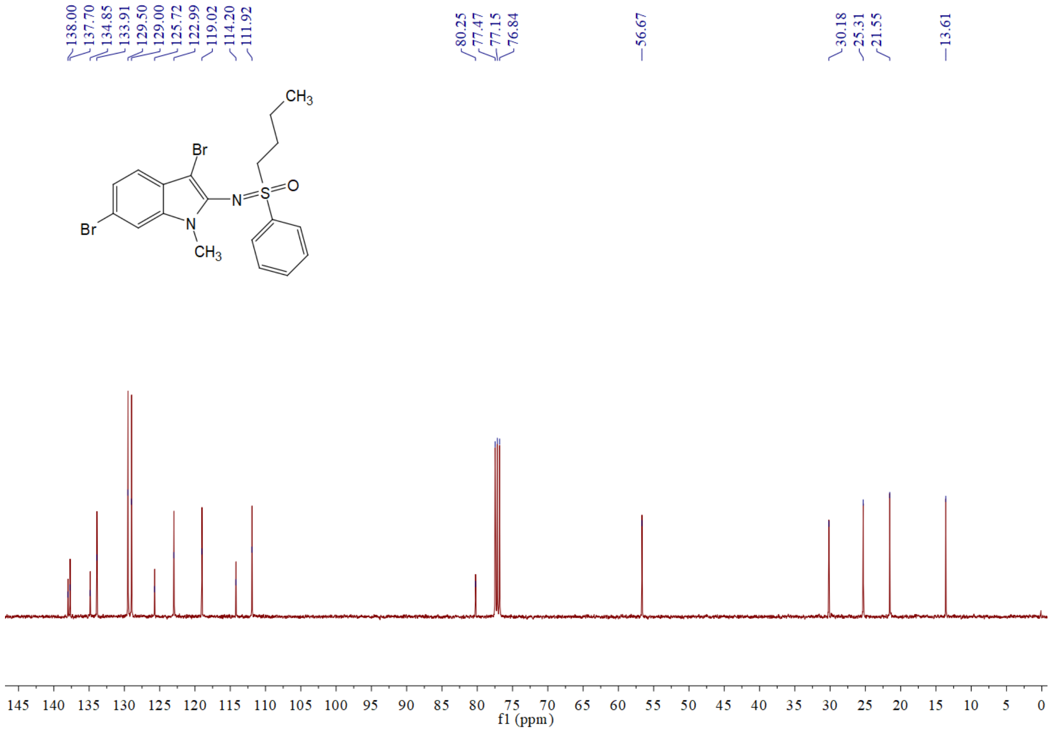
138.00 137.70 134.85 133.91 129.50 129.00 125.72 122.99 119.02 114.20 111.92
80.25 77.47 77.15 76.84
56.67
30.18 25.31 21.55
13.61
CH3
Br
N
S
O
Br
N
CH3
145 140 135 130 125 120 115 110 105 100 95 90 85 80 75 70 65 60 55 50 45 40 35 30 25 20 15 10 5 0
f1 (ppm)

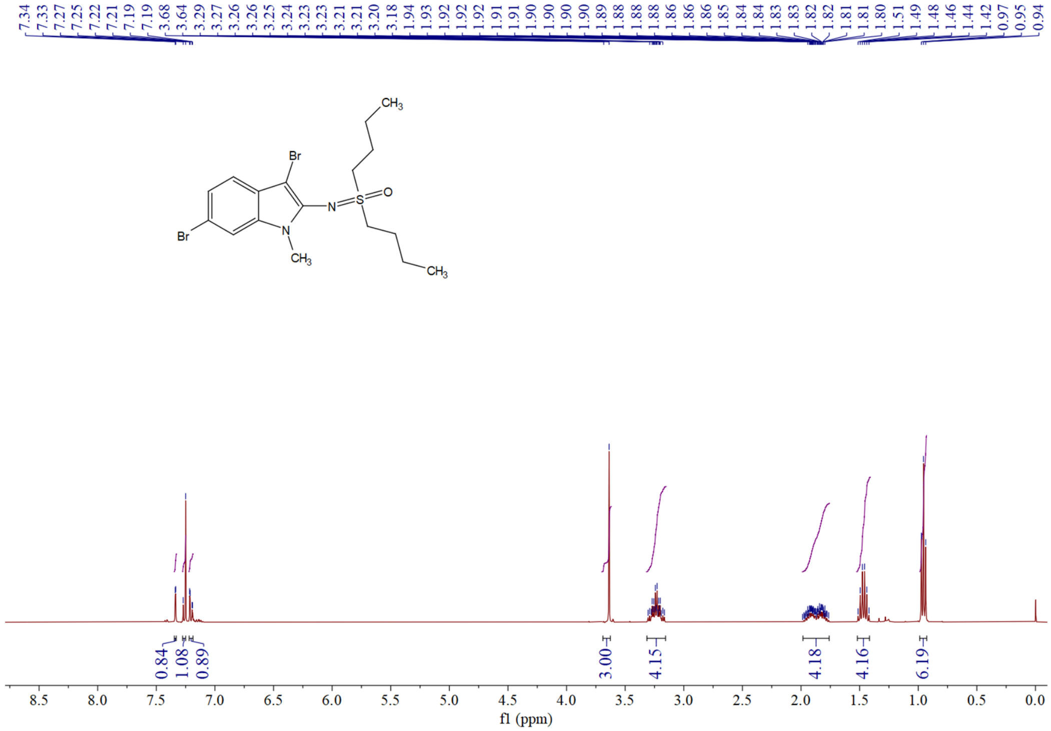
7.34 7.33 7.27 7.25 7.22 7.21 7.19 7.19 3.68 3.64 3.29 3.27 3.26 3.25 3.24 3.23 3.23 3.21 3.21 3.20 3.18 1.94 1.93 1.92 1.92 1.92 1.91 1.91 1.90 1.90 1.90 1.89 1.88 1.88 1.88 1.86 1.86 1.85 1.84 1.84 1.83 1.83 1.82 1.82 1.81 1.81 1.80 1.51 1.49 1.48 1.46 1.44 1.42 0.97 0.95 0.94
CH3
Br
N
S
O
Br
N
CH3
CH3
0.84
1.08
0.89
3.00
4.15
4.18
4.16
6.19
8.5 8.0 7.5 7.0 6.5 6.0 5.5 5.0 4.5 4.0 3.5 3.0 2.5 2.0 1.5 1.0 0.5 0.0
f1 (ppm)

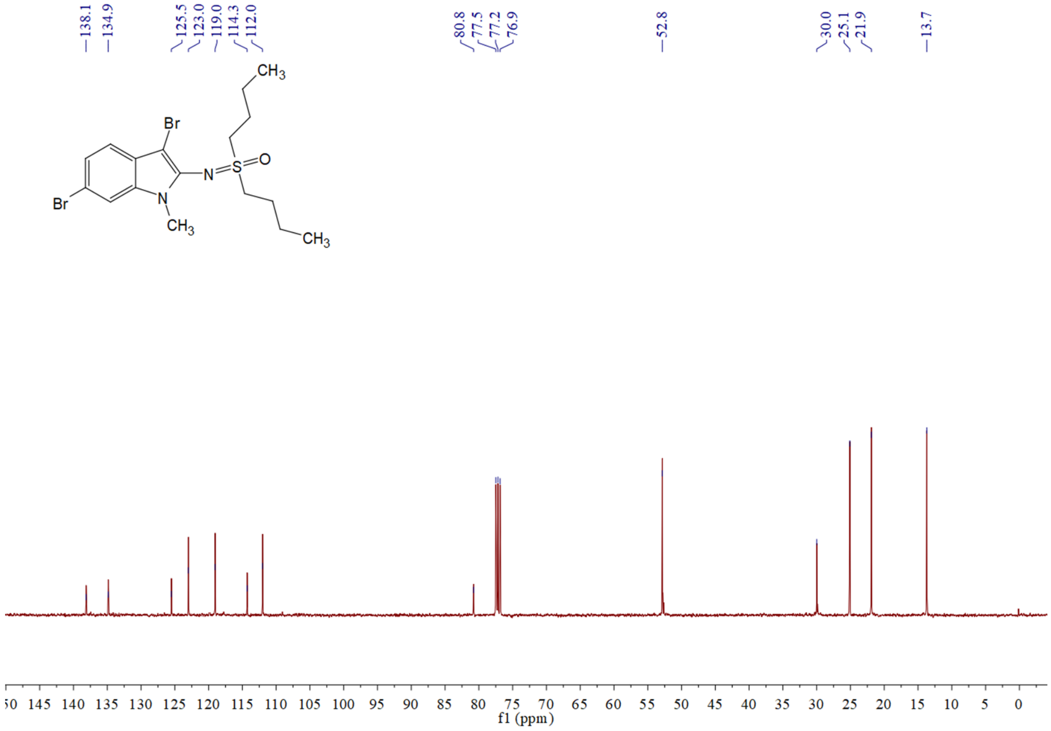
138.1 134.9 125.5 123.0 119.0 114.3 112.0 80.8 77.5 77.2 76.9 52.8 30.0 25.1 21.9 13.7
CH3
Br
N
S
O
Br
N
CH3
CH3
145 140 135 130 125 120 115 110 105 100 95 90 85 80 75 70 65 60 55 50 45 40 35 30 25 20 15 10 5 0
f1 (ppm)
